# Microplastics‐Induced Gut Microbiota Dysbiosis Accelerates Alzheimer's‐Like Pathology and Cognitive Decline via the Gut–Brain Axis

**DOI:** 10.1002/advs.76072

**Published:** 2026-06-15

**Authors:** Zifeng Wu, Jiarong Yang, Miaoxuan Zhang, Yi Li, Zhuling Tu, Ji Wang, Jin‐Tai Yu, Kun Guo, Rongcan Luo

**Affiliations:** ^1^ Gansu Key Laboratory of Biomonitoring and Bioremediation for Environmental Pollution and Ministry of Education Key Laboratory of Cell Activities and Stress Adaptations School of Life Sciences Lanzhou University Lanzhou China; ^2^ Department of Neurology National Center for Neurological Disorders Huashan Hospital, State Key Laboratory of Medical Neurobiology MOE Frontiers Center for Brain Science Shanghai Medical College Fudan University Shanghai China; ^3^ Department of Anatomy and Histology & Embryology Faculty of Basic Medical Science Kunming Medical University Kunming China

**Keywords:** Aβ pathology, Alzheimer's disease, autophagy, fecal microbiota transplantation, gut microbiota, metabolome, microplastics, Taurine

## Abstract

Alzheimer's disease (AD) is incurable and increasingly attributed to gene–environment interactions. Microplastics (MPs) are omnipresent in the human food chain, yet their impact on neurodegeneration is largely unknown. Here we show that chronic oral exposure to 2‐µm amine‐modified polystyrene microparticles accelerates cognitive decline, amplifies Aβ deposition, gliosis, and synaptic loss, and cripples autophagic flux in 5XFAD mice through the gut–brain axis. MPs accumulate in the gut, breach the epithelial barrier, and selectively expand the taurine‐depleting pathobiont *Bilophila*, while suppressing taurine‐synthesizing commensals. Untargeted metabolomics reveal a systemic taurine deficit that precedes and predicts exacerbated Aβ deposition, gliosis, synaptic loss, and autophagic blockade in 5XFAD mice. Antibiotic‐mediated microbiota ablation and fecal microbiota transplantation (FMT) demonstrate that the neurotoxic phenotype is fully microbiota‐dependent. Restoring taurine level rebalances microglial homeostasis, reinstates autophagic flux, and rescues memory deficits in MPs‐treated 5XFAD mice. Translational validation using Alzheimer's Disease Neuroimaging Initiative (ADNI) plasma shows taurine is significantly lower in AD patients versus cognitively normal controls and inversely correlates with cognitive decline. Our findings identify MPs‐induced gut‐microbiota dysbiosis as a modifiable environmental driver of AD pathogenesis and establish taurine supplementation as a readily translatable intervention that simultaneously fortifies the intestinal barrier and neutralizes microbiota‐mediated neurodegeneration.

## Graphical abstract

Microplastics (MPs) breach the gut‐brain axis by triggering taurine‐depleting microbiota dysbiosis that accelerates Alzheimer‐like pathology in 5XFAD mice. Taurine supplementation normalizes autophagy, microglial quiescence, and cognition, and plasma taurine inversely tracks cognitive decline in ADNI participants, revealing a readily translatable metabolite target.

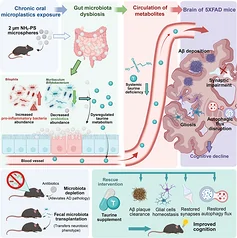

## 1 Introduction

Alzheimer's disease (AD) is an age‐associated, progressive neurodegenerative disorder characterized by a decline in cognitive and memory impairment, representing the leading cause of dementia worldwide [1, 2, 3]. The classic pathological hallmarks of AD are the progressive accumulation of extracellular amyloid‐β (Aβ) plaques and intracellular tau pathology. These processes serve as the primary drivers leading to widespread gliosis (both in the microglia and astrocytes) and neuroinflammation, synaptic dysfunction, neuronal loss, and impaired autophagic flux in the whole brain, and ultimately cognitive decline [4, 5, 6]. In recent years, substantial efforts have been devoted to the development of disease‐modifying therapies for AD, and several pharmacological agents and therapeutic strategies have advanced into late‐stage clinical trials, with some receiving regulatory approval. These advances mark important milestones in AD research [7]. However, to date, no available therapy has been demonstrated to fully halt, reverse, or even cure the disease, and clinical benefits remain modest, with considerable heterogeneity across patient populations [6, 8, 9, 10]. Consequently, there remains a critical unmet need to identify additional biological pathways and modifiable factors that shape AD onset and progression, particularly those operating upstream of overt neurodegeneration. The limited efficacy observed in recent clinical trials suggests that AD pathogenesis likely reflects a complex, system‐level disturbance engaging multiple biological pathways and regulatory networks, rather than a single linear cascade driven by an isolated molecular target [11].

In recent years, accumulating evidence has indicated that sporadic late‐onset AD (LOAD) represents over 90% of Alzheimer's cases and is driven by the dynamic interaction between genetic elements factors and environmental risk factors, including levels of education, lifestyle‐associated factors, and specifically chronic exposures to environmental toxicants, which may interact with aging‐related vulnerability to shape disease trajectory [12, 13]. In parallel with these emerging concepts of environmental factors, microplastics (MPs) have gained increasing attention as a pervasive and persistent component of the modern exposome [14, 15, 16]. MPs are particles that are smaller than 5 mm in diameter, and they can also include smaller nanoplastics (less than 1 µm). The presence of MPs in the environment has become a significant global concern [14, 17, 18] due to the potential risks they pose to human health [19, 20, 21]. Due to the small size and complex morphology, MPs are now widely distributed across terrestrial [22] and aquatic environments [23], and are continuously encountered through routine human activities [17, 24, 25]. It has been reported that MPs have been detected in a range of human samples, including stool, tissues, organs, and bodily fluids in recent years [26, 27, 28]. Specifically, owing to their small size and chemical stability, ingested MPs have a deleterious effect on human health [18, 20, 29, 30]. Emerging evidence positions MPs as a potential risk factor that can aggravate pathological features in neurodegenerative diseases [16], notably in Parkinson's disease (PD) [30, 31] and amyotrophic lateral sclerosis (ALS) [32]. A previous study showed that nanoplastics promote Aβ peptide aggregation in vitro [33]. However, direct experimental evidence demonstrating a causal role of MPs in aggravating AD‐related pathology remains lacking.

Given that ingested MPs primarily accumulate in the gastrointestinal tract, it is plausible that they exert systemic toxicity through intestinal pathways [14]. Multiple studies have suggested that the gut microbiota plays a pivotal role in mediating the systemic toxicity exacerbated by MPs exposure [34, 35, 36]. Meanwhile, gut microbiota plays an essential role in regulating central nervous system functions, including neuronal development, neural signaling, and behavior [37]. The composition of the gut microbiota of patients with AD differs from that of healthy individuals [38]. Recent evidence suggests a potential role for gut microbiota in the regulation of AD‐related brain Aβ pathology and cognitive impairment [39]. Consistent with this, a relevant study indicates that antibiotic (ABX) treatment can ameliorate AD pathological progression [40] and that fecal microbiota transplantation (FMT) from AD patients can induce AD‐like phenotypes in recipient mice [39, 41]. Collectively, these lines of evidence position the gut as a critical target for MPs and implicate gut microbiota as a key mediator in both MPs toxicity and AD pathogenesis. However, the specific mechanisms by which environmental MPs might exploit this gut‐microbiota‐brain interface to influence neurodegenerative processes remain poorly understood. Deciphering the molecular mechanism by which MPs affect AD can help in understanding the pathophysiology of AD and exploiting the new therapeutics.

In this article, we investigated the contribution of MPs to the AD pathogenesis through their interaction with the gut microbiota. We found that oral MPs treatment exacerbated cognitive deficits and key neuropathological hallmarks in 5XFAD mice, a model of AD. Further metabolomic profiling revealed that MPs significantly disrupted the serum metabolome of the 5XFAD mice. This systemic metabolic disruption was found to be associated with concurrent dysbiosis of the gut microbiota and perturbation of the cecal metabolome. Interestingly, supplementation with specific depleted endogenous metabolite rescued the MPs‐aggravated AD phenotypes, including the cognitive decline, Aβ burden, and glial overactivation. Our findings thus demonstrate that MPs are a potential causative factor to promote AD progression, at least through the gut‐microbiota‐metabolites‐brain axis, and emphasize the crucial need to address plastic pollution globally.

## 2 Results

### 2.1 MPs Exacerbated the Behavioral Deficits in 5XFAD Mice

To ensure the consistent colloidal stability throughout the entire treatment window, we first benchmarked the physicochemical identity of the selected 2‐µm amine‐modified polystyrene microparticles [42]. Scanning electron microscopy (SEM) revealed that most of the particles with a regular shape were closely and orderly packed together (Figure S1A), while nearly all MPs exhibited a particle diameter ranging from 1.90 to 2.05 µm, with the highest distribution frequency observed in the range of 1.95 – 2.00 µm (Figure S1B). Dynamic light scattering (DLS) was performed to analyze the hydrodynamic particle size distribution in the freshly prepared working solution (Figure S1C). DLS analysis showed a consecutive pattern of diameter change between 1874.87 and 2184.88 nm, with the most frequency of diameter at 2041.84 nm (Intensity = 18.53%). In addition, three characteristic absorption peaks were observed in the Fourier transform infrared spectroscopy (FTIR) of the microplastic particles (Figure S1D): (1) The absorption band at 1100 cm^−1^ was assigned to the asymmetric stretching of the C‐N bond; (2) The absorption band at 1733 cm^−1^ was assigned to the asymmetric stretching of the C═O bond; (3) The absorption band at 3420 cm^−1^ was assigned to the asymmetric stretching of the N─H bond. Analysis of the FTIR data indicated that the selected MPs were amine‐modified polystyrene microparticles. The zeta potentials of the MPs were −4.85 ± 0.065 mV (Figure S1E), indicating that the MPs were stable in the working solution.

**FIGURE 1:**
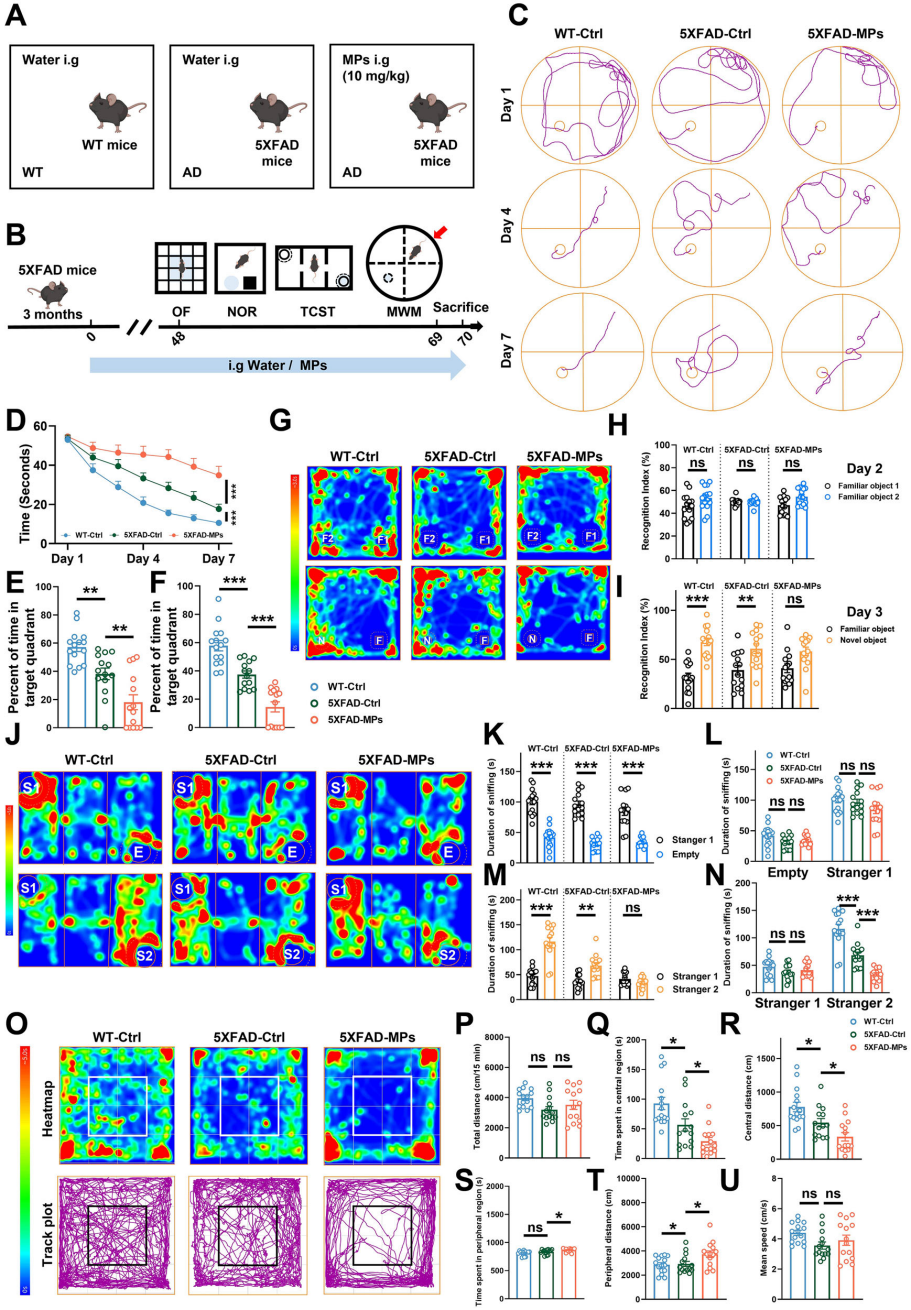
MPs exposure accelerates cognitive and exploratory dysfunction in 5XFAD mice. (A) Grouping and (B) experimental timelines of oral MPs administration followed by behavioral testing. (C) Representative swim paths recorded during the Morris water maze (MWM). (D) MWM navigation training showed escape latency across consecutive training days. (E‐F) Percentage of time spent in the target quadrant of all groups in the probe tests conducted at (E) 4 h and (F) 72 h after the last training session. (G) Representative heatmaps of exploration patterns in the novel‐object recognition (NOR) task. (H‐I) Recognition index (%) on day 2 (familiar vs. familiar; H and G, upper) and day 3 (novel vs. familiar; I and G, lower). (J) Representative heatmaps of sniffing patterns in the three‐chamber social test (TCST). (K) Comparison of duration of sniffing (s) between stranger 1 (S1) and Empty in each group. (L) Comparison of duration of sniffing (s) to Empty or S1 among the three groups both. (M) Comparison of duration of sniffing (s) between S1 and S2 in each group. (N) Comparison of duration of sniffing (s) to S1 or S2 among the three groups both. (O) Representative heatmaps and tracked paths of the Open‐field (OF) test. (P‐U) Comparison of (P) total distance (cm/15 min), (Q) time spent in central region, (R) central distance (cm), (S) time spent in peripheral region (s), (T) peripheral distance (cm) and (U) mean speed (cm/s) among three groups in the OF test. The group sizes were n = 15, 14, and 14 for the WT‐Ctrl, 5XFAD‐Ctrl, and 5XFAD‐MPs groups, respectively. The WT‐Ctrl group included 8 males and 7 females, while the other two groups each included 7 males and 7 females, respectively. Each point represented a mouse. Data are presented as mean ± standard error of the mean (SEM). ns, not significant; ^*^ *P* < 0.05, ^**^ *P* < 0.01, ^***^ *P* < 0.001 (Paired two‐tailed t test in H, I, K, M; one‐way ANOVA with Tukey's *post hoc* test in E, F, L, N, P‐U; Two‐way ANOVA with Tukey's *post hoc* test in D).

To determine the effect of MPs on AD pathology, 3‐month‐old 5XFAD mice received a daily gavage 10 mg kg^−^^1^ body weight MPs or sterile water for 70 days; wild type (WT) littermates served as a negative control (Figure 1A,B). The behavioral tests were performed at the 48th day for 21 days, followed by euthanasia for subsequent experiments (e.g., tissue harvesting, serum collection) (Figure 1A,B). Throughout the MPs treatment period, body weight gain in 5XFAD mice was significantly attenuated, indicating that MPs treatment markedly compromised the overall health status of mice (Figure S2A). The Morris water maze (MWM) consisted of a navigation training phase and 4 and 72 h spatial probe tests and was employed to assess spatial learning and memory in mice. During the navigation training phase, mice from the three groups were trained for seven consecutive days, followed by analysis of escape latency over the training period. Compared with WT mice, 5XFAD mice exhibited a significantly increased escape latency during the seven‐day training period. Notably, MPs treatment further exacerbated this increase, indicating impaired spatial learning and reference memory in 5XFAD mice, which was aggravated by MPs treatment (Figure 1C,D). At 4 h and 72 h after completion of the seven‐day navigation training phase, mice from each group were subjected to identical spatial probe tests. In both probe test 4 h and probe test 72 h, no significant differences in mean swimming speed were observed among the groups (Figure S2F,K). MPs‐treated 5XFAD mice exhibited a significantly prolonged latency to the first platform crossing, accompanied by marked reductions in time spent and distance moved in the target quadrant, as well as in the number of platform crossings (Figure 1E,F, Figure S2B–E, 1G–J). Non‐spatial memory was probed with novel‐object recognition (NOR). During the familiarization, exploration times for identical objects did not differ (Figure 1G,H). In contrast, compared with littermates, 5XFAD mice exhibited a significant reduction in the proportion of time in sniffing the novel object, which was further decreased following MPs treatment (Figure 1G,I). Social cognition was examined in the three‐chamber social test (TCST). All cohorts preferred a stranger mouse (S1) to an empty chamber during the sociability phase (Figure 1J–L). In the subsequent social novelty trial, however, 5XFAD mice exhibited a significantly reduced sniffing time toward the novel mouse (S2) compared with their littermates. Notably, MPs‐treated 5XFAD mice lost the innate preference for the newly introduced stranger (S2), allocating equal sniff time to both stimuli (Figure 1J,M, and N). Considering that anxiety is another phenotype of AD, we further tested whether MPs exacerbated anxiety‐like behaviors in 5XFAD mice. Compared with their littermates, 5XFAD mice showed reduced center time and distance in the open field test (OFT), which were further decreased by MPs treatment, while total locomotor activity remained comparable across groups (Figure 1O–U). Collectively, these results indicate that chronic MPs ingestion contributes to the exacerbation of AD‐associated phenotypes in an Alzheimer's‐relevant model.

### 2.2 MPs Aggravated AD‐Related Pathology in 5XFAD Mice

**FIGURE 2:**
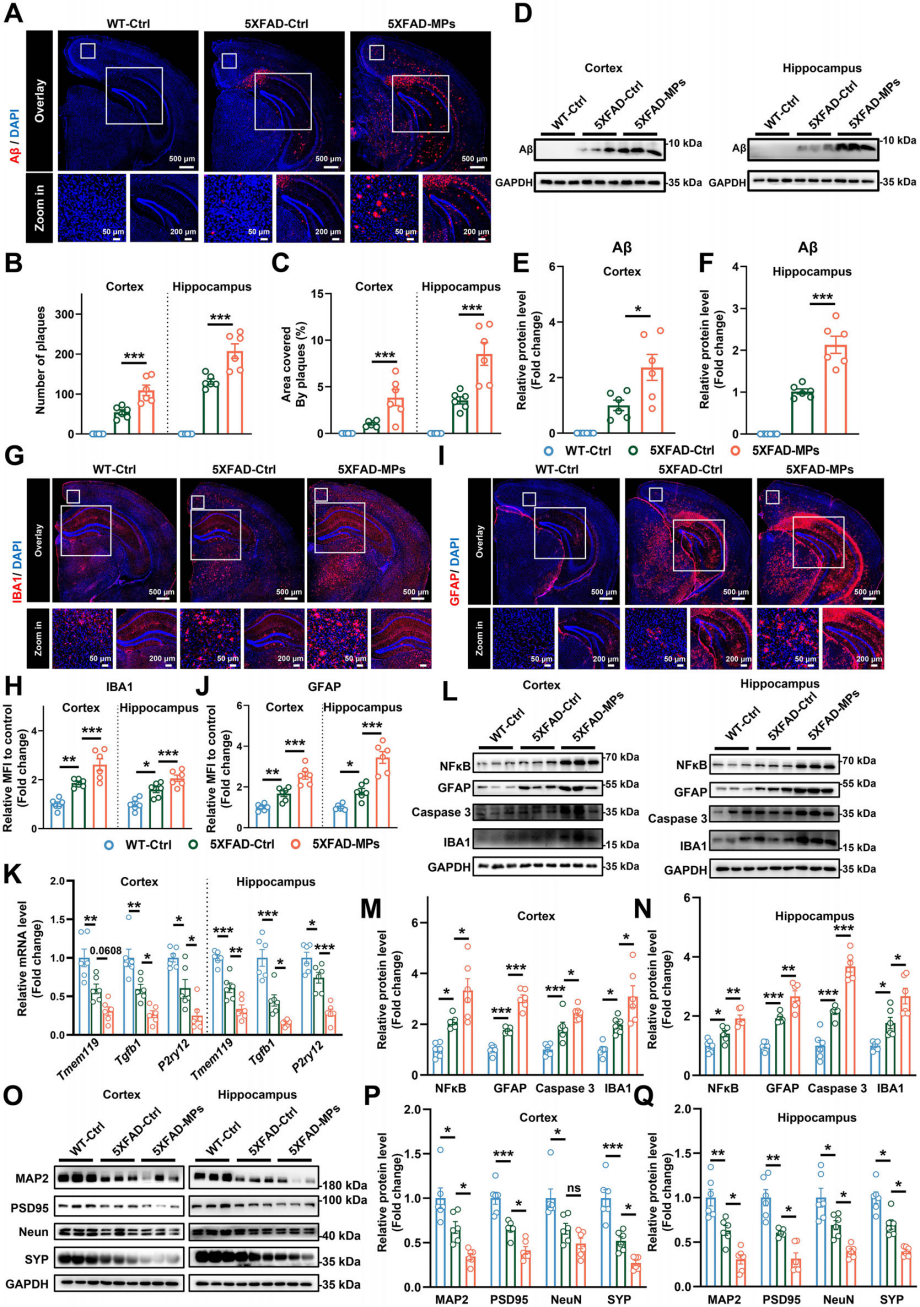
MPs ingestion exacerbates AD‐related neuropathology in 5XFAD mice. (A) Representative immunofluorescence images and (B‐C) quantification of Aβ deposition in the cortex and hippocampus of 5XFAD mice across groups. (D) Representative western blot results and (E‐F) quantification of Aβ in the cortex and hippocampus of 5XFAD mice across groups. (G) Representative immunofluorescence images and (H) mean fluorescence intensity (MFI) quantification of IBA1 in the cortex and hippocampus across groups. (I) Representative immunofluorescence images and (J) MFI quantification of GFAP in the cortex and hippocampus across groups. (K) RT‐qPCR profiling of microglial homeostasis‐ and phagocytosis‐related genes in the cortex and hippocampus among three groups. (L) Representative western blot results and (M‐N) quantification of inflammatory‐ and glial activation‐related proteins in the cortex and hippocampus of 5XFAD mice among three groups. (O) Representative western blot results and (P‐Q) quantification of neuronal /synaptic integrity markers in the cortex and hippocampus across groups. n = 6 per group. Each point denotes individual animals. Data are presented as mean ± SEM. ns, not significant; ^*^ *P* < 0.05, ^**^ *P* < 0.01, ^***^ *P* < 0.001 (Unpaired two‐tailed *t*‐test in B, C, E, F; one‐way ANOVA with Tukey's *post hoc* test in H, J, K, M, N, P, Q).

To gain insight into the effects of MPs on AD pathology, we conducted a battery of pathological experiments, including the Aβ pathology, glial activation, neuroinflammation, synaptic impairment, and disrupted autophagic flux, which are core pathological indicators in AD [2]. We found that the number of Aβ plaques and area covered by plaques (%) quantified by immunofluorescence (Figure 2A–C) and the mean optical density (MOD) quantified by immunohistochemistry (Figure S3A,B) in the cortex and hippocampus of 5XFAD mice were significantly increased after MPs treatment. In line with these, western blots showed increased Aβ level both in the cortex and hippocampus in MPs‐treated mice (Figure 2D–F). As Aβ can be cleared through autophagy, autophagic flux is markedly impaired in the brains of AD patients and mice [2]. We subsequently assessed autophagy‐related protein markers by western blot analysis. We found that autophagic flux was impaired in the brains of 5XFAD mice characterized by significantly increased levels of Sequestosome 1 (SQSTM1), lysosomal‐associated membrane protein 1 (LAMP1), and ratio of LC3‐II/LC3‐I, with significantly decreased of LMAP2A. Notably, MPs treatment further exacerbated this alteration, indicating an aggravation of autophagic dysfunction (Figure S3C–E).

As activation of microglia and astrocytes is also the pathology of AD [43], the same methods were employed to investigate the effect of MPs on neuroinflammation. We found that the mean fluorescence intensity (MFI) of ionized calcium‐binding adapter molecule 1 (IBA1, a marker of microglia) and glial fibrillary acidic protein (GFAP, a marker of astrocytes) were significantly increased in the cortex and hippocampus of 5XFAD mice compared with littermates, and MPs treatment further exacerbated these increases (Figure 2G–J). Immunohistochemistry for IBA1 and GFAP further confirmed increased microglial and astrocytic activation in 5XFAD mice compared with littermates, which was further aggravated by MPs treatment (Figure S3F–H). In line with these findings, quantitative reverse transcription PCR (RT‐qPCR) showed that the expression levels of microglial homeostasis‐ and phagocytosis‐related genes, including *Tmem119* (transmembrane protein 119), *P2ry12* (purinergic receptor P2Y12), and *Tgfb1* (transforming growth factor beta 1), were significantly reduced in 5XFAD mice compared with littermates, and were further decreased following MPs treatment (Figure 2K). Glial activation can give rise to subsequent neuroinflammatory responses [44, 45]. Western blots showed elevated protein levels of IBA1, GFAP, nuclear factor kappa B (NFκB), and full‐length caspase‐3 in the cortex and hippocampus of 5XFAD mice under MPs treatment (Figure 2L–N). RT‐qPCR analysis showed significantly increased expression levels of *Il‐6*, *Tnf‐α*, and *Il‐1β*, accompanied by significantly decreased expression levels of *Il‐4* and *Il‐10* (Figure S3I,J). Collectively, these results indicate that chronic MPs ingestion exacerbates AD‐associated glial activation and neuroinflammation.

As neuroinflammation could further contribute to synaptic impairment and neuronal loss [2, 4], we examined the levels of neuron‐ and synapse‐related proteins by western blotting, to determine whether MPs treatment aggregated synaptic impairment in 5XFAD mice. We found that the protein levels, including neuronal somatic marker (NeuN), dendritic protein microtubule‐associated protein 2 (MAP2), synaptic proteins synaptophysin (SYP), and postsynaptic density protein 95 (PSD95), were all significantly decreased in 5XFAD mice compared with littermates, and were further decreased following MPs treatment (Figure 2O–Q). To dissect the mechanism by which MPs precipitate synaptic‐protein loss in 5XFAD mice, we first asked whether neuronal viability itself is compromised. Although hematoxylin & Eosin (H&E) staining showed no obvious morphological differences among experimental groups (Figure S3K), Nissl staining revealed a marked reduction in optical density (OD) of Nissl bodies in both the cortex and hippocampus of vehicle‐treated 5XFAD animals relative to littermate controls; MP exposure further intensified this deficit (Figure S3L–N). Together, these data suggest MPs as a synergistic insult that amplifies amyloid‐driven synaptic pathology, thereby accelerating cognitive decline in the 5XFAD paradigm.

### 2.3 MPs Disrupted the Systemic Metabolome in Serum of the 5XFAD Mice and Selectively Depleted Taurine‐Dependent Cytoprotective Modules

**FIGURE 3:**
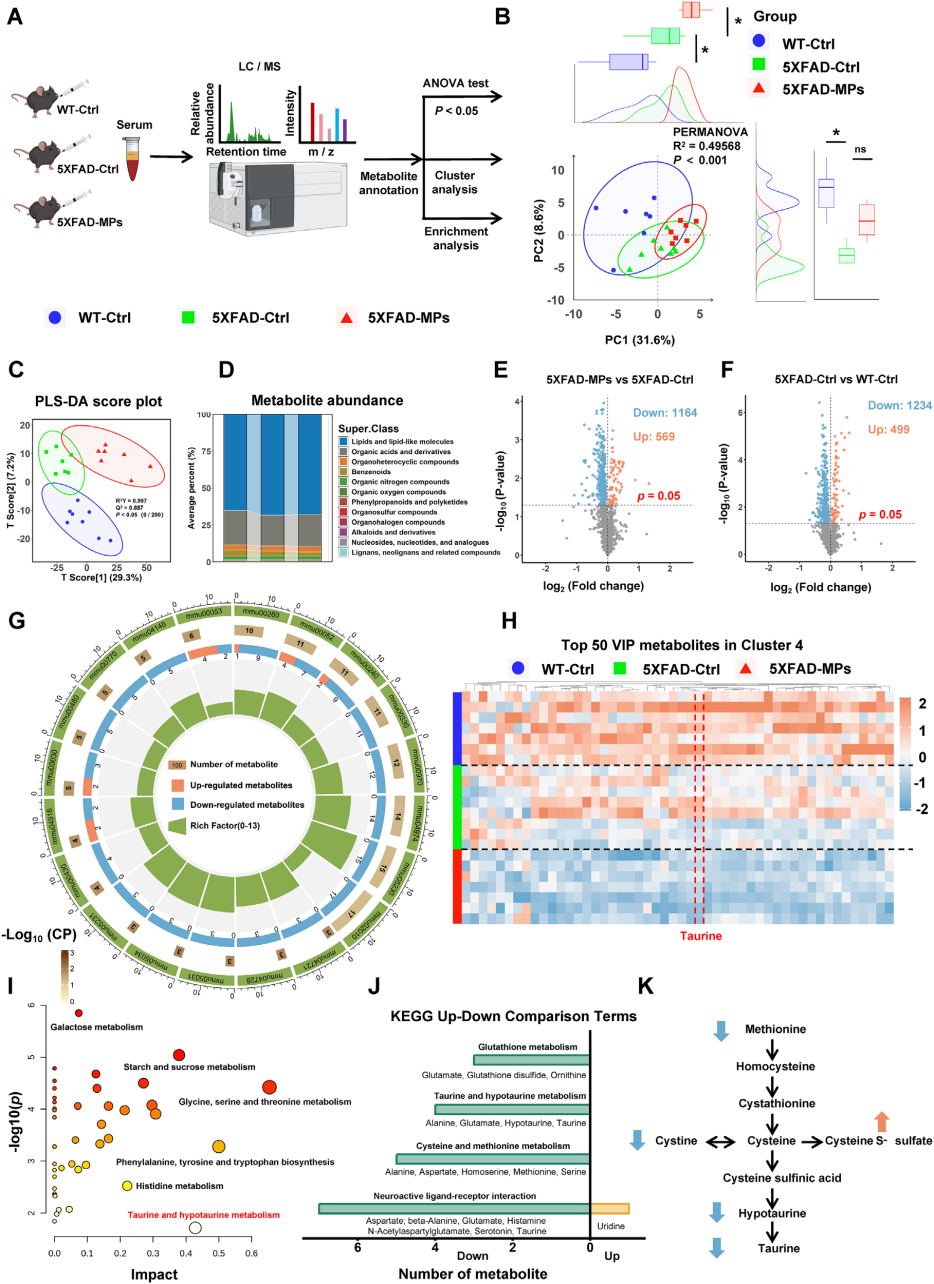
MPs exposure profoundly disrupts the serum metabolome in 5XFAD mice. (A) Schematic overview of the experimental workflow for serum metabolomics. (B) PCA score plot showing the distribution of serum metabolomic profiles among groups. Marginal boxplots and density plots indicate the distribution along PC1 and PC2. (C) PLS‐DA score plot of serum metabolomic profiles among groups. (D) Relative abundance of metabolite superclasses in serum across groups. (E, F) Volcano plots showing differential metabolites (E) between 5XFAD‐Ctrl and WT‐Ctrl, and (F) between 5XFAD‐MPs and 5XFAD‐Ctrl. Dashed lines indicate the significance threshold (p = 0.05). (G) Circular plot summarizing KEGG pathway enrichment of significantly differential metabolites, showing the number of metabolites, up‐ and down‐regulated metabolites, and enrichment significance. (H) Heatmap of the top 50 VIP metabolites in Cluster 4. Metabolite abundances were Z‐score normalized across samples. (I) Pathway impact analysis of significantly enriched metabolic pathways based on differential metabolites using MetaboAnalyst. (J) Regulation of differential metabolites in the taurine‐related metabolic pathways. Green bars indicate downregulated metabolites, and yellow bars indicate upregulated metabolites. Bar length represents the number of metabolites mapped to each pathway. (K) Schematic representation of changes in metabolite abundances within taurine‐related metabolic pathways following MPs treatment. n = 7, 8, 7 for the WT‐Ctrl, 5XFAD‐Ctrl, 5XFAD‐MPs groups, respectively. Data are presented as mean ± SEM. ns, not significant; ^*^ *P* < 0.05.

Given that metabolic dyshomeostasis precedes cognitive decline in AD [46], we therefore interrogated whether MPs acted as an environmental amplifier of this dysfunction. Untargeted liquid chromatography–mass spectrometry (LC‐MS) profiling of serum (Figure 3A) revealed discrete metabolic signatures for the WT‐control (Ctrl), 5XFAD‐Ctrl, and 5XFAD‐MPs groups. Principal‐component analysis (PCA) and Partial least‐squares discriminant analysis (PLS‐DA) revealed that the metabolomic composition was significantly different among the groups (Figure 3B,C). Classification of detected metabolites indicated that lipids, organic acids, and organoheterocyclic compounds were ranked as the top three most abundant metabolites (Figure 3D). Subsequently, we performed unpaired Student's *t*‐tests to analyze the differences in metabolite abundance between the WT‐Ctrl and 5XFAD‐Ctrl groups, as well as between the 5XFAD‐Ctrl and 5XFAD‐MPs groups (p < 0.05). Benjamini‐Hochberg FDR correction was applied, and metabolites with FDR < 0.05 were considered statistically significant. For exploratory purposes, metabolites with nominal p < 0.05 were also examined in the context of pathway‐level analyses. Compared with WT‐Ctrl, there were 499 up‐regulated metabolites and 1234 down‐regulated metabolites in the 5XFAD‐Ctrl group (Figure 3E). Compared with 5XFAD‐Ctrl, there were 569 up‐regulated metabolites and 1164 down‐regulated metabolites in the 5XFAD‐MPs group (Figure 3F). Kyoto Encyclopedia of Genes and Genomes (KEGG) enrichment analysis pinpointed three intersecting super‐pathways‐ glycine/serine/threonine, galactose, and arginine/proline metabolism [47, 48, 49] (Figure 3G, Table S1), each feeding into taurine‐centric modules like taurine/hypotaurine, cysteine/methionine, and glutathione metabolism (Table S1). Metabolites were clustered based on their relative expression patterns across the three experimental groups using an unsupervised clustering approach (Figure S4A). Among all clusters, Cluster 4 displayed expression trends that were highly consistent with AD‐related pathological changes. Cluster 4—encompassing taurine, citrulline, arginine, and lysophosphatidylcholine (LPC)—displayed expression patterns aligned with anti‐aging–related effects (Figure 3H, Table S2) [40, 50, 51]. Taurine, a well‐documented antioxidant sulfur‐containing amino acid [52] that exerts therapeutic effects in cancer [53], alleviates obesity [54], and contributes to the attenuation of aging‐related processes [55], was decreased following MPs treatment (Figure 3H, Table S2). Moreover, we found that taurine‐related metabolic pathways, particularly taurine and hypotaurine metabolism, significantly affected the metabolome by comparing the impact among all pathways calculated using MetaboAnalyst (Figure 3I,J). Consistently, multiple metabolites within taurine‐related pathways were significantly dysregulated, indicating a marked disruption of taurine metabolism (Figure 3K). In addition, clusters 1 and 2 were also analyzed, and metabolites within these clusters exhibited increased levels in 5XFAD mice, particularly following MPs treatment (Figure S4B). Collectively, MPs' chronic ingestion did not merely accentuate an existing AD metabolic signature; it reconfigured the serum metabolome into a taurine‐deficient, oxidation‐prone state that may accelerate neurovulnerable circuitry toward failure.

### 2.4 Dysregulated Serum Taurine Metabolism is Associated with Alterations in the Cecal Content Metabolome and Microbiota

Mammals acquire the bulk of their taurine exogenously [52]; hence, the cecum—the intestinal segment with the highest luminal metabolite load—becomes a gatekeeper of whole‐body taurine economy. We therefore interrogated cecal contents by untargeted LC–MS (Figure S5A). Similarly, PCA and PLS‐DA analyses revealed that the metabolomic composition in the cecal contents was significantly different among the groups (Figure S5B,C). Metabolite classification revealed that lipids, organic acids, and organoheterocyclic compounds were the three most abundant metabolite classes, consistent with the serum metabolomic profile (Figure S5D). Subsequently, we performed unpaired Student's *t*‐tests to analyze the differences in metabolite abundance between the WT‐Ctrl and 5XFAD‐Ctrl groups, as well as between the 5XFAD‐Ctrl and 5XFAD‐MPs groups. Compared with WT‐Ctrl, there were 1437 up‐regulated metabolites and 2095 down‐regulated metabolites in the 5XFAD‐Ctrl group (Figure S5E). Compared with 5XFAD‐Ctrl, there were 909 up‐regulated metabolites and 1631 down‐regulated metabolites in the 5XFAD‐MPs group (Figure S5F). To further characterize the dynamic changes of taurine in the cecal metabolome and its relative contribution to cecal metabolic dysregulation, Z‐score normalization analysis was performed based on PLS‐DA analysis. We found taurine exhibited a significant decrease in abundance and ranked among the top metabolites based on variable importance in projection (VIP) scores (displayed in descending order from left to right), indicating its prominent contribution to the observed metabolic alterations (Figure S5G). To comprehensively assess the effects of MPs treatment on the cecal metabolome, we also performed an unsupervised clustering analysis on all detected metabolites in cecal contents. All detected metabolites were classified into eight distinct clusters based on their differential expression patterns, while Clusters 3 and 4 displayed expression trends that were highly consistent with AD‐related pathological changes (Figure S6A). Notably, taurine was also included in cluster 4, and its level in cecal contents was significantly reduced in the 5XFAD‐Ctrl group compared with WT‐Ctrl, with a further decrease observed following MPs treatment (Figure S5H, Table S3). We applied the same analytical approach to clusters 1 and 2, but did not identify any metabolites associated with the taurine‐related metabolic pathway (Figure S6B). KEGG enrichment analysis results showed that MPs treatment significantly disrupted the cecal metabolome, as exemplified by perturbations in key microbial‐metabolic pathways like citrate cycle (TCA cycle), galactose and glycine/serine/threonine metabolism (Figure S5I and Table S4). We further compared taurine levels between serum and cecal contents. Quantitation confirmed a concentration gradient (cecum > serum) that collapsed concordantly after MPs exposure, suggesting that alterations in intestinal taurine availability may contribute to the observed systemic taurine deficit (Figure S5J,K).

**FIGURE 4:**
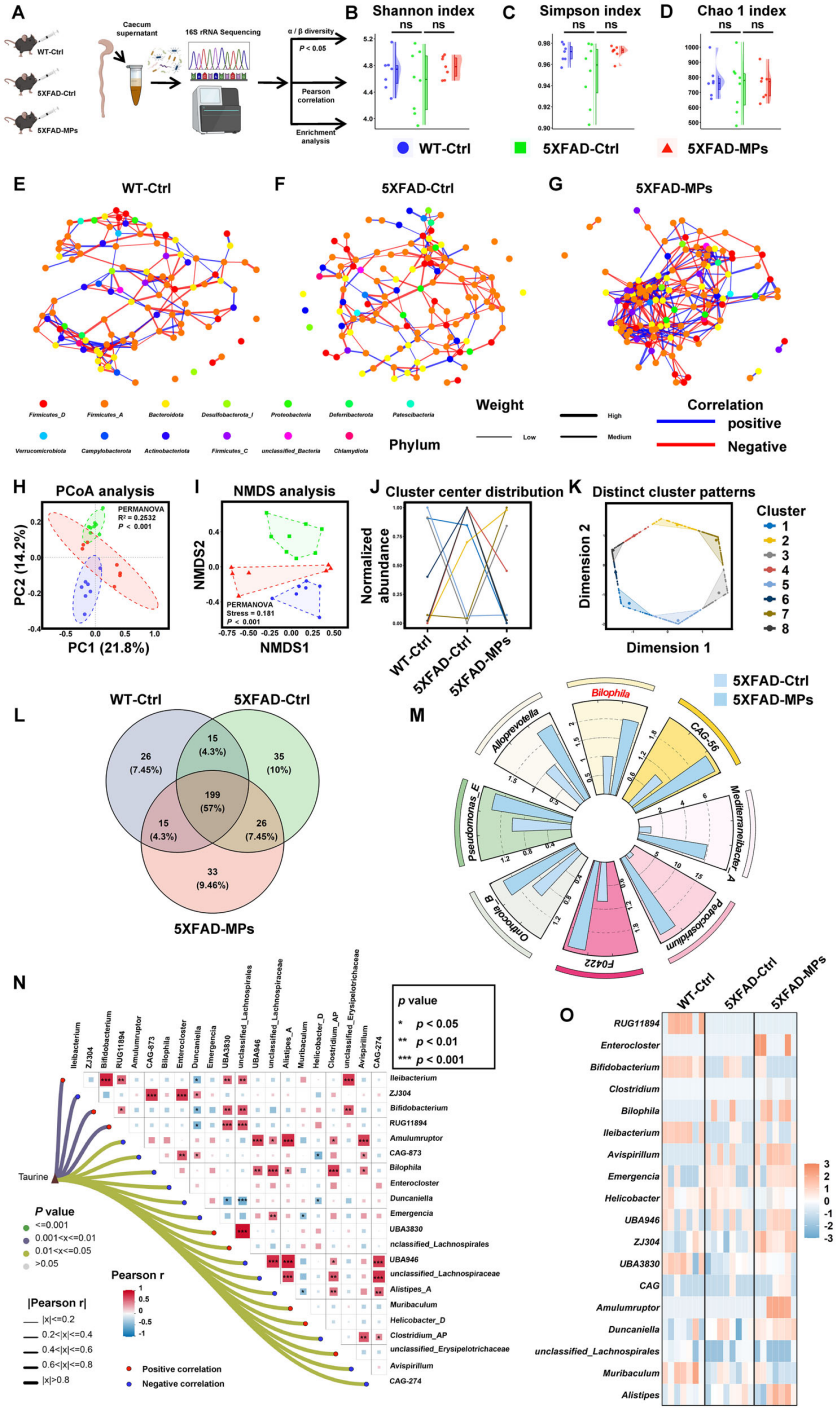
MPs exposure profoundly disrupts the microbiome of cecal contents in 5XFAD mice. (A) Experimental outline for cecal‐content collection, 16S rRNA sequencing and multi‐layered bioinformatics. (B‐D) Comparison of α diversity of microbiome as measured by (B) Shannon index, (C) Simpson index and (D) Chao 1 index among all groups (Compared by ordinary one‐way ANOVA). Box plots extend from the 25^th^ to the 75^th^ percentile with the median value shown as a black line in the middle, and whiskers denote the minima and maxima values. (E‐G) Co‐occurrence networks analysis of the microbiome in each group. Each network is presented as an independent panel. Within each network, nodes represent bacterial classes (colored by phylum), and edges denote significant correlations (with color indicating positive or negative interactions). (H) β diversity, as measured by PCoA of Bray‐Curtis distances. Ellipses indicate 95% confidence intervals per group. *P* and R^2^ were calculated by PERMANOVA analysis. (I) β diversity, as measured by NMDS of Bray‐Curtis distances of the microbiome of cecal contents among all groups. *P* and Stress were calculated by PERMANOVA analysis. (J‐K) K‐means clustering of the gut microbiota was performed based on abundance profiles across the three groups. All the taxa were partitioned into eight distinct and complementary clusters. (L) Venn diagram depicting the overlap of genera among the three groups. The diagram illustrates the number of taxa that are shared by all groups or unique to each group. (M) Display of the genera that are both exclusive to the 5XFAD‐Ctrl and 5XFAD‐MPs groups and assigned to Cluster 2. (N) Correlation analysis between taurine and the genus taxa of the microbiota of the cecal contents in 5XFAD mice. *P* and r were calculated by Pearson correlation analysis, while the correlation among the microbiota was analyzed with color indicating positive or negative interactions. (O) Heatmap showing Z‐score‐normalized abundances of taurine‐associated bacterial genera, ranked by linear discriminant analysis (LDA) effect size. Each point represented a mouse. n = 7, 8, 7 for the WT‐Ctrl, 5XFAD‐Ctrl, 5XFAD‐MPs groups, respectively.

The cecal microbiota plays a crucial and dynamic role in host gut metabolism [56]. To further investigate the potential mechanism by which MPs exacerbated AD pathology, we conducted 16S rRNA sequencing of the cecal contents from WT‐Ctrl, 5XFAD‐Ctrl, and 5XFAD‐MPs mice (Figure 4A). alpha diversity metrics—including the Shannon, Simpson, and Chao1 indices—showed no significant differences among the three groups (Figure 4B–D). Similarly, ASV counts, ACE index, and rarefaction curves confirmed comparable species richness and evenness, indicating that MPs did not overtly alter microbial abundance or uniformity in 5XFAD mice (Figure S7A–C). Given that the gut microbiota constitutes a complex micro‐ecosystem characterized by intricate interspecies interactions, we next constructed bacterial co‐occurrence networks for each group. These analyses revealed markedly distinct microbial interaction topologies: MPs‐treated 5XFAD mice exhibited a marked increase in network complexity, reflected by a higher number of strong pairwise correlations (both positive and negative) among bacterial taxa (Figure 4E–G and S7D–F). This suggested that MP exposure substantially rewired microbial interrelationships within the 5XFAD gut environment. Beta‐diversity analyses via principal coordinate analysis (PCoA) and non‐metric multidimensional scaling (NMDS) further demonstrated that MPs treatment drove a significant shift in overall microbiota composition in 5XFAD mice (Figure 4H,I). Taxonomic profiling uncovered group‐specific microbial signatures, with conspicuous divergence in the relative abundances of shared phyla, classes, orders, and families (Figure S7G–J). Circos plots corroborated these findings, illustrating both the differential abundance of individual genera across groups and their altered proportional representation within each community (Figure S7K). Collectively, these data indicate that chronic MPs ingestion profoundly perturbed the structure and composition of the cecal microbiota in 5XFAD mice, potentially contributing to the exacerbation of AD‐associated pathophysiology.

To dissect group‐specific reconfiguration of the cecal microbiota, we applied K‐means clustering to the full microbial‐feature matrix. Eight non‐redundant, co‐abundance clusters were resolved that collectively recapitulated the inter‐group partitioning observed in the ordination analyses (Figure 4J,K). Among these, cluster 2 displayed a trajectory that precisely tracked the exacerbated AD phenotype seen in MPs‐treated 5XFAD mice (Figure S7L). Venn mapping next identified 26 genera shared exclusively between the 5XFAD‐Ctrl and MPs‐treated 5XFAD groups, while eight of these were classified into Cluster 2 and exhibited a consistent step‐wise abundance shift that paralleled disease progression (Figure 4L,M). Most prominent was *Bilophila*, a taurine‐respiring, hydrogen‐sulfide‐producing genus previously implicated in barrier disruption and mucosal inflammation [57, 58, 59]. Its relative abundance rose sharply after MPs treatment (Figure 4M), suggesting a mechanistic link between this pathobiont and MPs‐aggravated AD pathology. Linear discriminant analysis (LDA) effect size (LEfSe) further resolved disease‐ and treatment‐specific biomarkers. Health‐associated taxa— *Akkermansia*, *Muribaculum*, and *Ileibacterium* [60, 61, 62, 63, 64], were enriched in the WT‐Ctrl group, whereas 5XFAD‐MPs mice were selectively colonized by *Amulumruptor* and *Enterocloster* (Figure S8A–D).

To dissect the metabolite–microbiota nexus in the cecal contents, we performed pairwise Pearson correlations between taurine concentration and the abundance of individual genera, and found that 21 genera were significantly associated with taurine abundance (Figure 4N). Eight taxa, including health‐associated *Ileibacterium*, *Bifidobacterium*, and *Muribaculum*, tracked positively with taurine, whereas thirteen genera displayed inverse relationships. Chief among the latter were *Enterocloster*, *Emergencia*, and the sulfite‐reducing pathobiont *Bilophila*, corroborating the proposed role of *Bilophila* in taurine respiration and H_2_S generation. To visualize the relative contributions of these taurine‐responsive taxa, Z‐score‐normalized abundances were rank‐ordered and displayed as a heatmap (Figure 4O). *RUG11894*, *Enterocloster*, *Bifidobacterium*, *Clostridium*, and *Bilophila* emerged as the dominant drivers, with *Bilophila* occupying the uppermost tier—an observation that strengthens its functional link to taurine turnover. Moderate correlations were also detected for *Akkermansia* and ancillary taxa (Figure S8E–H). Finally, KEGG and MetaCyc pathway enrichment analyses were performed to characterize the functional profiles of the microbiota. Notably, several altered pathways were closely related to AD‐associated metabolic processes, including neurodegenerative disease pathways, as well as key metabolic pathways such as amino acid metabolism, lipid metabolism, energy metabolism, carbohydrate metabolism, and metabolism of cofactors and vitamins (Figure S9A,B). These pathways are known to be involved in neuronal metabolism, energy homeostasis, and neurodegeneration. Collectively, these data establish taurine as a pivotal metabolite whose cecal concentration is tightly coupled to specific microbial guilds, providing a plausible mechanistic bridge between MPs‐induced dysbiosis and exacerbated Alzheimer's pathology.

### 2.5 MPs‐Driven Exacerbation of AD Pathology is Dependent on the Gut Microbiota

To determine whether the gut is an initial target of orally administered MPs, 3‐month‐old 5XFAD mice received rhodamine‐B‐labeled MPs daily for 14 days. Ex vivo imaging revealed strong fluorescence throughout the small and large intestine (Figure S10A,B), confirming that MPs accumulate along the gastrointestinal tract. Previous studies showed that MPs treatment disrupted intestinal homeostasis and altered the local intestinal microenvironment [14, 65]. We next assessed the effects of MPs treatment on the duodenum and colon of 5XFAD mice using H&E staining and tissue immunofluorescence analyses. H&E staining showed no overt differences in mucosa layer thickness, villus length, and muscularis externa thickness between WT and 5XFAD mice, whereas MPs treatment markedly impaired these morphological features, indicating structural damage to the intestine (Figure S10C–F). Consistent with this, immunofluorescence results suggested that the MFI of Zonula Occludens‐1 (ZO‐1), a core component of tight junction structure, was significantly decreased both in the duodenum and colon (Figure S10G–J). Collectively, these results suggested that MPs could accumulate in the intestine and cause dysfunction of the intestinal barrier.

**FIGURE 5:**
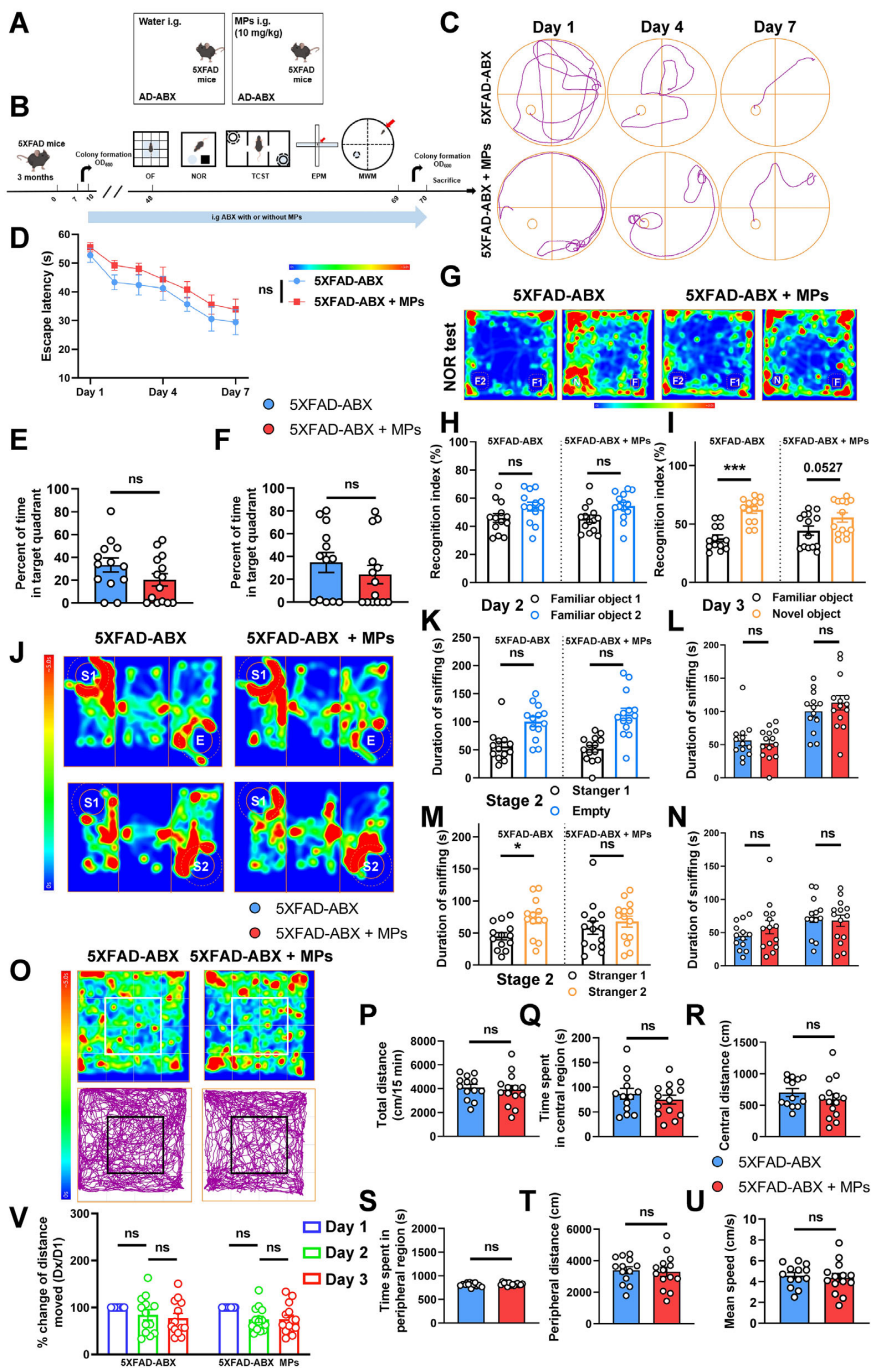
Antibiotic‐mediated gut microbiota depletion prevents MPs‐induced aggravation of AD‐related behavioral deficits in 5XFAD mice. (A‐B) Experimental scheme illustrating co‐administration of ABX and MPs to deplete the gut microbiota in 5XFAD mice, followed by behavioral assessments and pathological experiments. (C) Representative swim paths recorded during acquisition. (D) MWM navigation training showed escape latency across consecutive training days. (E‐F) Percentage of time in the target quadrant in the probe tests at (E) 4 h and (F) 72 h after the final training session. (G) Representative heatmap from the NOR task. (H‐I) Recognition index (%) of mice in (H) the day 2 between two similar objects and (I) the day 3 between the novel object and the familiar object. (J) Representative heatmaps from the TCST task. (K) Duration of sniffing (s) between S1 and Empty in each group. (L) Duration of sniffing (s) to Empty or S1 between the two groups. (M) Duration of sniffing (s) between S1 and S2 in each group. (N) Duration of sniffing (s) to S1 or S2 between the two groups. (O) Representative heatmaps and tracked paths from the OF test. (P‐U) Comparison of (P) total distance (cm/15 min), (Q) time spent in the central region, (R) central distance (cm), (S) time spent in the peripheral region (s), (T) peripheral distance (cm) and (U) mean speed (cm/s) between two groups. (V) Locomotor habituation index: percentage reduction in total OF distance on days 2 and 3 relative to day 1. The group sizes were n = 13 for the 5XFAD‐ABX group (7 males and 6 females) and n = 14 for the 5XFAD‐ABX+MPs group (7 males and 7 females). Each point denotes individual animals. Data are presented as mean ± SEM. ns, not significant; ^*^ *P* < 0.05, ^**^ *P* < 0.01, ^***^ *P* < 0.001 (Unpaired two‐tailed *t*‐test in E, F, H, I, K‐N, P‐U; one‐way ANOVA with Tukey’s *post hoc* test in V; Two‐way ANOVA with Tukey's *post hoc* test in D).

We next asked whether the MPs‐induced exacerbation of pathology is dependent on the gut microbiota. 5XFAD mice were subjected to microbiota ablation with a broad‐spectrum antibiotic (ABX) cocktail before concomitant MPs or vehicle administration, followed by subsequent behavioral and pathological assessments (Figure 5A and 5B). To confirm effective depletion of the gut microbiota, 3‐month‐old 5XFAD mice were administered ABX by oral gavage for 7 consecutive days to remove gut microbiota, followed by a colony formation assay (CFA). We found that ABX administration virtually eliminated culturable fecal bacteria (Figure S11). Behavioral testing revealed that MPs treatment no longer impaired learning or memory in ABX‐treated animals. During MWM acquisition, escape latencies were indistinguishable between 5XFAD‐ABX and 5XFAD‐ABX+MPs mice (Figure 5C,D). Probe‐trial indices—time in target quadrant, platform crossings, latency to first crossing—were likewise comparable at 4 h and 72 h (Figure 5E,F and Figure S12A–J). NOR, TCST, OF, and Elevated plus maze (EPM) metrics also showed no MPs‐specific deficits (Figure 5G–V; Figure S12K–Q). Collectively, these results suggested that the exacerbation of AD‐related behavioral deficits by MPs is dependent on gut microbiota.

To gain insight into the role of gut microbiota in mediating the effects of MPs on AD pathology, we next performed pathological analyses. Aβ plaque number and area in the cortex and hippocampus were identical in 5XFAD‐ABX and 5XFAD‐ABX+MPs mice (Figure S13A–C), and western blotting revealed no significant MPs‐induced rise in Aβ or alteration of autophagy markers (LAMP1, LAMP2A, SQSTM1, LC3‐II/LC3‐I) (Figure S13D,E). Similarly, microgliosis (IBA1) and astrogliosis (GFAP) were unchanged by MPs after ABX (Figure S13F–I), and expression of homeostatic (*Tmem119*, *P2ry12*, *Tgfb1*) and inflammatory transcripts/proteins remained stable (Figures S13J,K and S14A–C). Synaptic markers (MAP2, PSD‐95, NeuN, SYP), H&E‐based quantification of neuronal density and Nissl‐bodyOD were also unaffected (Figures S13L,M and S14D–F), indicating preserved neuronal integrity. In addition, we assessed the effects of MPs treatment on the duodenum and colon of 5XFAD mice following ABX treatment. ABX treatment failed to alleviate the MPs‐aggravated morphological impairment of the duodenal barrier in 5XFAD mice, as evidenced by persistent reductions in mucosa layer thickness, villus length, and muscularis externa thickness (Figure S14G,H). Remarkably, ABX did not prevent MPs‐induced intestinal injury: ZO‐1 level remained low in the duodenum and colon of 5XFAD‐ABX+MPs mice (Figure S14I–L), confirming that barrier damage is a direct physicochemical effect of MPs, independent of bacteria.

**FIGURE 6:**
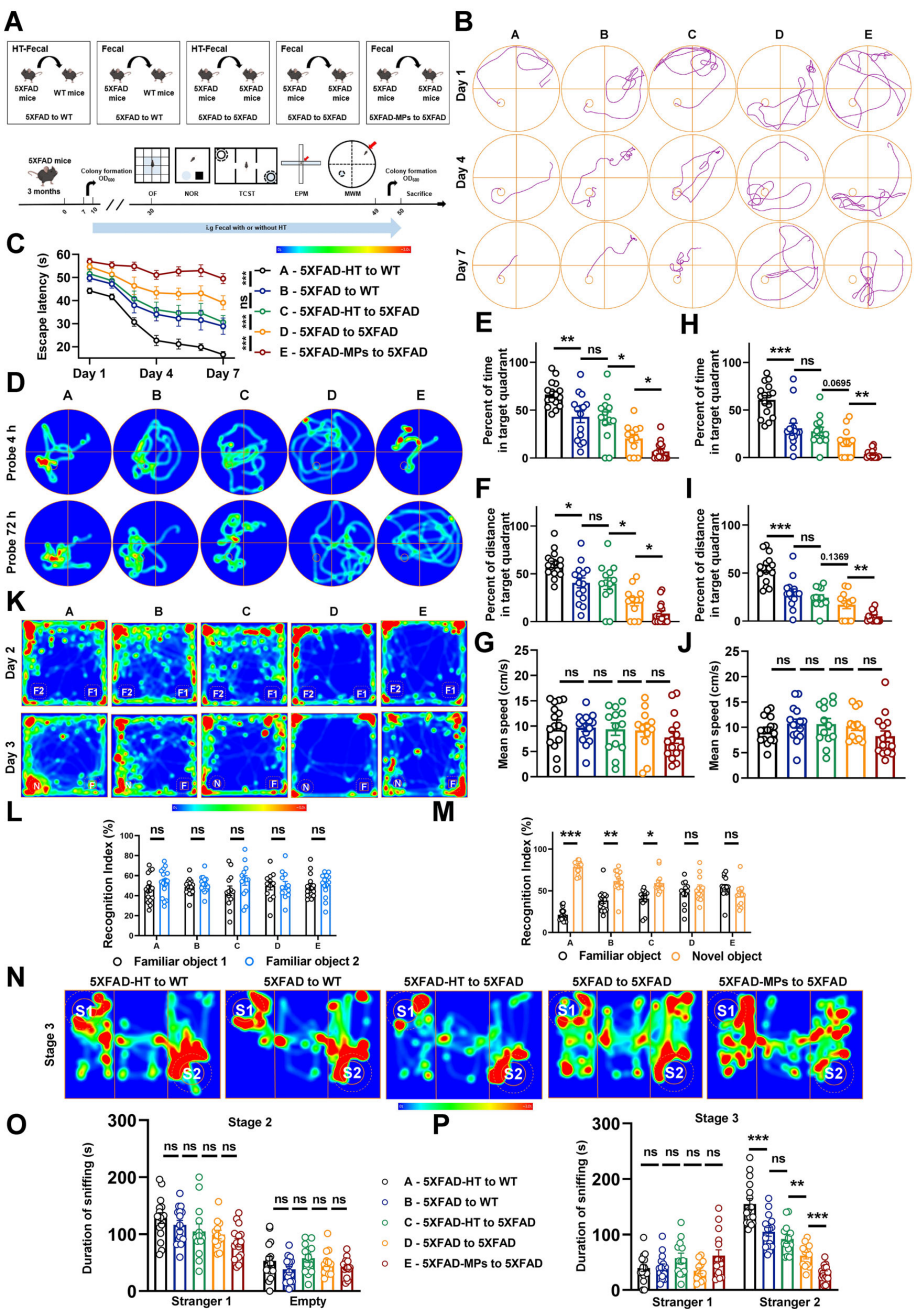
FMT transfers MPs‐associated cognitive and social behavioral deficits to recipient mice. (A) Schematic overview of the experimental design. ABX‐treated recipient WT or 5XFAD mice received fecal microbiota (with or without heat‐killed treated [HT]) from distinct donor groups: 5XFAD control mice (5XFAD‐Ctrl) or 5XFAD mice exposed to microplastics (5XFAD‐MPs), followed by comprehensive behavioral assessments. (B) Representative track plots from the MWM test for each group. (C) MWM acquisition training: escape latency across consecutive training days. (D) Representative heatmaps and track plots during the 4 h and 72 h probe trials of the MWM test for each group. (E‐G) Quantitative analysis of the 4 h probe test: (E) percentage of time in the target quadrant (%), (F) percent of distance in target quadrant (%), and (G) mean speed (cm/s). (H‐J) Quantitative analysis of the 72 h probe test: (H) percentage of time in the target quadrant (%), (I) percent of distance in the target quadrant (%), and (J) mean speed (cm/s). (K) Representative heatmaps from the NOR test for each group. (L‐M) Recognition index (%) of mice in (L) on day 2 between two similar objects and (M) on day 3 between the novel object and the familiar object. (N) Representative heatmaps of Stage 3 in TCST of mice in each group. (O) Comparison of duration of sniffing (s) toward Empty or S1 among the three groups. (P) Comparison of duration of sniffing (s) to S1 or S2 among the three groups. The group sizes were n = 16 (8 males and 8 females) for Group A, n = 15 (8 males and 7 females) for Group B, n = 13 (7 males and 6 females) for Group C, n = 12 (6 males and 6 females) for Group D, and n = 15 (8 males and 7 females) for Group E. Each data point represents an individual mouse. Data are presented as mean ± SEM. ns, not significant; ^*^ *P* < 0.05, ^**^ *P* < 0.01, ^***^ *P* < 0.001 (unpaired Student's *t*‐test in L and M; one‐way ANOVA with Tukey's *post hoc* test E‐J, O and P; Two‐way ANOVA with Tukey's *post hoc* test in C).

To determine whether the neurocognitive deterioration driven by MPs is microbiota‐mediated, we performed FMT after microbiota depletion by ABX treatment (Figure 6A). We found that ABX administration efficiently depleted the gut microbiota in 5XFAD mice, as evidenced by a marked suppression of bacterial colony formation on LB agar plates and a significant reduction in the OD600 values of fecal homogenates (Figure S15A,B). Following FMT and subsequent behavioral assessments, the same validation assays were repeated to confirm the successful engraftment of the transplanted microbiota. FMT‐recipient groups consistently showed increased microbial abundance, supporting successful microbiota engraftment (Figure S15C,D). Behavioral analyses revealed that spatial learning deficits are transmissible. In the MWM, microbiota‐ablated WT recipients receiving 5XFAD‐derived microbiota displayed prolonged escape latencies; this impairment was further amplified when donors had been exposed to MPs (Figure 6B,C). Probe‐trial metrics (percentage of time in target quadrant, percentage of distance in target quadrant, platform crossings, latency to first crossing) were comparably affected at 4 h and 72 h (Figure 6D–J, Figure S16A–D), although no significant differences in mean swimming speed were observed among all groups. NOR test showed no significant differences in exploration time between the two identical objects among all the groups during the familiarization phase (Figure 6K,L). However, WT recipient mice receiving fecal microbiota from 5XFAD donors exhibited a significant reduction in the proportion of time spent exploring the novel object compared with microbiota‐ablated WT controls, and this deficit was further exacerbated in recipients transplanted with microbiota from MPs‐treated 5XFAD donors (Figure 6K,M). TCST showed all FMT recipient groups spent significantly more time sniffing the S1 mouse than the empty chamber, indicating preserved sociability (Figure 6O, Figure S16E,F). In the social novelty phase, WT recipient mice transplanted with fecal microbiota from 5XFAD donors exhibited a significantly reduced sniffing time toward the novel mouse compared with microbiota ablated WT recipients, while fecal microbiota transplantation from untreated 5XFAD donors impaired social novelty recognition relative to microbiota ablated 5XFAD controls, and this deficit was further exacerbated following transplantation with microbiota from MPs‐treated donors (Figure 6N,P, Figure S16G). OF and EPM assays likewise demonstrated exacerbated anxiety and impaired habituation exclusively in recipients of 5XFAD‐MPs microbiota (Figure S16H–V).

Since microbiota transplantation could cause AD‐related behavioral deficits, we further investigated the effect of microbiota transplanted from donors to recipients on AD‐related pathology. Immunofluorescence and western blotting revealed increased Aβ deposition and impaired autophagy in the cortex and hippocampus of recipients (Figure S17A–E). Immunofluorescence and RT‐qPCR showed that microgliosis and astrogliosis were elevated, homeostatic microglial genes down‐regulated, and pro‐inflammatory cytokines were up‐regulated (Figures S17F–I and S18A–E). Western blotting, H&E, and Nissl staining showed that synaptic proteins (PSD‐95, SYP, MAP2, NeuN) and Nissl‐body optical density were reduced, although neuronal density was not significantly changed (Figure S18F–G and S19A–D). Notably, neither 5XFAD nor 5XFAD‐MPs microbiota altered intestinal tight junction proteins (ZO‐1 and Occludin) or villus morphology in recipients (Figure S20A–K), indicating that barrier injury is a direct effect of MPs, whereas neurodegeneration is microbiota‐driven. Collectively, these data suggest that MPs shape a disease‐aggravating gut community whose transfer is sufficient to recapitulate the AD‐like cognitive and neuropathological deficits.

### 2.6 Taurine Supplementation Alleviated AD Pathology and Cognitive Decline Exacerbated by MPs

To determine whether restoring taurine level could offset the microbiota‐dependent deterioration caused by MPs, 3‐month‐old 5XFAD mice received daily oral taurine (500 mg kg^−^^1^) for 70 days concomitant with continued MP exposure (Figure S21A). In the MWM task, MPs‐treated 5XFAD mice displayed prolonged escape latencies that were fully normalized by taurine (Figure S21B,C). Probe‐trial metrics—latency to first platform crossing, time and distance in the target quadrant, and platform crossings—were likewise restored at both 4 and 72 h, without alteration of swim speed (Figures S21D–J and S22A–D). Consistently, in the NOR test, no significant differences were observed among groups during the familiarization phase, indicating comparable exploratory activity toward identical objects (Figure S21K,L). In contrast, 5XFAD mice treated with MPs exhibited a significant reduction in the proportion of time spent exploring the novel object, while taurine supplementation significantly attenuated this reduction, indicating an improvement in recognition memory deficits induced by MPs (Figure S21K,M). In addition, mice from all groups spent significantly more time sniffing the S1 mouse than the empty chamber during the sociability phase (Figures S21O and S22E). In the social novelty phase, MPs‐treated 5XFAD mice exhibited a significantly reduced sniffing time toward the novel mouse. Notably, in the context of MPs treatment, taurine supplementation significantly ameliorated the impairment in social novelty preference (Figures S21N,P and S22G). Furthermore, OF and EPM tests were performed in mice receiving taurine supplementation. We found MPs‐treated 5XFAD mice exhibited increased anxiety, as indicated by reduced center time and distance in the OF test, whereas taurine supplementation significantly increased these measures, suggesting that taurine treatment alleviated MPs‐induced anxiety (Figure S22H–N). Notably, in the context of MPs treatment, 5XFAD mice administered with taurine exhibited a significant decrease in total distance compared with Day 1 during the three days, indicating that taurine supplementation improved the adaptability of MPs‐treated 5XFAD mice to a novel environment (Figure S22O). Consistent with this, based on comparable locomotor speed, total distance, and entries into the open arms, 5XFAD mice treated with both MPs and taurine exhibit more time spent and entries of open arm compared with those treated only with MPs, indicating adaptation to the EPM (Figure S22P–V). Thus, taurine supplementation ameliorates MP‐aggravated cognitive and affective deficits.

**FIGURE 7:**
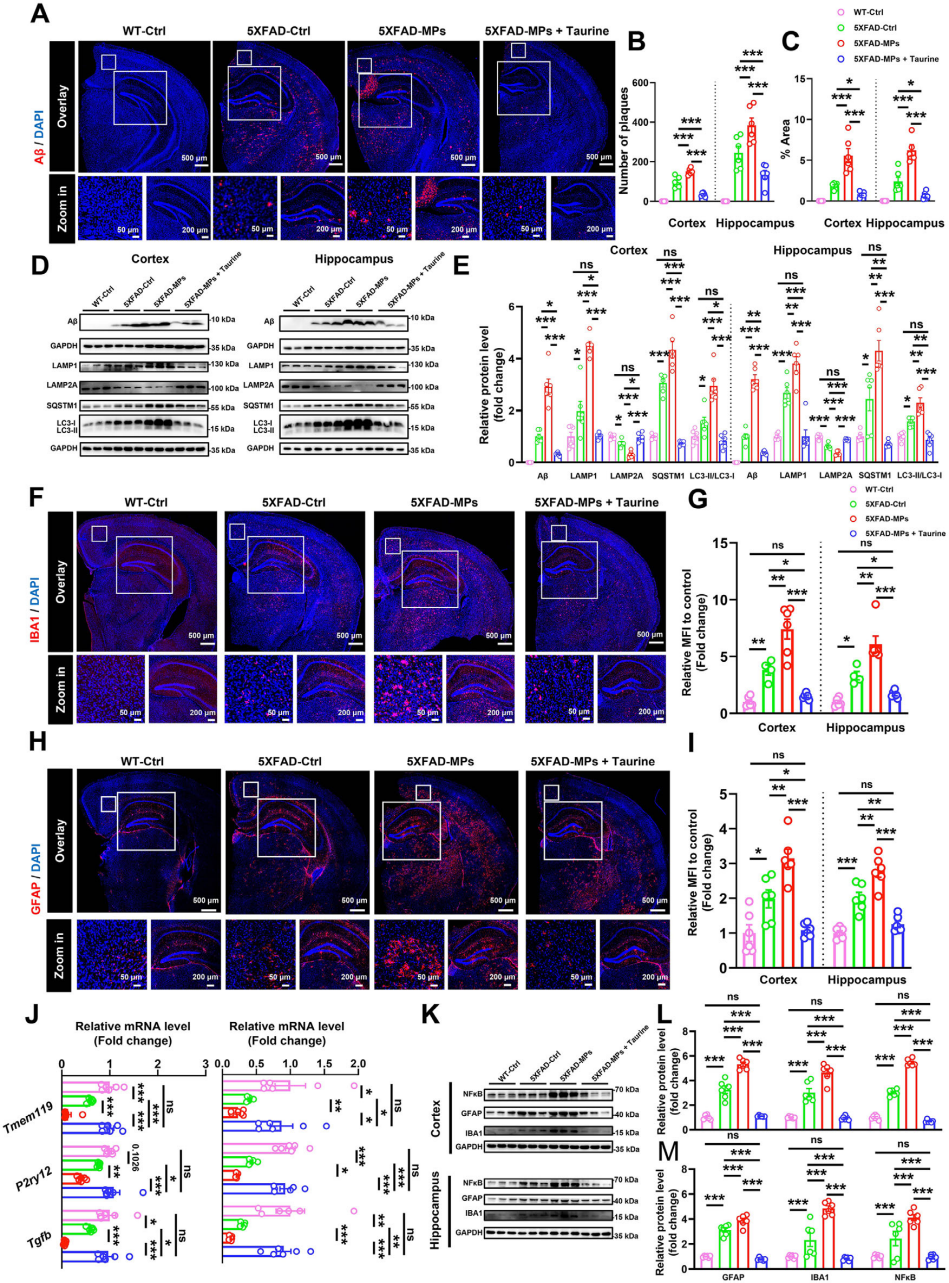
Taurine supplementation alleviates MPs‐exacerbated AD‐related pathology in 5XFAD mice. (A) Representative cortical and hippocampal immunofluorescence images showing Aβ deposition (red) with DAPI counterstaining (blue) in each group. (B‐C) Quantification of Aβ plaque number (B) and plaque area (C) in the cortex and hippocampus. (D) Representative western blots of Aβ and autophagy‐related proteins (LAMP1, LAMP2A, SQSTM1, and LC3‐II / LC3‐I) in cortical and hippocampal tissues. (E) Quantification of the level of Aβ and autophagy‐related proteins in D. (F) Representative cortical and hippocampal immunofluorescence images and (G) quantification of the MFI of IBA1 among all groups. (H) Representative cortical and hippocampal immunofluorescence images and (I) quantitative analysis of the MFI of GFAP among all groups. (J) RT‐qPCR profiling of microglial homeostasis/phagocytosis‐related genes in the cortex and hippocampus among all groups. (K‐M) Representative western blots (K) and quantification (L‐M) of the level of inflammatory‐ and glial activation‐related proteins in cortex and hippocampus among all groups. Each point denotes individual animals. n = 6 biologically independent mice per group. Data are presented as mean ± SEM. ns, not significant; ^*^ *P* < 0.05, ^**^ *P* < 0.01, ^***^ *P* < 0.001 (one‐way ANOVA with Tukey's *post hoc* test in B and C, E and G, I and J, L and M).

We next assessed the effects of taurine on improving AD‐related pathology in MPs‐treated 5XFAD mice. Immunofluorescence and western blotting revealed that taurine reduced cortical and hippocampal Aβ pathology in MPs‐treated 5XFAD mice (Figure 7A‐E). Notably, taurine administration significantly increased autophagic flux in the 5XFAD mice treated with MPs (Figure 7D,E). Microgliosis and astrogliosis (IBA1 and GFAP mean fluorescence intensity) were markedly attenuated (Figure 7F–I), coinciding with up‐regulation of homeostatic/phagocytic genes (Figure 7J). In addition, western blotting revealed that taurine treatment significantly reduced the protein levels associated with glial activation and neuroinflammation both in the cortex and hippocampus of MPs‐treated 5XFAD mice (Figure 7K–M). In addition, RT‐qPCR analysis exhibited a similar trend, showing that taurine supplementation significantly downregulated pro‐inflammatory cytokine expression while upregulating anti‐inflammatory cytokine expression (Figure S23A–D). Furthermore, synaptic proteins (PSD‐95, SYP, MAP2, NeuN) and OD of Nissl‐bodies reduced by MPs were restored by taurine (Figure S23E–J), although H&E staining did not reveal significant neurodegenerative changes (Figure S23K). Collectively, taurine normalized Aβ burden, autophagic flux, gliosis, and synaptic integrity in MP‐exposed 5XFAD mice.

As previous studies showed that taurine treatment mitigated colitis‐induced disruption of the intestinal barrier [66], we asked whether taurine also protected the gut from MP injury. H&E staining and ZO‐1 immunostaining demonstrated that taurine preserved villus architecture and tight‐junction integrity in the duodenum and colon of MPs‐treated 5XFAD mice (Figure S24), indicating that taurine concurrently fortified the intestinal barrier. In summary, taurine supplementation simultaneously rescued gut barrier function, re‐equilibrated the microbiota–brain axis, and prevented MPs‐accelerated Alzheimer‐like pathology, identifying taurine as a readily translatable intervention against environment‐linked neurodegeneration.

### 2.7 Taurine Deficiency in AD

To determine whether the taurine depletion observed in 5XFAD mice is mirrored in humans, we analyzed taurine concentrations across individuals with different cognitive statuses in the Alzheimer's Disease Neuroimaging Initiative (ADNI) cohort. Plasma taurine levels from 411 participants, including 226 cognitively normal (CN) individuals and 185 patients with Alzheimer's disease (AD), were included for analysis and compared between the two groups, followed by a correlation analysis with clinical cognitive assessment scores (Figure S25A). Of note, taurine levels in the plasma were significantly lower in the AD group compared with the CN group (Figure S25B). The Alzheimer's Disease Assessment Scale–Cognitive Subscale (ADAS‐Cog) and the Clinical Dementia Rating (CDR) scale are widely used clinical instruments for comprehensive assessment of Alzheimer's disease and related cognitive impairment states, with higher scores indicating greater disease severity. As a result, plasma taurine levels exhibited significant negative correlations with both the ADAS‐Cog and CDR scores, indicating lower taurine levels in individuals with more severe cognitive impairment (Figure S25C,D). In addition, plasma taurine levels were inversely correlated with the annual rate of change in CDR scores (%), suggesting an association between lower taurine levels and faster cognitive decline (Figure S25E). Furthermore, the Mini‐Mental State Examination (MMSE) is a widely used clinical instrument for assessing cognitive impairment and related clinical features, with higher scores indicating better cognitive status. Consistently, plasma taurine levels were significantly positively correlated with MMSE scores (Figure S25F). Collectively, these translational data validate our pre‐clinical findings and identify plasma taurine as a readily measurable biomarker linked to AD severity and rate of decline.

## 3 Discussion

Accumulating evidence indicates that AD is increasingly recognized as a multifactorial disorder influenced by chronic environmental stressors in addition to genetic risks [67, 68]. In this study, we provide evidence that environmentally relevant exposure to MPs acts as such a stressor by disrupting the gut microbiota‐taurine‐brain axis. Using an integrated framework combining behavioral assessments, neuropathological analyses, multi‐omics profiling, and microbiota manipulation, we show that chronic MPs treatment accelerated cognitive decline and AD‐related pathology in 5XFAD mice in a microbiota‐dependent manner. Taurine deficiency tracked disease severity not only in mice but also in an independent human AD cohort, establishing a trans‐species metabolite signature that is both necessary and sufficient for MPs‐aggravated neurodegeneration. Moreover, microbiota depletion and transplantation experiments, together with taurine supplementation, support a causal and reversible contribution of microbiota‐driven taurine dysregulation to MPs‐exacerbated neurodegenerative phenotypes. Our findings identify the environmental microbiome as a reversible driver of dementia risk and position taurine supplementation as a plausible prophylaxis against pollution‐linked AD acceleration.

AD and related neurodegenerative disorders are no longer regarded as cell‐autonomous and age‐dependent proteinopathies. It is increasingly viewed as a systemic disorder that emerges from the lifelong conversation between intrinsic aging clocks and extrinsic modulators of the exposome [2, 68, 69, 70, 71]. Among these modulators, MPs have become an inescapable component of modern life: minute, persistent, and continuously ingested at low, sub‐toxic doses that rarely trigger acute symptoms yet never fully clear [14, 72, 73]. Unlike acute neurotoxic insults, MPs are unlikely to elicit overt toxicity; instead, they may progressively modulate host physiological states over time, intersecting with age‐related vulnerabilities of the nervous system. Such chronic modulation is particularly relevant to neurodegenerative diseases [74], in which cumulative disruptions of metabolic balance, immune surveillance, and brain‐periphery communication play critical roles in disease initiation and progression [75, 76]. While emerging evidence indicates that MPs exposure may induce cognitive deficits in wild‐type animals through direct neurotoxicity and systemic inflammation [30, 31, 32], the present study demonstrates that MPs substantially exacerbate AD‐specific pathological hallmarks—including Aβ accumulation, autophagic dysfunction, and microglial activation—in a genetically susceptible background. This suggests that MPs may act as an environmental modifier that accelerates neurodegenerative progression in vulnerable populations. Here, we provide causal evidence that this barely perceptible exposure is sufficient to shift the trajectory of Alzheimer's pathology. Using the 5XFAD mouse model, we demonstrate that sustained MPs treatment robustly accelerates multiple core features of AD‐related pathology. MPs exposure aggravated learning and memory deficits across complementary behavioral paradigms and amplified major hallmarks of AD, including enhanced Aβ plaque load, microglial and astrocytic activation, amplified neuroinflammatory responses, and synaptic impairment in cortex and hippocampus. These alterations are not isolated epiphenomena; they represent coordinated, multi‐scale re‐programming of brain‐periphery communication that converges on neurodegeneration. Consequently, MPs exposure qualifies as a bona fide disease‐modifying factor that can convert a slow‐burning amyloidopathy into an aggressive clinical phenotype, offering an experimentally tractable and environmentally relevant mechanism by which the contemporary exposome shapes dementia risk.

Large‐scale metabolomic and microbiome studies have revealed that AD is accompanied by profound remodeling of peripheral metabolism and gut microbial ecology, changes that are increasingly recognized as integral drivers of disease progression rather than secondary consequences [38, 77]. In parallel, accumulating evidence indicates that following ingestion, MPs are not confined to the gastrointestinal tract but disseminate systemically and accumulate across multiple organs, thereby perturbing host physiology at the organismal level [72]. Consistent with this view, recent multi‐omics analyses have demonstrated that MPs' exposure induces widespread metabolic and molecular reprogramming across peripheral and central compartments, alterations that have been increasingly linked to neurotoxic outcomes [76, 78, 79]. However, how MPs intersect with these networks to propel AD remains unclear. Here we show that chronic ingestion of MPs precipitates a coordinated, organism‐wide depletion of taurine that links gut dysbiosis to accelerated AD pathology. Untargeted metabolomics of matched serum and cecal samples identified taurine as the top‐ranked metabolite whose abundance inversely mirrored cognitive performance and Aβ burden in 5XFAD mice. This signature emerged de novo from unsupervised clustering and pathway enrichment, indicating it is a primary, systemic response rather than a downstream epiphenomenon. Mechanistically, taurine loss was coupled to MPs‐induced expansion of taurine‐respiring *Bilophila* and contraction of taurine‐synthesizing *Bifidobacterium* and *Muribaculum*, shifts that collectively down‐regulated microbial sulfur‐amino‐acid pathways. Because mammals possess limited capacity for endogenous taurine synthesis, our data establish the gut microbiota as the obligate gatekeeper of host taurine availability under chronic MPs exposure, and identify taurine as a tractable metabolic lever through which environmental pollutants reshape neurodegenerative trajectories.

ABX‐mediated microbiota depletion and FMT are widely used complementary approaches to directly interrogate the functional involvement of the gut microbiota in host AD‐related phenotypes [40, 47, 80]. We exploited ABX and FMT as reciprocal gain‐ and loss‐of‐function tools to establish causality. ABX completely neutralized the ability of MPs to worsen memory deficits and Aβ pathology in 5XFAD mice, whereas FMT of cecal contents from MPs‐exposed donors into antibiotic‐naïve recipients was sufficient to recapitulate the full spectrum of cognitive and neuropathological deterioration. Thus, MPs operate as indirect, microbiota‐dependent accelerants of neurodegeneration rather than as primary neurotoxic insults.

Previous studies have implicated taurine in a broad spectrum of beneficial biological processes, including enhancement of antitumor immune responses [53], regulation of body weight [54], and extension of lifespan [55, 81]. Mechanistically, taurine has been shown to attenuate oxidative stress, preserve telomere integrity, and improve glucose metabolism, processes that are intimately linked to aging biology and neurodegenerative vulnerability [52, 82]. We therefore asked whether replenishing taurine could break the gut‐brain axis of MPs‐accelerated AD. We found that taurine supplementation significantly mitigated MPs‐exacerbated cognitive dysfunction, amyloid pathology, glial activation, synaptic impairment, and autophagic flux disruption in 5XFAD mice, providing direct evidence that restoring taurine homeostasis is sufficient to counteract the detrimental effects of MPs challenge on the aging brain. In addition, data from the ADNI database showed that plasma taurine level was significantly reduced in individuals with AD compared with cognitively normal participants, and was closely associated with established clinical measures of cognitive impairment. These data elevate taurine from a correlative biomarker to an immediately actionable therapeutic that can be leveraged to neutralize the dementia risk imposed by chronic environmental MPs exposure.

Collectively, our data establish a microbiota‐taurine axis that transduces chronic MPs exposure into accelerated Alzheimer's pathology. By coupling gut‐ecological remodeling to systemic metabolic failure, this pathway offers a mechanistic explanation for how an unavoidable environmental contaminant can converge with intrinsic aging programs to dictate the pace of neurodegeneration.

## 4 Limitations

Several limitations of the present study should be acknowledged when interpreting our findings and considering their broader translational implications. First, while the present study focused on MPs‐exacerbated AD pathology in 5XFAD mice, we acknowledge that the absence of a WT‐MPs group limits our ability to distinguish genotype‐independent neurotoxic effects from disease‐specific exacerbation. Future studies incorporating WT‐MPs controls will be essential to fully characterize the interaction between MPs exposure and genetic susceptibility in AD. Second, while we cannot completely exclude the presence of residual MPs in donor feces, the absence of intestinal barrier damage in FMT recipients—despite robust transfer of cognitive and neuropathological deficits—suggests that the observed effects are primarily driven by transplanted microbiota rather than particulate MPs carryover. Thirdly, while our preclinical findings and ADNI metabolite data support the relevance of taurine dysregulation in AD, the specific alterations in taurine‐associated gut microbiota—including the potential role of *Bilophila*—in human AD patients remain to be determined. Future human microbiome‐metabolome integration studies are warranted to validate the translational relevance of the microbiota‐taurine‐brain axis identified herein. Another limitation is that our evaluation of intestinal barrier integrity was primarily based on selected tight junction markers, including ZO‐1 and additional Occludin assessment, which may not fully capture the broader complexity of gut barrier regulation. Although our data suggest that FMT‐mediated modulation of neurodegenerative phenotypes occurs independently of overt changes in these classical tight junction components, other barrier‐associated pathways—such as additional junctional proteins, epithelial transport systems, mucus layer integrity, immune signaling, or microbial metabolite–host interactions—may also contribute. Therefore, future studies incorporating more comprehensive intestinal permeability measurements and expanded molecular characterization will be important to further dissect microbiota‐dependent mechanisms beyond canonical tight junction disruption in MPs‐associated AD progression.

## Author Contributions

R.L. and Z.W. conceived and designed the study. Z.W. conducted the experiments, performed data analysis and drafted the manuscript. J.Y., M.Z., Y.L. and Z.T. performed the experiments. J.W. provided intellectual contribution. R.L. supervised the study and revised the manuscript. K.G. and J.T.Y. revised the manuscript. All authors reviewed the content and approved the final version for publication.

## Conflicts of Interest

The authors declare no conflicts of interest.

## Supporting information




**Supporting File**: advs76072‐sup‐0001‐SuppMat.docx.

## Data Availability

The 16s rRNA sequencing and untargeted metabolomics data are available from the corresponding author upon request.
