# Supplementary material for: Microplastics‐Induced Gut Microbiota Dysbiosis Accelerates Alzheimer's‐Like Pathology and Cognitive Decline via the Gut–Brain Axis
Source: Adv Sci (Weinh). 2026 Jun 15:e76072. Online ahead of print. doi: 10.1002/advs.76072 (PMC13336413; doi:10.1002/advs.76072)
Supplement: Supplementary file 1 — Supporting File: advs76072‐sup‐0001‐SuppMat.docx. [file ADVS-9999-e76072-s001.docx]

**Supplementary Materials for**

**Microplastics-induced gut microbiota dysbiosis accelerates Alzheimer's-like pathology and cognitive decline via the gut–brain axis**

Zifeng Wu ^1^, Jiarong Yang ^1^, Miaoxuan Zhang ^1^, Yi Li ^1^, Zhuling Tu ^1^, Ji Wang ^1^, Jin-Tai Yu ^2,*^, Kun Guo ^3,*^, Rongcan Luo ^1, *^

1. Gansu Key Laboratory of Biomonitoring and Bioremediation for Environmental Pollution, and Ministry of Education Key Laboratory of Cell Activities and Stress Adaptations, School of Life Sciences, Lanzhou University, 222 South Tianshui Road, Lanzhou 730000, China

2. Department of Neurology, National Center for Neurological Disorders, Huashan Hospital, State Key Laboratory of Medical Neurobiology, MOE Frontiers Center for Brain Science, Shanghai Medical College, Fudan University, Shanghai, China

3. Department of Anatomy and Histology & Embryology, Faculty of Basic Medical Science, Kunming Medical University, Kunming 650500, China

^∗^ Corresponding author, E-mail addresses: luorc@lzu.edu.cn (R. Luo), guokun@kmmu.edu.cn (K.G.), jintai_yu@fudan.edu.cn (J.T.Y)

**This PDF file includes:**

Supplementary Materials and Methods

Figures. S1 to S25

Tables S1 to S6

**Table of Contents:**

1. **Supplementary Materials and Methods**

Animal model

MPs

Characterization of MPs

*SEM*

*DLS analysis*

*FTIR characterization*

*Zeta Potential Measurement*

Preparation of fluorescent MPs and organ fluorescence imaging

Treatment

*Oral gavage of MPs*

*ABX cocktail treatment*

*FMT experiment*

*Drug treatment*

Behavioral tests

*OF test*

*NOR test*

*TCST*

*EPM test*

*MWM test*

Mice tissue collection

Immunofluorescence

Immunohistochemistry

Nissl staining

H&E staining

Western blotting

RNA extraction, cDNA synthesis and RT-qPCR

Cecal contents collection and DNA extraction

16S rRNA library preparation and sequencing

*Amplicon generation*

*Library construction*

*Quality control and sequencing*

16S rRNA microbiome analysis

Untargeted metabolomics of serum

Untargeted metabolomics of cecal contents

Correlation analysis for metabolomics and microbiome

Colony formation assay (CFA)

Subject Participants and clinical assessment

Quantification and statistical analysis

1. **Supplementary Figures and Tables**

Fig. S1. Characterization and distribution of MPs.

Fig. S2. Supplementary behavioral assessments of MPs-exacerbated cognitive deficits in 5XFAD mice.

Fig. S3. MPs treatment exacerbated AD-related pathological features in 5XFAD mice.

Fig. S4. Supplementary analyses revealed that MPs treatment profoundly disrupted the serum metabolome in 5XFAD mice.

Fig. S5. MPs treatment profoundly disrupted the metabolome of cecal contents in 5XFAD mice.

Fig. S7. MPs treatment altered cecal microbiota composition in 5XFAD mice.

Fig. S8. MPs exposure disrupted gut microbial community structure in 5XFAD mice.

Fig. S9. MPs exposure induced significant alterations in microbial functional pathways in 5XFAD mice.

Fig. S10. MPs accumulation in the intestinal tract and the resultant pathological alterations.

Fig. S11. Antibiotic (ABX) treatment effectively depleted gut microbiota in 5XFAD mice.

Fig. S12. Gut microbiota depletion attenuated MPs-exacerbated cognitive and anxiety-like behavioral deficits in 5XFAD mice.

Fig. S13. Gut microbiota depletion abolished MPs-exacerbated AD-related pathology in 5XFAD mice.

Fig. S14. Depletion of gut microbiota abolished MPs-exacerbated neuroinflammation and synaptic dysfunction but not intestinal impairment in recipient mice.

Fig. S15. Validation of gut microbiota depletion and recolonization following ABX treatment and fecal microbiota transplantation (FMT).

Fig. S17. Supplementary behavioral assessments of MPs-exacerbated deficits in 5XFAD mice.

Fig. S17 Gut microbiota is required for MPs-induced Aβ pathology and gliosis.

Fig. S18. FMT from MPs-treated donors exacerbated gliosis, neuroinflammation, and synaptic impairment in recipient mice.

Fig. S19. FMT from MPs-treated donors exacerbated neuronal impairment in recipient mice.

Fig. S20. FMT from donor mice did not alter the intestinal structure of recipient mice.

Fig. S21. Taurine supplementation alleviated MPs-exacerbated cognitive and social behavioral deficits in 5XFAD mice.

Fig. S22. Taurine administration alleviated MPs-exacerbated behavioral deficits in 5XFAD mice.

Fig. S23. Taurine supplementation alleviated MPs-exacerbated synaptic impairment and neuroinflammation in 5XFAD mice.

Fig. S24. Taurine supplementation alleviates MPs-exacerbated intestinal pathology of intestine of 5XFAD mice.

Fig. S25. Decreased plasma taurine level in patients with AD.

Table S1 KEGG pathway enrichment results of the serum metabolomics.

Table S2 Top 50 VIP metabolites in Cluster 4 of serum.

Table S3 Top 50 VIP metabolites in Cluster 4 of cecal contents.

Table S4 KEGG pathway enrichment results of the cecal contents metabolomic.

Table S5. Antibodies used in this study.

Table S6. Primer sequences for RT-qPCR in this study.

**Materials and methods**

**Animal model**

The 5XFAD mice, which overexpress human amyloid precursor protein (APP695) harboring the Swedish (K670N/M671L), Florida (I716V), and London (V717I) mutations, as well as presenilin-1 (PS1) with the M146L and L286V mutations under the control of the Thy-1 promoter, has been described previously^1^. Both genders of 5XFAD mice on a C57BL/6J background, along with their wild-type littermates, were group-housed (5-6 per cage) under specific pathogen-free (SPF) conditions. They were maintained on a 12-h light/dark cycle at approximately 22 ℃ with ~30%-70% humidity. Standard mouse chow and autoclaved water were provided *ad libitum*. Genotyping was performed by PCR analysis of tail biopsy samples using protocols provided by The Jackson Laboratory. Unless otherwise specified, only age-matched mice were used in the experiment. All animal procedures were approved by the Animal Ethics Committee of the School of Life Sciences, Lanzhou University (approval numbers: EAF2024055).

**MPs**

Amino-modified polystyrene microparticles (2 μm diameter; Magsphere, Cat # AM002UM) were reconstituted at 1 mg mL^-1^ in deionized water. The stock suspension was sonicated for 30 min to ensure uniform dispersion and used within 7 days.

**Methods**

**Characterization of MPs**

***SEM***

MPs were characterized for morphology, surface topography, and particle size using SEM (JEOL, JSM-IT500LA). Samples collected on filter paper were transferred onto conductive adhesive tape affixed to specimen stubs, ensuring an even distribution of particles. The specimens were coated with a thin gold layer using an ion sputter coater for 90 s to improve conductivity and enhance imaging quality. SEM imaging was performed at magnifications of 7,500× and 10,000× to capture morphological features at different scales. Particle diameters were measured and size distributions were quantified.

***DLS analysis***

MPs were analyzed for hydrodynamic particle size distribution using DLS. Samples were diluted and dispersed in sterile water, subjected to ultrasonication (100 W, 10 min) to ensure homogeneity, and filtered through a 0.45 µm membrane. Measurements were performed on a DLS instrument (Brookhaven, 90Plus Pals) at a scattering angle of 90°, a constant temperature of 25°C, and a laser wavelength of 658 nm. Each sample was measured three times consecutively with an acquisition time of 60 s per run. Data were processed by the cumulant analysis method to calculate the Z-average hydrodynamic diameter and polydispersity index (PDI).

***FTIR characterization***

MPs were characterized for molecular structure and functional groups using FTIR. Briefly, microsphere suspensions were evenly dispensed onto clean glass culture dishes and dried in an oven at 55°C, followed by additional dehydration under an infrared lamp to remove residual moisture. FTIR spectra were then acquired using an FTIR spectrometer (Nicolet iS50, ThermoFisher) with the following parameters: spectral resolution of 4 cm^-1^, wavenumber range of 400–4000 cm^-1^, and 32 scans accumulated per spectrum. The obtained wavenumber spectra were subsequently used for functional group analysis of MPs.

***Zeta Potential Measurement***

MPs were analyzed for surface charge by determining the Zeta potential using a nanoparticle size and potential analyzer. Samples were prepared as suspensions at 0.02 mg/mL in sterile water that had been pre-filtered through a 0.22 µm membrane to remove impurities. 1 mL of the suspension was carefully drawn using a sterile syringe to avoid air bubble formation and transferred into a disposable cuvette designed for Zeta potential measurements (90Plus Pals, Brookhaven). Measurements were performed at 25°C using a Brookhaven 90Plus PALS analyzer. Each sample was measured in multiple replicates to ensure reproducibility, and the Zeta potential values of MPs were recorded.

**Preparation of fluorescent MPs and** **organ fluorescence imaging**

MPs were fluorescently labeled with Rhodamine B according to the protocol described previously^2^. Fluorescent MPs were prepared by labeling amino-modified polystyrene microspheres with Rhodamine B. The stock solution of MPs was diluted at a 1:100 volume ratio into 50 mL of sterile ultrapure water and dispersed by ultrasonication (200 W, 40 kHz, 30 min) with intermittent cooling on ice to prevent overheating. Subsequently, 300 µL of Rhodamine B solution (0.719 mg/mL; R8040, Solarbio) was added to the suspension, and the reaction vessel was immediately wrapped in aluminum foil to avoid light exposure. The mixture was incubated at 60°C with continuous magnetic stirring (500 rpm) for 12 h to achieve dye loading. The reaction product was collected by vacuum filtration and the retained fluorescently labeled particles were dried in a vacuum oven (70°C, -0.08 MPa, 12 h) to constant weight. The fluorescent MPs were resuspended in sterile water to prepare a 0.02 mg/mL gavage working solution, which was stored at 4°C in the dark until use. All procedures were performed under light-controlled conditions to minimize fluorescence quenching, and all glassware was sterilized by autoclaving before use. 3-month-old 5XFAD mice were exposed to fluorescent MPs for 14 consecutive days. At the end of exposure, mice were sacrificed and the small intestine and large intestine were collected with intact structure and temporarily stored at 4°C. Freshly isolated tissues were promptly transferred to the fluorescence imaging system. Individual tissues were placed in dark sample trays, and *ex vivo* fluorescence imaging was performed using a multimodal *in vivo* imaging system.

**Treatment**

***Oral gavage of MPs***

Both genders of 3-month-old 5XFAD mice were randomly assigned to either the 5XFAD-Ctrl or 5XFAD-MPs group, with age-matched wild-type littermates serving as WT-Ctrl. To preclude cross-contamination of the gut microbiota, each group was housed separately. All procedures followed the ARRIVE guidelines^3^ for the study design. The study was designed to probe the indirect, microbiota-mediated impact of MPs on Alzheimer’s-related pathophysiology. 5XFAD-MPs mice received 10 mg kg⁻¹ day⁻¹ MP suspension by oral gavage for 70 consecutive days; this dose translates to ≈5 g plastic week⁻¹ in humans (human-equivalent dose calculated with Km correction) and aligns with previously published rodent exposure regimens ^4-6^. WT-Control and 5XFAD-Control mice received an equivalent volume of sterile water via the same route. The specific onset times and total duration of behavioral testing are detailed in the timelines accompanying each corresponding section.

***ABX cocktail treatment***

To deplete the gut microbiota, 3-month-old 5XFAD mice were randomly assigned to either 5XFAD-ABX or 5XFAD-ABX + MPs group. All animals remained under SPF housing and received an oral ABX cocktail (vancomycin 0.5 mg/ml, gentamicin 1 mg/ml, ampicillin 1 mg/ml, neomycin 1 mg/ml, metronidazole 1 mg/ml) dissolved in sterile water, as previously described ^1^. The mice were given with cocktail daily for 7 consecutive days, followed by a 3-day resting period to minimize acute toxicity, and then maintained by gavage every 48 h. Fecal samples at the end of the depletion phase were collected and used for OD_600_ measurements and a CFA experiment which will be discussed in the following section. Mice in the 5XFAD-ABX + MPs group received daily MP gavage beginning immediately after the initial 7-day antibiotic course. Behavioral tests were performed during the last period of treatment, after which animals were anesthetized and euthanized for downstream analyses.

***FMT experiment***

Previous studies reported that a stable remodeling of the gut microbiota necessitates a continuous treatment period of 3-4 weeks^7,8^. Donor mice (5XFAD-Ctrl and 5XFAD-MPs mice which were mentioned above) were housed in SPF conditions to establish a stable gut microbial community. For gut microbiota isolation, fresh feces were collected from donor mice and immediately dispersed in pre-cooled, sterile PBS solution to avoid a shift in the microbiota composition or loss of bacterial viability. The fecal suspension was vigorously shaken and vortexed until the particles were completely disrupted, with intermittent cooling on ice to keep microbiota stable. Following this, the fecal suspension was soon centrifuged (300 g, 4℃, 5 min) and the supernatant was collected into a new centrifuge tube and mixed with an equal volume of 50% glycerol in PBS to keep the microbiota stable^9^. To deplete all the microbes, the fecal supernatant was subjected to steam sterilization using an autoclave at 121℃ for 20 minutes. Overall, the fecal supernatant was prepared to administer to mice from each group.

For the FMT experiment, 3-month-old 5XFAD mice and littermates were grouped as: (A) 5XFAD-heat-killed treated (HT) to WT; (B) 5XFAD to WT; (C) 5XFAD-HT to 5XFAD; (D) 5XFAD to 5XFAD and (E) 5XFAD-MPs to 5XFAD. All mice were housed in SPF conditions and administered the ABX cocktail orally for 7 consecutive days as previously described. Fecal samples from the stage of gut microbiota depletion were collected for OD_600_ analysis and a CFA experiment after gut microbiota depletion followed by a rest for 3 days. All mice were administered fecal supernatant daily via oral gavage and fresh feces were collected from recipient mice on day 10 to perform OD_600_ analysis and a CFA experiment to evaluate the colony efficacy of the gut microbiota from donor mice. We conducted animal behavioral tests during the last period of oral gavage followed by anesthesia and euthanasia to perform subsequent experiments.

***Drug treatment***

Previous work demonstrated that oral taurine at 500 or 1000 mg kg^-1^·d^-1^ protects C57BL/6J mice from aging-related decline ^10^. Based on the maximum solubility of taurine (25 mg/mL in H_2_O according to the information of MCE), we selected 500 mg kg⁻¹ day⁻¹ for the present study. Taurine [HY-B0351, MCE] was dissolved in sterile water (25 mg mL^-1^) with brief sonication and administered by oral gavage.

Three-month-old 5XFAD mice were randomly assigned to 5XFAD-Ctrl, 5XFAD-MPs or 5XFAD-MPs + Taurine groups, and wild-type littermates served as WT-Ctrl. Mice in the 5XFAD-MPs + Taurine group received taurine in the morning and MPs in the afternoon; those in the 5XFAD-MPs group received MPs alone; controls received an equivalent volume of sterile water.

**Behavioral tests**

Mice were moved to the behavioral laboratory before the first assay and remained there throughout the battery to ensure environmental habituation. The animal behavioral tests were performed in the fixed order: OF test, NOR test, TCST, EPM test, MWM test, with a 24 h inter-test interval to prevent carry-over effects.

***OF test***

Anxiety-like behavior and spontaneous locomotor activity of mice in the open field test were recorded, tracked and evaluated according to the protocol described previously^11^. The apparatus consisted of a hard plastic open-field box (40 × 40 × 40 cm), with ambient illumination maintained at 50 lux. After 30 min of room habituation, mice were placed in the center and allowed to explore for 15 min while being video-tracked from above using AnyMaze (v 7.37) behavioral tracking software (RRID:SCR_014289, Stoelting Co). Total distance, time and distance in center vs. periphery, and mean speed were extracted. Trials ran between 09:00–17:00 in a sound-attenuated chamber; the arena was wiped with 75 % ethanol between subjects and the experimenter remained silent to eliminate olfactory and auditory confounds.

***NOR test***

Recognition memory was assessed in a 40 × 40 × 40 cm grey Plexiglas arena (50 lux)^1^. The procedure was conducted over three consecutive days: On the first day (habituation), each mouse was individually placed in the empty arena and allowed to explore freely for 10 min. On the second day (familiarization), two identical wooden cubes (5 × 5 × 5 cm) were symmetrically positioned within the arena, and the animal was permitted to explore freely for 10 min. On the third day (test session), one of the cubes on the left side was replaced with a wooden cylinder of equal volume (5 cm in diameter, 7 cm in height), and exploration behavior was recorded for another 10 min. Trials ran between 09:00–17:00 in a sound-attenuated room; the arena and objects were cleaned with 75% ethanol between mice. Behavioral activity was recorded using an overhead video tracking system and analyzed with AnyMaze. Exploration time—defined as the duration during which the mouse’s nose was within 2 cm of the object and oriented toward it—was quantified, and the Recognition Index was calculated to assess recognition memory and preference for the novel object. All tests were conducted under quiet conditions to minimize environmental disturbance.

***TCST***

Social behavior and social memory were evaluated using the three-chamber social test according to the protocol described previously^12^. The apparatus consisted of a transparent acrylic box divided into three interconnected chambers (overall dimensions: 60× 40× 40 cm; each chamber: 20 cm × 40 cm × 40 cm). The test comprised three consecutive phases: during the first phase (habituation), empty wire cages (10 cm in diameter, 15 cm in height) were placed in the two side chambers, and the test mouse was positioned in the central chamber with access to all compartments for 5 min to explore freely. During the second phase (sociability test), a novel unfamiliar mouse (stranger 1, S1) was confined in one of the wire cages, while the opposite cage remained empty; the test mouse was allowed to explore for 10 min. In the third phase (social novelty test), a new unfamiliar mouse (stranger 2, S2) was placed in the previously empty cage while S1 remained in place, and exploration behavior was recorded for another 10 min. All experiments were conducted under uniform illumination of 50 lux. The apparatus and wire cages were thoroughly cleaned with 75% ethanol between sessions and trials to remove residual odor cues. Behavioral data were recorded using an overhead video tracking system and analyzed with AnyMaze. Duration of sniffing—defined as the duration during which the mouse’s nose was within 2 cm of the cage and oriented toward it within a 60° angle—along with the social preference index were automatically calculated to evaluate sociability and social recognition. All unfamiliar mice were habituated to the wire cages for 30 min prior to testing and were matched to test mice by strain, age, and sex. All procedures were carried out in a sound-attenuated behavioral room under controlled environmental conditions.

***EPM test***

Fear and anxiety states of mice were evaluated and analyzed by the elevated plus maze test. The apparatus was elevated 100 cm above the ground and consisted of a central platform (6 × 6 cm) with two closed arms (30 × 6 × 15 cm) and two open arms (30 × 6 cm), with ambient illumination maintained at 50 lux. During the test, each mouse was gently placed in the center of the central platform and allowed to explore freely for 10 min. All sessions were recorded in real time using a video camera mounted above the apparatus, and the recordings were analyzed using AnyMaze behavioral tracking software to quantify duration of sniffing, time spent in open arm and time spent in closed arm. All tests were conducted under quiet conditions to minimize environmental disturbance and all equipment was thoroughly cleaned with 75% ethanol between trials to eliminate odor cues.

***MWM test***

Spatial learning and memory abilities of mice were assessed using the MWM test according to the previous study^13^. Briefly, the MWM test was conducted in a circular pool (120 cm in diameter and 50 cm in depth) filled with opaque water maintained at 20 ± 1℃. A circular escape platform (10 cm in diameter) was submerged 1 cm below the water surface. To minimize visual interference, the water surface was evenly covered with light-colored plastic balls, and the water was replaced every three days to maintain cleanliness.

The experiment consisted of a 7-day acquisition training phase followed by spatial probe tests. During the training phase, mice were subjected to four trials per day, each initiated from one of four fixed starting points (southeast, northeast, southwest, and northwest quadrants). The intertrial interval was 20–40 min to allow adequate rest while preventing overlearning or memory decay. For each trial, the mouse was released facing the wall of the pool and allowed 60 s to locate the hidden platform. If the mouse failed to find the platform within the allotted time, it was gently guided to it and allowed to remain there for 30 s. Four hours after completion of the final training session on day 7, a short-term memory probe test was performed by removing the platform and recording swimming trajectories for 60 s. A long-term memory probe test was conducted 72 h later under the same conditions. All experiments were performed under uniform illumination (50 lux) in a quiet testing room. Behavioral data—including time of first crossing, platform location crosses, mean speed, distance moved in the target quadrant, and time spent in the target quadrant—were automatically recorded and analyzed using AnyMaze software. Video tracking was conducted with an overhead camera system, and stable distal visual cues were maintained around the pool to provide spatial references throughout the experiment.

**Mice tissue collection**

Mouse anesthesia and tissue sampling were performed following standard procedures as previously described^11,13^. After behavioral testing, mice were deeply anesthetized with sodium pentobarbital (60 mg kg⁻¹, i.p.; P3761, Sigma) and were verified to be unresponsive to noxious stimuli. Transcardial perfusion was performed with ice-cold 0.1 M PBS (5 mL min⁻¹) until the outflow was clear (10–15 mL). Tissue samples were then harvested in the following order: 1. *Cecal contents*: the cecum was ligated at both ends, excised aseptically, opened longitudinally, and the contents were transferred to sterile cryovials; 2. *Intestinal tissues*: A 2-cm duodenal segment (distal to the pylorus) and the proximal colon (1 cm distal to the cecum) were isolated, gently flushed with PBS, and processed for fixation or snap-freezing; 3. *Brain tissue*: The skull base was opened and the whole brain removed intact for regional dissection. Fresh tissues were flash-frozen in liquid nitrogen and stored at -80°C. Intestinal segments were fixed in pre-cooled 4% paraformaldehyde (PFA), and brains were processed according to downstream assay requirements. All steps were performed on ice with instruments cleaned in 75% ethanol and RNase decontaminant to prevent RNA degradation and cross-contamination.

**Immunofluorescence**

Cerebral hemispheres and intestinal tissues (duodenum and colon) were fixed in 4% PFA (30525-89-4, Macklin), cryoprotected by sequential immersion in 15% and 30% sucrose (57-50-1, Macklin) in PBS at 4℃ until the tissues sank. Cryoprotected tissues were embedded in OCT compound (Cat # 4583, Sakura) and sectioned at 30 µm on a cryostat (FS800A, RWD). Sections were collected and stored at -20°C in cryoprotectant (glycerol: PBS = 7:3) until use. For staining, free-floating sections were rinsed in PBS containing 0.1% Triton X-100 (BS084, Biosharp). Brain sections were blocked with 5% bovine serum albumin (BSA; 9048-46-8, Macklin) in PBST for 2 h at room temperature; intestinal sections were blocked with 10% goat serum (BL210A, Biosharp) for 1.5 h. All sections were then incubated with the respective primary antibodies (Table S5) diluted in PBST containing 2% BSA overnight at 4°C. The sections were then washed three times in PBST, and incubated with fluorophore-conjugated goat anti-rabbit secondary antibodies (SA00013-4, Proteintech) for 1.5 h at room temperature in the dark. Nuclei were counterstained with 4',6-diamidino-2-phenylindole (DAPI; C1006, Beyotime), washed in PBST, and mounted in glycerol: PBS (7:3). Coverslips were sealed and slides were stored at 4°C until imaging on a Leica Stellaris 5 confocal microscope.

**Immunohistochemistry**

After 48 h post-fixation in 4% PFA, hemispheres were dehydrated through a graded ethanol series (50%, 70%, 80%, 95%, and 100% ethanol), cleared in xylene (1330-20-7, Guangnuo) and infiltrated with molten paraffin (8002-74-2, Biosharp) in a 65℃ oven (three changes). Tissues were oriented in embedding molds and sectioned at 6 μm on a rotary microtome. Ribbons were floated on a 40℃ water-bath, mounted on charged slides and dried overnight at 37℃. For immunohistochemical staining, 6 μm coronal slices were de-paraffinized in xylene and rehydrated through a graded ethanol series (100%, 95%, 80%, and 70%). Antigen retrieval was carried out in 10 mM sodium citrate, pH 6.0 (BL619A, Biosharp) at 95℃ for 20 min. Endogenous peroxidase was blocked with 3% H₂O₂ in methanol (67-56-1, Fuyu chemical) for 15 min at room temperature. Non-specific binding was blocked with 5% normal goat serum in PBS containing 0.3% Triton X-100 for 1 h at room temperature, followed by overnight incubation at 4°C with primary antibodies against Aβ, GFAP and IBA1(Table S5). After washing with PBS, sections were incubated with HRP-conjugated secondary antibody (5450-0010 (474-1506), Seracare) for 1 h at room temperature and visualized with DAB substrate (ZLI-9017, Zhongshanjinqiao). Slides were dehydrated, cleared in xylene and mounted with a neutral synthetic resin. Images were acquired on an Olympus VS200 bright-field slide scanner.

**Nissl staining**

Paraffin-embedded coronal sections were baked at 60 °C for 1 h, then deparaffinized and rehydrated: xylene (3 × 3 min), 100% ethanol (2 × 3 min), 85% ethanol (3 min), 75% ethanol (3 min), distilled water. Sections were stained in Nissl solution (G1036, Servicebio) for 2 min, rinsed gently in distilled water, and differentiated in 0.1% acetic acid for 1 s. Rapid dehydration followed (75%, 85%, 95%, and 100% ethanol, 5 s each), cleared in xylene (2 × 5 s), and coverslipped with neutral synthetic resin. Images were acquired on an Olympus VS200 bright-field slide scanner.

**H&E staining**

Deparaffinized and rehydrated sections of brain and intestine were incubated in hematoxylin (G1005-1, Servicebio) for 30 s, rinsed in distilled water, and differentiated in 1% acid alcohol for 5 s. After bluing in 1% ammonia water (10 s), sections were dehydrated through 70%, 80%, and 95% ethanol (15 s each), counterstained in eosin (G1100, Solarbio) for 2 s, rapidly dehydrated in absolute ethanol (2 × 5 s), cleared in xylene (2 × 5 s) and mounted with neutral synthetic resin. Images were captured on an Olympus VS200 bright-field slide scanner.

**Western blotting**

Western blotting was performed as described in our previous study^13^. Tissues were homogenized on ice in RIPA buffer (R0010, Solarbio) supplemented with PMSF (ST506, Beyotime) at a 1:10 (w/v) ratio. After 30 min at 4°C, lysates were centrifuged at 12,000 rpm for 15 min at 4°C, and the supernatant was collected. Protein concentration was determined with a BCA kit (PC0022, Solarbio). Samples were adjusted to equal concentrations, mixed with 5× SDS-PAGE loading buffer (P1040, Solarbio) and denatured at 95°C for 10 min. Equal amounts (25 μg/lane) of protein were separated by 12% sodium dodecyl sulfate polyacrylamide gel electrophoresis and transferred to a polyvinylidene difluoride membrane (ISEQ00010, Millipore). The membranes were blocked with 5% non-fat milk (100-04504, Bio-rad) for 2 h at room temperature, incubated overnight at 4°C with primary antibodies (Table S5), washed three times with TBST (Tris buffered saline [Cell Signaling Technology, 9997] with Tween-20 [0.1%; Sigma, P1379]) (5 min each) and probed with HRP-conjugated secondary antibodies (1:10,000; 5450-0010 (474-1506), Seracare) for 2 h at room temperature. The epitope was visualized using an ECL chemiluminescence kit (PK10003, Proteintech) and imaged on a Shenhua SH-Magic523 system. Band intensities were quantified using ImageJ (Fiji) software, normalized to GAPDH, and analyzed using GraphPad Prism 9.5.

**RNA** **extraction, cDNA synthesis and RT-qPCR**

Total RNA was isolated from 10-20 mg frozen tissue using TRIzol (RK145, Tiangen) under RNase-free conditions. Tissue powder (liquid nitrogen) was lysed in 1 mL TRIzol, incubated on ice for 20-30 min, and centrifuged (12,000 × g, 10 min, 4°C). The supernatant was mixed with 200 µL chloroform, vortexed for 15 s, incubated for 5 min, and centrifuged (12,000 × g, 15 min, 4℃). The aqueous phase was transferred and mixed with 1:1 isopropanol, incubated for 15 min, and centrifuged (12,000 × g, 10 min, 4 °C). The pellet was washed twice with 75% ethanol (DEPC-water), air-dried, and dissolved in 30–50 µL RNase-free water. RNA purity (A260/280 ≥ 1.8), concentration (Nanodrop 2000, Thermo) and integrity (1% agarose gel) were verified. All RNA samples were aliquoted and stored at -80℃ until further use.

For cDNA synthesis, 2 µg of RNA was reverse-transcribed in 25 µL with M-MLV reverse transcriptase (M1708, Promega) and random primers (PC2440, Solarbio). The RNA-primer mixture was pre-incubated at 70℃ for 5 min to activate the primers and denature secondary structures, followed by immediate cooling on ice. The reaction mixture was incubated at 37℃ for 60 min and subsequently heated at 70℃ for 5 min to terminate the reaction. cDNA was stored at −20℃ for subsequent RT-qPCR. RT-qPCR was performed with SYBR Green PCR Master Mix (4913914001, Roche) on an Archimed-X4 system (ROCGENE). Each 20 µL reaction contained 10 µL of SYBR Green, 1 µL each primer (10 µM), 1 µL cDNA, and 7 µL nuclease-free water. Cycling: 95 °C 30 s; 40 cycles of 95 °C 5 s, 60 °C 30 s; melt-curve 65–95 °C. All samples were run in triplicate, and no-template and no-reverse-transcription controls were included to confirm the absence of contamination or genomic DNA. Primer efficiency (90–110%) and single-peak melting curves were confirmed. Relative gene expression levels were calculated by 2^(−ΔΔCt)^14^ with *Gapdh* as reference. Primer sequences used for RT-qPCR are listed in Table S6.

**Cecal contents collection and DNA extraction**

Cecal contents were snap-frozen in liquid nitrogen within 2 min of collection and stored at -80°C (seven samples each for WT-Ctrl and 5XFAD-MPs, eight for 5XFAD-Ctrl). Total genomic DNA was extracted with the DNA isolation kit (KG203, Tiangen) under sterile, nuclease-free conditions. Concentration and purity (A260/A280 1.8–2.0) were determined with a NanoDrop 2000 (Thermo), and integrity was verified on a 1.2% agarose gel stained with nucleic acid dye (3590A, Takara). High-molecular-weight DNA was aliquoted and stored at -80 °C until sequencing.

**16S rRNA library preparation and sequencing**

Amplicon generation

The V3–V4 region of the 16S rRNA gene was amplified with universal primers that incorporated sample-specific 8-nt barcodes at the 5’ end. PCR was performed with Q5® High-Fidelity DNA polymerase (M0491L, NEB) for 25 cycles using an identical thermal profile for all samples. Negative (no-template) controls were processed in parallel; any run with detectable amplification in the negative control was discarded. Amplicons were purified with VAHTS™ DNA Clean Beads (Vazyme) and quantified with the Quant-iT™ PicoGreen® dsDNA kit (P7589, Invitrogen) on a FLx800 microplate reader (BioTek). Samples were pooled in equimolar amounts based on the fluorescence readings.

Library construction

Pooled amplicons were converted to Illumina libraries with the TruSeq Nano DNA LT Library Prep Kit. End-repair, A-tailing, adapter ligation (indexed adapters), and 8-cycle library enrichment were carried out according to the manufacturer’s protocol. Adapter dimers and short inserts were removed with two consecutive AMPure XP (Beckman Coulter) clean-ups. Final libraries were validated on a 2% agarose gel and stored at -20°C.

Quality control and sequencing

Library insert size and concentration were determined with an Agilent Bioanalyzer using the High Sensitivity DNA kit; only libraries showing a single sharp peak at 450–500 bp and absence of adapter dimers were accepted. Quantified libraries (> 2 nM) were combined into a single sequencing pool, denatured with 0.2 N NaOH, and loaded onto an Illumina MiSeq instrument at 10 pM with 15% PhiX spike-in. Paired-end 2 × 300 bp reads were generated with the MiSeq Reagent Kit v3 (600 cycles), yielding ≥ 30,000 merged reads per sample.

**16S rRNA microbiome analysis**

Raw paired-end reads generated from Illumina sequencing were processed using QIIME2 ^15^, followed by sequence length distribution analysis performed by R (v 4.3.3). We then performed taxonomic classification in QIIME2 (v 2019.4). Greengenes (Release 13.8)^16^ or SILVA (Release 132)^17^ were used for read alignment and classification. Sequences lacking adequate reference coverage were labeled as unassigned, and those with insufficient resolution were annotated as unclassified. To standardize sequencing depth across samples, amplicon sequence variants (ASV)/operational taxonomic units (OTU) abundance tables were rarefied using QIIME2 (v 2019.4). Rarefaction was performed with the qiime feature-table rarefy function at 95% of the minimum sample depth to minimize data loss while maintaining comparability. The rarefied table was used for downstream alpha and beta diversity analyses performed in R (v 4.3.3) with the phyloseq and vegan packages.

**Untargeted metabolomics of serum**

Metabolite extraction and detection were performed at Biotree Co., Ltd. (Shanghai, China). Serum was collected in pre-cooled sterile EP tubes (seven samples for WT-Ctrl and 5XFAD-MPs groups and eight for 5XFAD-Ctrl group) and snap-frozen in liquid nitrogen and stored at -80℃.

For metabolite extraction, 100 μL of serum was mixed with 400 μL of pre-chilled methanol/acetonitrile (1:1, v/v), vortexed for 30 s, followed by sonication for 10 min in an ice-water bath, and incubated at -20 °C for 1 h. After centrifugation at 14,000 × g for 15 min at 4℃, the supernatants were collected for analysis. Equal aliquots of supernatants from all samples were pooled to generate quality control (QC) samples, which were analyzed alongside experimental samples.

Polar metabolites were analyzed using an ultra-high-performance liquid chromatography (UHPLC) system (Vanquish, Thermo Fisher Scientific) equipped with a Waters ACQUITY UPLC BEH Amide column (2.1 × 50 mm, 1.7 μm; Waters). The mobile phase consisted of solvent A (aqueous solution containing 25 mmol/L ammonium acetate and 25 mmol/L ammonia) and solvent B (acetonitrile). The autosampler temperature was maintained at 4℃, and the injection volume was 2 μL. Mass spectrometry data acquisition was performed on an Orbitrap Exploris 120 mass spectrometer (Thermo Fisher Scientific) operated using Xcalibur software (version 4.4; Thermo Fisher Scientific). Both full MS and MS/MS spectra were acquired in positive and negative electrospray ionization modes using the following parameters: sheath gas flow rate, 50 Arb units; auxiliary gas flow rate, 15 Arb units; capillary temperature, 320℃; full MS resolution, 60,000; MS/MS resolution, 15,000; normalized stepped collision energy, 20/30/40; spray voltage, 3.8 kV (positive mode) or -3.4 kV (negative mode). Raw LC–MS data were converted to mzXML format using ProteoWizard and processed using collaboratively developed R packages for metabolite identification against the BiotreeDB database. Subsequent data visualization and statistical analyses were performed using in-house R scripts. The dataset comprised four QC samples and 28 experimental samples, from which a total of 30,356 metabolic features were extracted.

**Untargeted metabolomics of cecal contents**

Cecal contents were collected in pre-cooled sterile EP tubes (seven samples for WT-Ctrl and 5XFAD-MPs groups and eight for 5XFAD-Ctrl group), snap-frozen in liquid nitrogen and stored at -80℃. For metabolite extraction, 25 mg of each sample was transferred into Eppendorf tubes, followed by the addition of 500 μL of extraction solvent (methanol/acetonitrile/water, 2:2:1, v/v/v) containing isotope-labeled internal standards. Samples were vortexed for 30 s, homogenized at 35 Hz for 4 min and subsequently sonicated in an ice-water bath for 5 min; this homogenization–sonication cycle was repeated three times. After incubation at -40 °C for 1 h to precipitate proteins, samples were centrifuged at 12,000 rpm (13,800 × g, rotor radius 8.6 cm) for 15 min at 4 °C. The supernatants were transferred to autosampler vials for LC–MS/MS analysis. QC samples were prepared by pooling equal aliquots of supernatants from all samples. Untargeted metabolomic profiling and subsequent data processing and visualization were performed using the same analytical pipeline as described for serum metabolomics. The dataset comprised four QC samples and 28 experimental samples, from which a total of 42,824 metabolic features were extracted.

**Correlation analysis for metabolomics and microbiome**

We performed a correlation analysis between metabolomics and microbiome, focusing on metabolites and bacteria to explore the relationship between molecules and microorganisms that may be different among WT-Ctrl, 5XFAD-Ctrl and 5XFAD-MPs groups according to a previous study^15^. Pearson’s correlation coefficients were calculated between the relative abundances of microbial taxa and raw metabolite values using R (V4.3.3), followed by association analyses among differentially abundant microorganisms.

**CFA**

For qualitative assessment of culturable bacteria, LB agar plates were prepared by dissolving 25 g/L LB broth (L001, MDBio) and 15 g/L agar powder (A8190, Solarbio) in deionized water, followed by autoclaving at 121°C for 20 min. After cooling to 55°C, the medium was poured into Petri dishes and solidified at room temperature. Fresh fecal supernatants extracted from mice were kept on ice and subjected to serial 10-fold dilutions in sterile PBS. Aliquots (100 μL) of dilutions ranging from 10^-3^ to 10^-5^ were spread evenly onto pre-solidified LB plates using sterile glass spreaders. Plates were air-dried for 15 min, inverted, and incubated at 37°C for 24-48 h. Colony formation was documented using a digital imaging system under standardized lighting and magnification conditions.

**Participants and clinical assessment**

Data used for this study were obtained from the ADNI-1 cohort, accessed through the ADNI database (adni.loni.usc.edu)^18^. Participants with available taurine-related data were included in the cohort for subsequent analyses (CN = 226 and AD = 185). Concentration of taurine (nM) in plasma was compared between CN and AD, followed by linear regression analyses performed to evaluate the relationship between plasma taurine levels and clinical indicators (ADAS-cog, CDR, MMSE). For each specific analysis, participants lacking the corresponding metric were excluded.

**Quantification and statistical analysis**

The experimental details were indicated in each figure legend. Statistical tests used for group or cluster comparisons in metabolomics, microbiome analyses and correlation analysis were specified in the relative sections of the methods. Each *n* represented an independent biological sample. No statistical methods were used to predetermine sample sizes, but our sample sizes were comparable to those used in a previously published study^1,13,15^. Data were presented as the mean ± the standard error of the mean (SEM). Prior to statistical analysis, data were assessed for normality using the Shapiro-Wilk test and for homogeneity of variance using the Brown-Forsythe test. For normally distributed data with equal variances, a two-tailed Student’s *t*-test was used for two-group comparisons, one-way ANOVA with Tukey’s *post hoc* test for multiple comparisons, and two-way ANOVA with Tukey's *post hoc* test for analyses involving two independent variables. For datasets violating parametric assumptions, Mann-Whitney U test or Kruskal-Wallis test with Dunn's *post hoc* test was applied. The statistical test used for each analysis was indicated in the corresponding figure legend. Statistical analyses were performed using GraphPad Prism (v 9.5) and R (v 4.3.3). The statistical test used and *P* values were indicated in each figure legend. ^*^*P* < 0.05, ^**^ *P* < 0.01, ^***^ *P* < 0.001. Commercial databases, including KEGG (http://www.genome.jp/kegg/) and MetaCyc (https://metacyc.org/), were used for the pathway enrichment analysis.

**Fig. S1.**

**
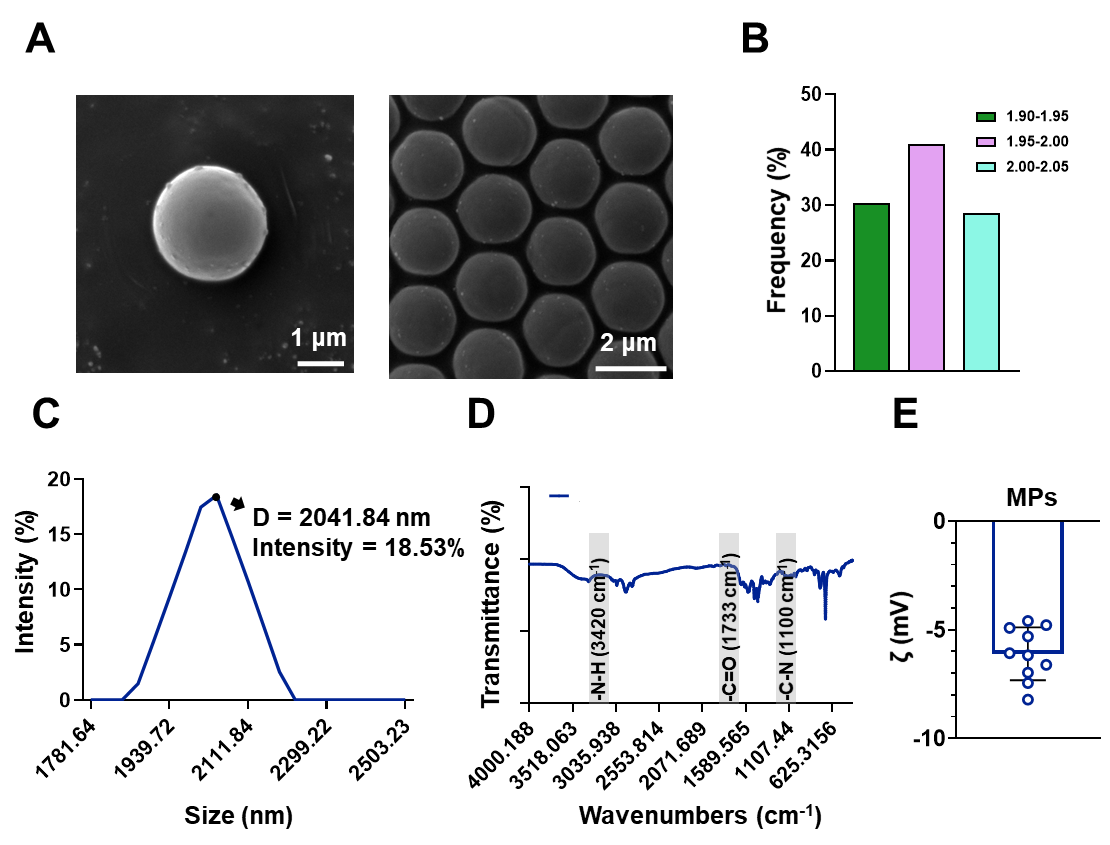
**

**Fig. S1. Characterization and distribution of MPs.** (A) Representative scanning electron microscopy (SEM) images of microplastics (MPs). Scale bar: 1 μm (*left* panel) or 2 μm (*right* panel). (B) Size distribution of MPs as determined from SEM images (n = 150 particles). (C) Zeta potential of MPs dispersed in sterile water (n = 10 measurements). (D) Infrared spectrum of MPs and three characteristic absorption peaks are displayed. (E) Particle size distribution measured by dynamic light scattering (DLS). Data are presented as mean ± SD. All experiments were performed in triplicate with representative results shown.

**Fig. S2.**


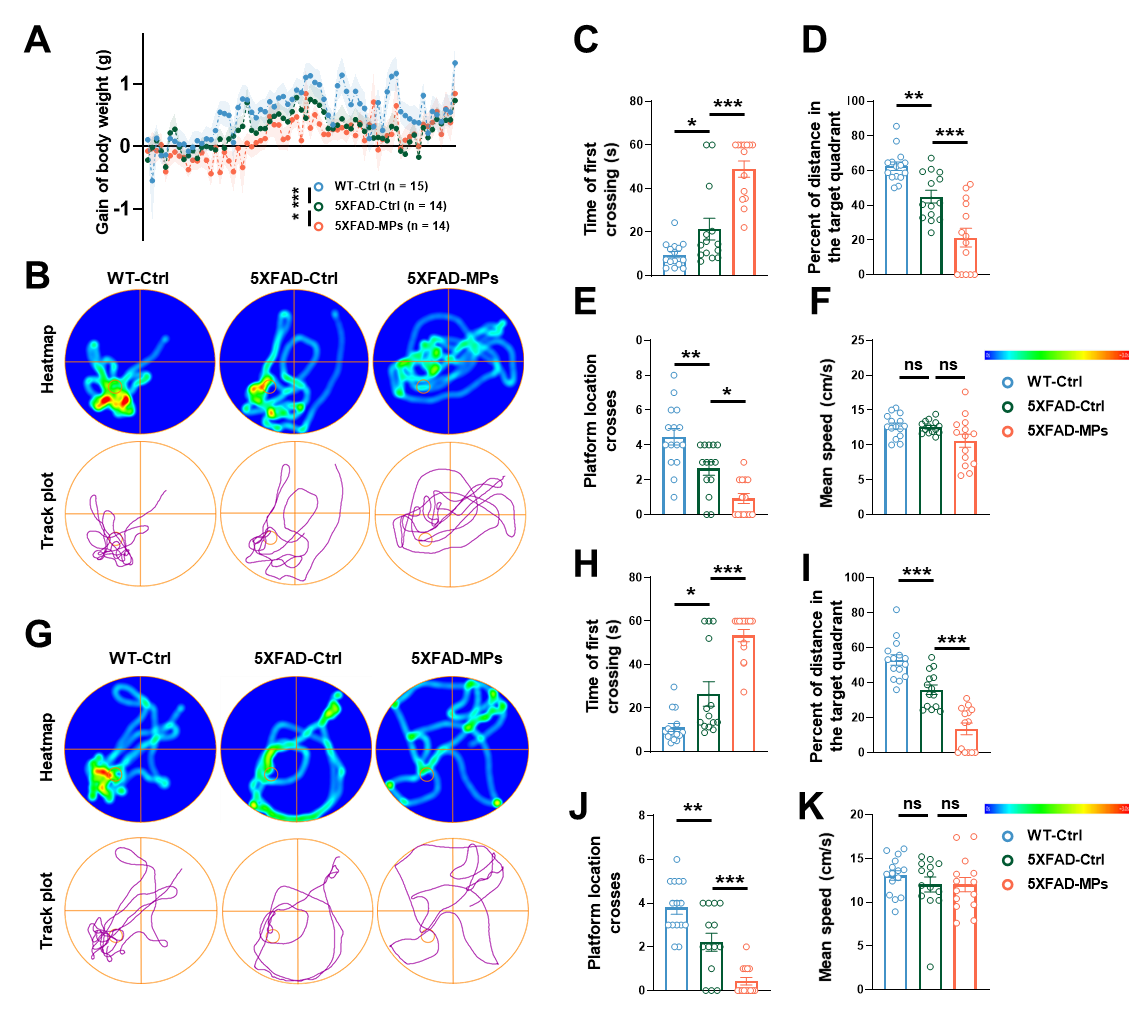


**Fig. S2. Supplementary behavioral assessments of MPs-exacerbated cognitive deficits in 5XFAD mice.** (A) Body weight gain across all groups. (B) Representative heatmaps and track plots during the 4 h probe trial of the Morris water maze (MWM) test for each group. (C-F) Quantitative analysis of the 4 h MWM probe trial: (C) time of first crossing (s), (D) percent of distance in the target quadrant, (E) crosses of platform location and (F) mean swimming speed (cm/s). (G) Representative heatmaps and track plots during the 72 h probe trial of MWM test for each group. (H-K) Quantitative analysis of the 72 h MWM probe trial: (H) time of first crossing (s), (I) percent of distance in the target quadrant, (J) crosses of platform location and (K) mean swimming speed (cm/s). The group sizes were n = 15, 14, and 14 for the WT-Ctrl, 5XFAD-Ctrl, and 5XFAD-MPs groups, respectively. The WT-Ctrl group included 8 males and 7 females, while the other two groups each included 7 males and 7 females. Each data point represents an individual mouse. Data are presented as mean± SEM. ns, not significant; **P* < 0.05, ***P* < 0.01, ****P* < 0.001 (one-way ANOVA with Tukey’s post hoc test in C-F and H-K; Two-way ANOVA with Tukey’s *post hoc* test in A).

**Fig. S3.**


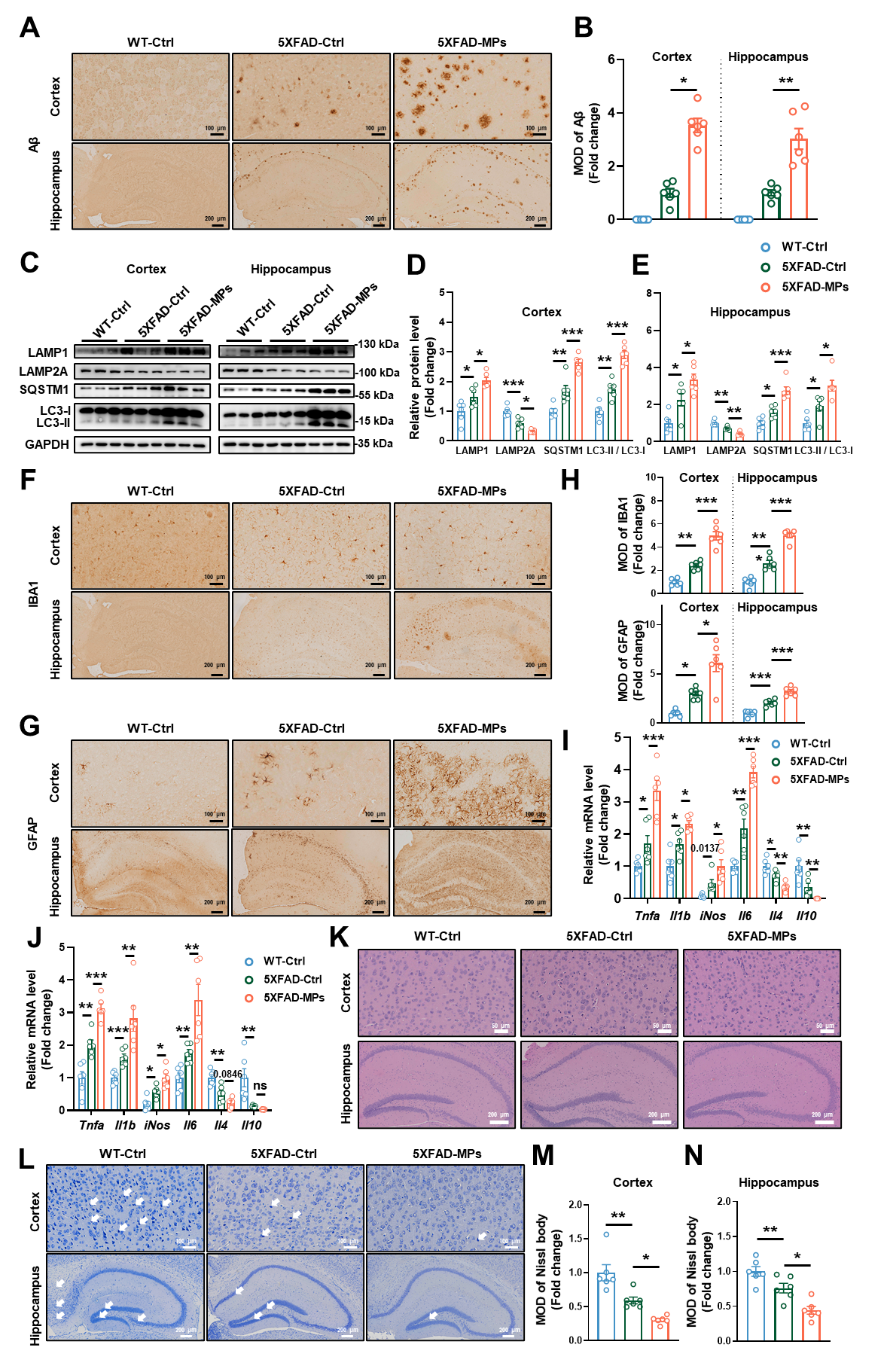


**Fig. S3. MPs treatment exacerbated AD-related pathological features in 5XFAD mice.** (A-B) Amyloid-β (Aβ) deposition in the cortex and hippocampus: (A) Representative immunohistochemistry (IHC) images and (B) quantitative analysis. (C-E) Autophagy-related protein levels: (C) representative western blot results and quantitative analysis in the (D) cortex and (E) hippocampus. (F-H) Neuroinflammation markers: (F) representative IHC images and (H) quantitative analysis of IBA1 mean optical density (MOD) in the cortex and hippocampus; (G) representative IHC images and (H) quantitative analysis of GFAP MOD in the cortex and hippocampus. (I-J) Relative mRNA expression of inflammatory cytokines in the (I) cortex and (J) hippocampus: pro-inflammatory (*Il6*, *Tnfa*, *Il1b*, *inos*) and anti-inflammatory cytokines (*Il4* and *Il10*). (K) Representative hematoxylin and eosin (H＆E) staining images of the cortex and hippocampus. (L-N) Nissl staining: (L) representative images of the cortex and hippocampus (white arrows indicate Nissl bodies) and quantitative analysis of Nissl body MOD in the (M) cortex and (N) hippocampus. n = 6 for each group. Each data point represents an individual mouse. Data are presented as mean ± SEM. ns, not significant; **P*< 0.05, ***P* < 0.01, ****P* < 0.001 (one-way ANOVA with Tukey’s *post hoc* test in B, D, E, I, J, M and N).

**Fig. S4.**


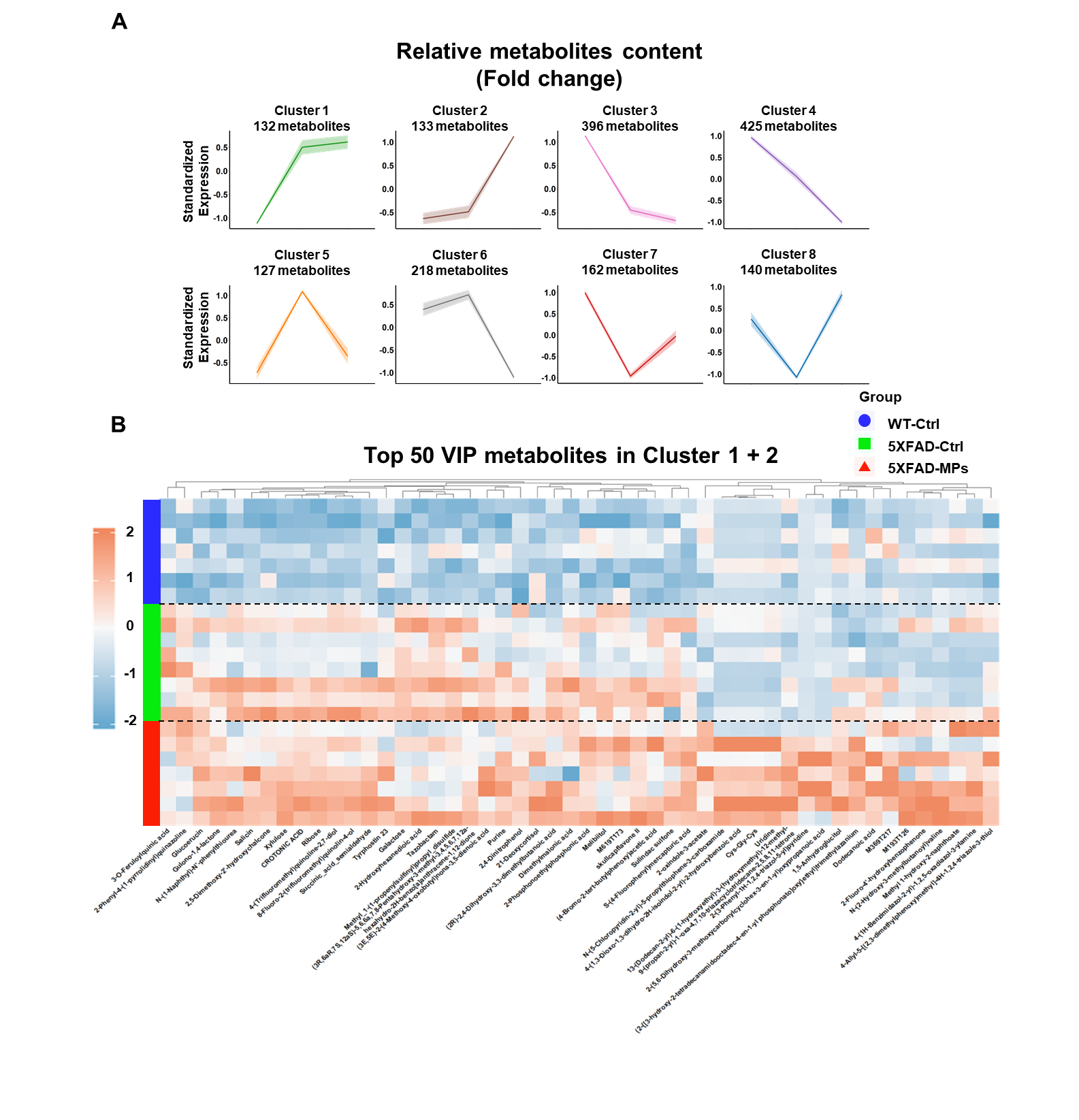


**Fig. S4. Supplementary analyses revealed that MPs treatment profoundly disrupted the serum metabolome in 5XFAD mice.** (A) Unsupervised clustering of serum metabolites based on standardized values across the three experimental groups. Metabolites were grouped into eight clusters according to their temporal expression patterns. Lines represent mean profiles, and shaded areas indicate SEM. The number of metabolites in each cluster is indicated. (B) Heatmap of the top 50 metabolites ranked by VIP scores from PLS-DA in Cluster 1 and Cluster 2. Data were Z-score normalized across samples. Colors indicate relative metabolite abundance. n = 7, 8, 7 for the WT-Ctrl, 5XFAD-Ctrl, 5XFAD-MPs groups, respectively.

**Fig. S5.**


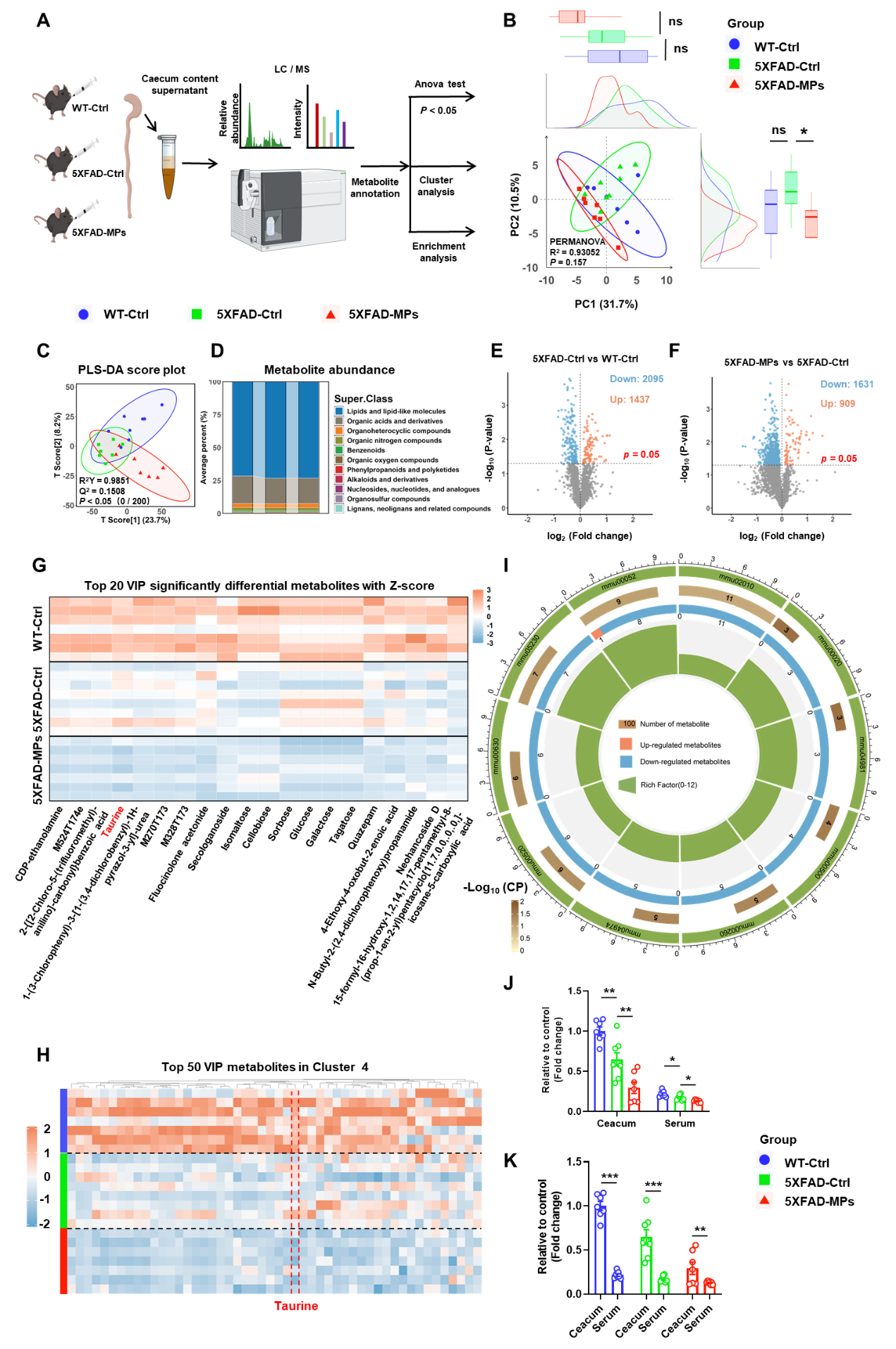


**Fig. S5. MPs treatment profoundly disrupted the metabolome of cecal contents in 5XFAD mice.** (A) Schematic overview of the experimental workflow for caecum content metabolomics. (B) Principal component analysis (PCA) score plot showing the distribution of caecum metabolomic profiles among groups. Marginal boxplots and density plots indicate the distribution along PC1 and PC2. (C) Partial least squares discriminant analysis (PLS-DA) score plot of caecum metabolomic profiles among groups. (D) Relative abundance of metabolite superclasses in caecum contents across groups. (E, F) Volcano plots showing differential metabolites (E) between 5XFAD-Ctrl and WT-Ctrl, and (F) between 5XFAD-MPs and 5XFAD-Ctrl. Dashed lines indicate the significance threshold (p = 0.05). (G) Heatmap showing Z‑score‑normalized abundances of the top 20 significantly differential metabolites, ranked by VIP scores. (H) Heatmap of the top 50 VIP metabolites in Cluster 3 and Cluster 4. Data were Z-score normalized across samples. (I) KEGG pathway enrichment analysis of differential metabolites, showing the number of metabolites, up- and down-regulated metabolites, and enrichment significance. (J, K) Relative levels of representative metabolites in caecum contents and serum, expressed as fold change relative to WT-Ctrl. n = 7, 8, 7 for the WT-Ctrl, 5XFAD-Ctrl, 5XFAD-MPs groups, respectively. Data are shown as mean ± SEM. Statistical significance is indicated (**P* < 0.05, ***P* < 0.01, ****P* < 0.001).

**Fig. S6.**


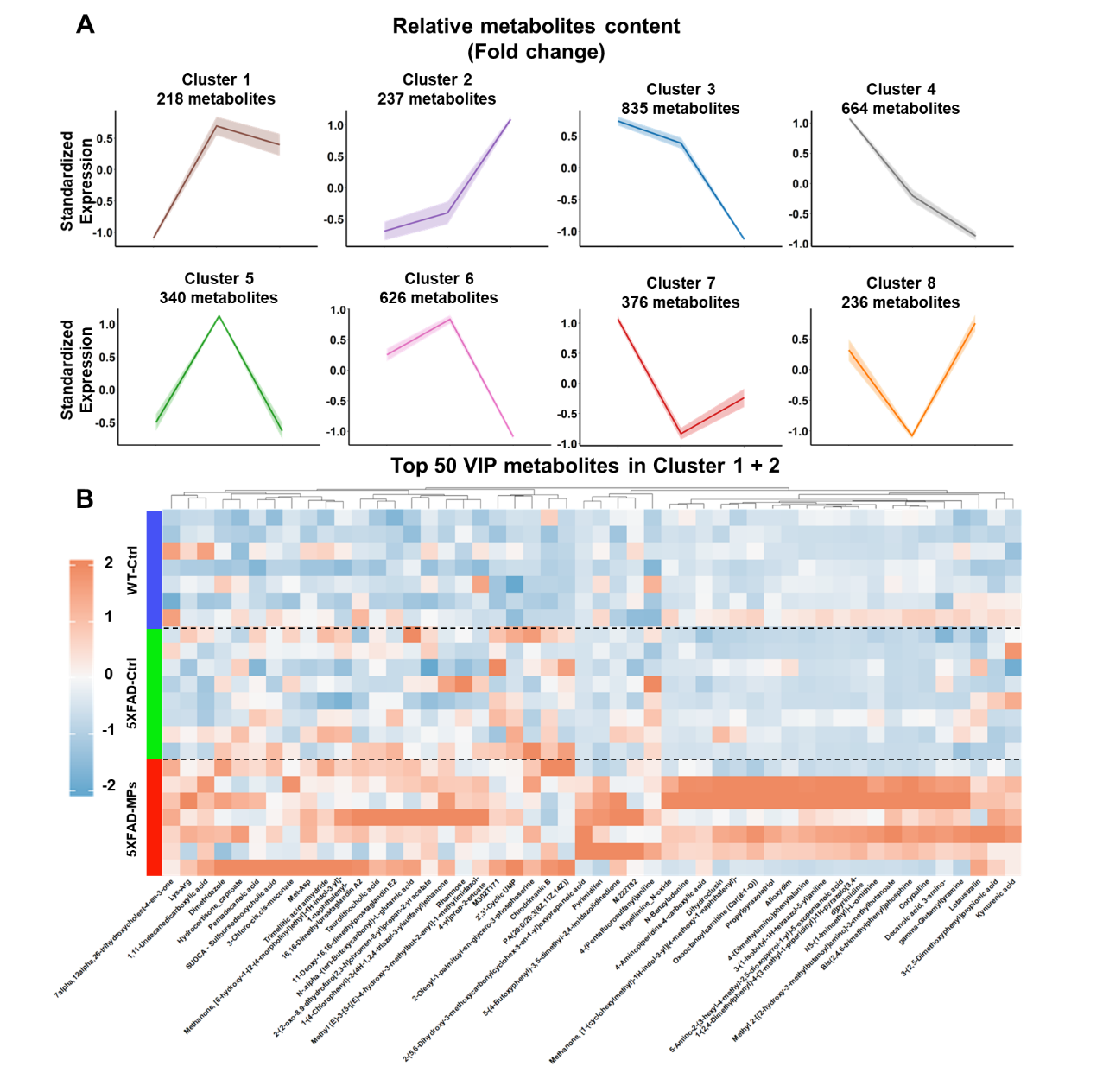


**Fig. S6. Supplementary analyses revealed that MPs treatment profoundly disrupted the metabolome of cecal contents in 5XFAD mice.** (A) Unsupervised clustering of cecal metabolites based on standardized values across the three experimental groups. Metabolites were grouped into eight clusters according to their temporal expression patterns. Lines represent mean profiles, and shaded areas indicate SEM. The number of metabolites in each cluster is shown. (B) Heatmap of the top 50 metabolites ranked by VIP scores from PLS-DA in Cluster 1 and Cluster 2. Metabolite abundances were Z-score normalized across samples. n = 7, 8, 7 for the WT-Ctrl, 5XFAD-Ctrl, 5XFAD-MPs groups, respectively.

**Fig. S7.**


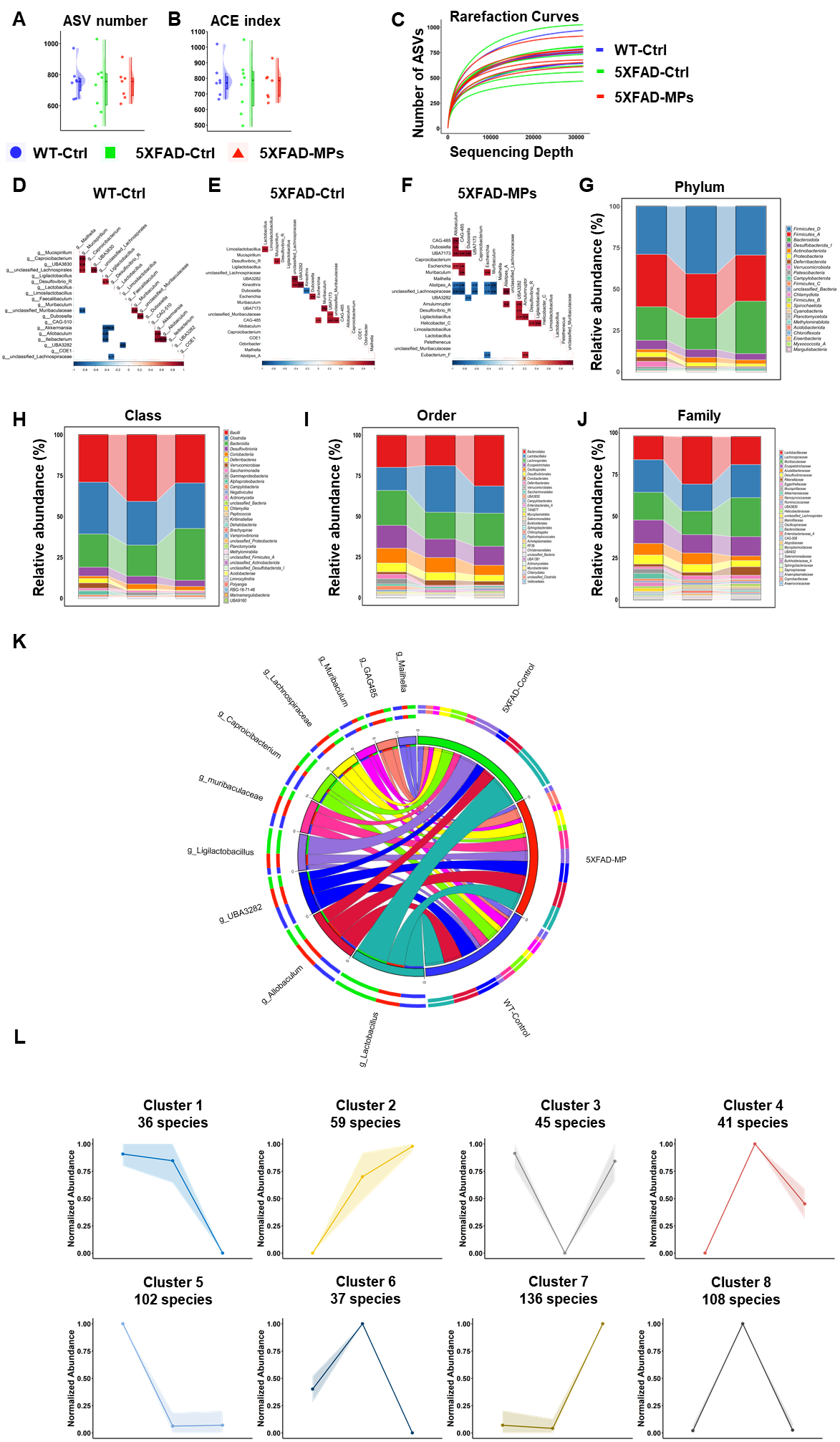


**Fig. S7. MPs treatment altered cecal microbiota composition in 5XFAD mice.** (A-B) alpha diversity comparison: (A) amplicon sequence variant (ASV) number and (B) abundance-based coverage estimator (ACE) index across all groups. Box plots display the interquartile range (25th–75th percentile) with the median indicated by a horizontal line; whiskers represent minimum and maximum values. Statistical comparison: one-way ANOVA with Tukey’s *post hoc* test. (C) Rarefaction curves of microbiome for individual mice in each group. (D-F) Correlation analysis of genus-level taxa across groups. Color scale indicates positive (red) or negative (blue) correlations between microbial taxa. (G-J) Relative abundance of microbial taxa at different levels: (G) Phylum, (H) Class, (I) Order and (J) Family. Data are presented as average percentage. (K) Circos plot illustrating the representative genera across all three groups. (L) K-means clustering analysis identified eight distinct microbial clusters. Numbers in parentheses indicate the number of species in each cluster. Each n represents a mouse. n = 7, 8, 7 for the WT-Ctrl, 5XFAD-Ctrl, 5XFAD-MPs groups, respectively.

**Fig. S8.**


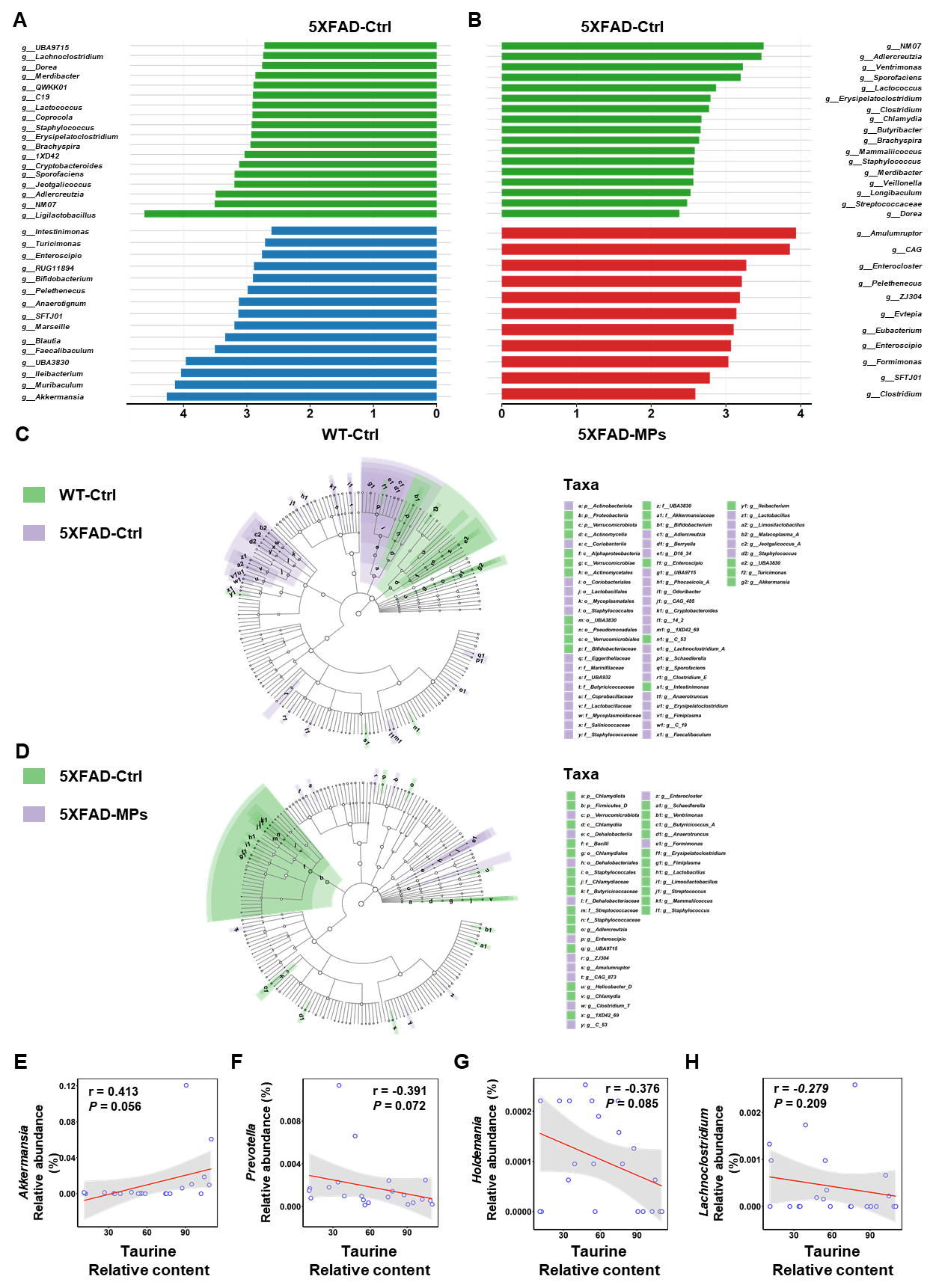


**Fig. S8. MPs exposure disrupted gut microbial community structure in 5XFAD mice.** (A-B) Linear discriminant analysis effect size (LEfSe) analysis showing significantly differentially abundant genera: (A) WT-Ctrl versus 5XFAD-Ctrl and (B) 5XFAD-Ctrl versus 5XFAD-MPs. Linear discriminant analysis (LDA) scores were calculated for each genus. (C-D) Cladograms illustrating the phylogenetic distribution of differentially abundant microbial taxa in 5XFAD mice (C: WT-Ctrl vs. 5XFAD-Ctrl; D: 5XFAD-Ctrl vs. 5XFAD-MPs). Circle size represents relative abundance; color indicates enrichment in the respective group. (E-H) Pearson correlation analysis between taurine level and the relative abundance of representative genera identified by LEfSe analysis. Correlation coefficients (r) and *P* values are indicated.

**Fig. S9.**


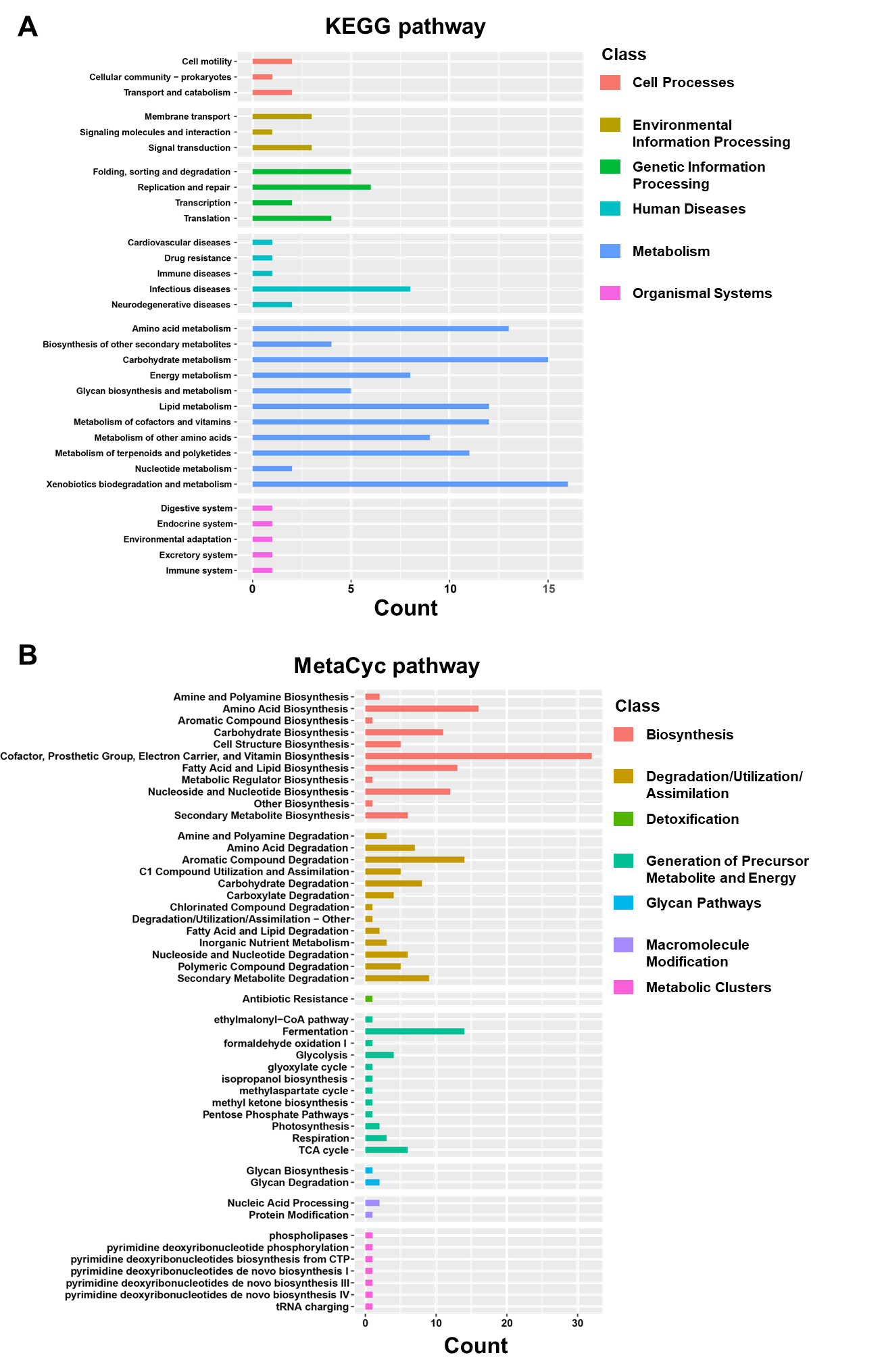


**Fig. S9. MPs exposure induced significant alterations in microbial functional pathways in 5XFAD mice.** (A) Kyoto Encyclopedia of Genes and Genomes (KEGG) pathway analysis of gut microbiota. (B) MetaCyc metabolic pathway analysis of gut microbiota.

**Fig. S10.**


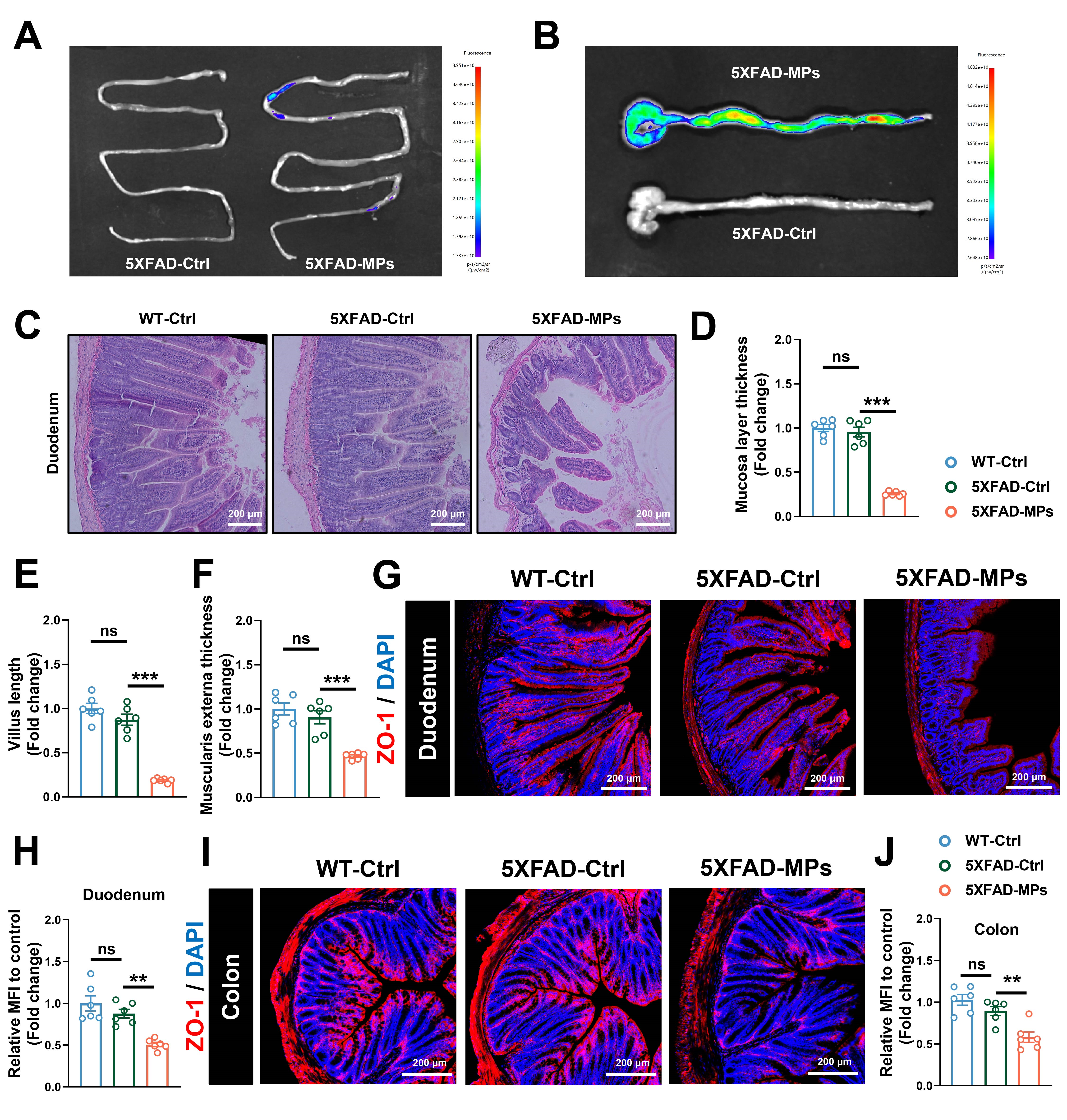


**Fig. S10. MPs accumulation in the intestinal tract and the resultant pathological alterations.** (A-B) Fluorescence signal was detected in the (A) small intestine and the (B) large intestine. (C) Representative H＆E images of the duodenum of mice in each group. (D-F) Quantitative analysis of the mucosa layer thickness, villus length and muscularis externa thickness of the duodenum (n = 6). (G, I) Representative fluorescence images of ZO-1 in the (G) duodenum and (I) colon of mice in each group. (H, J) Quantitative analysis and comparison of the MFI of ZO-1 among the three groups in the (H) duodenum and (J) colon (n = 6). Each point represented a mouse. Data are presented as mean ± SEM. ns, not significant; ***P* < 0.01, ****P* < 0.01 (one-way ANOVA with Tukey’s *post hoc* test in D-F, H and J).

**Fig. S11.**


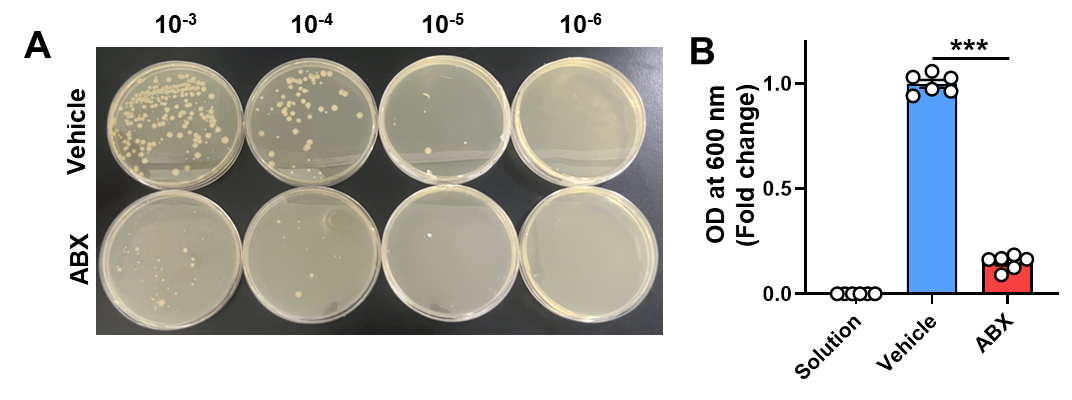


**Fig. S11. Antibiotic (ABX) treatment effectively depleted gut microbiota in 5XFAD mice.** (A) Representative colony-forming assay (CFA) images of fecal homogenates from 5XFAD mice following ABX administration, showing markedly suppressed bacterial growth on Luria-Bertani (LB) agar plates. (B) Optical density at 600 nm (OD_600_) measurements of fecal homogenates confirming a significant reduction in total bacterial load after ABX treatment (n = 6 per group). Each data point represents an individual mouse. Data are presented as mean ± SEM. ****P* < 0.001 (one-way ANOVA with Tukey’s *post hoc* test in B).

**Fig. S12.**


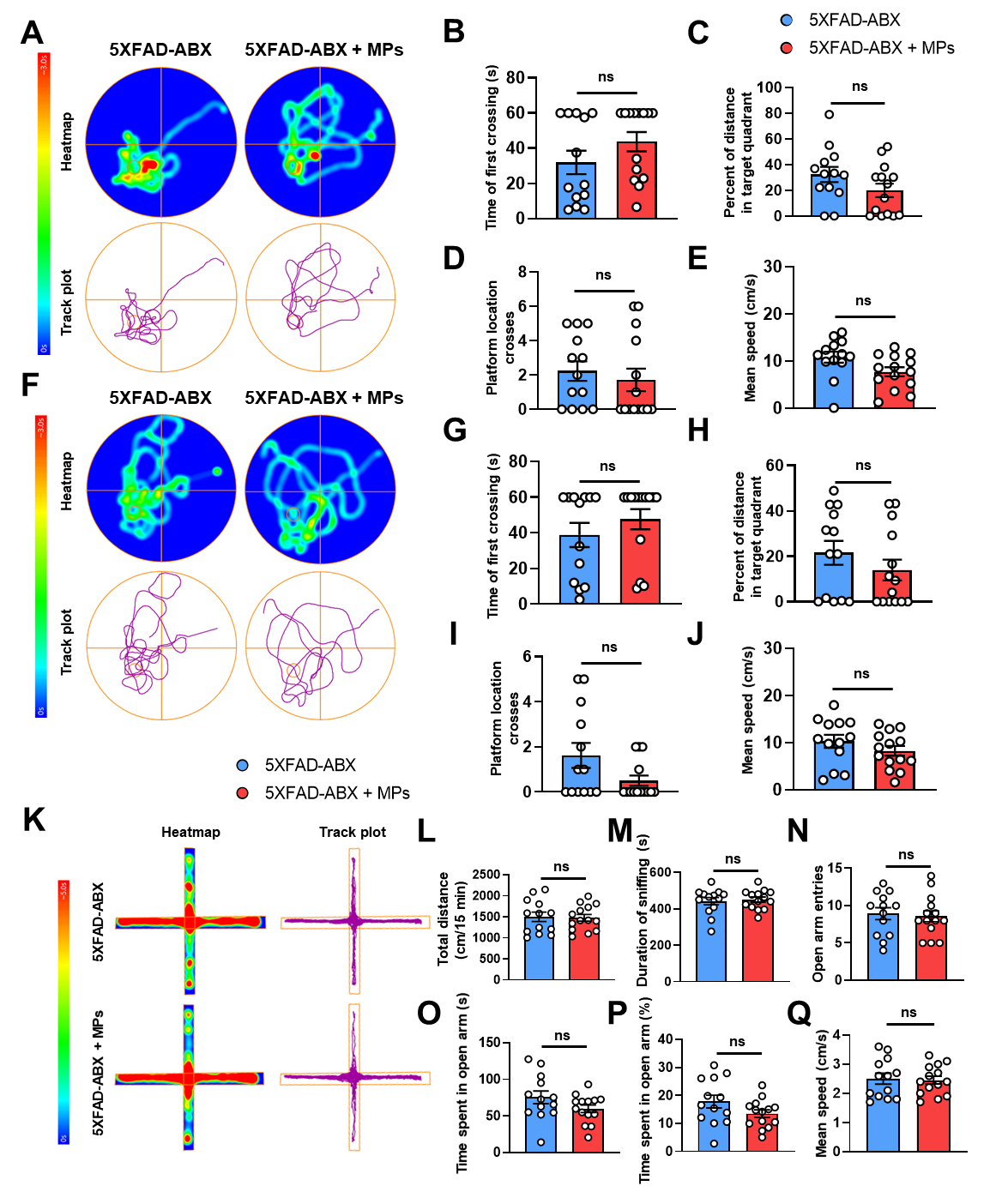


**Fig. S12. Gut microbiota depletion attenuated MPs-exacerbated cognitive and anxiety-like behavioral deficits in 5XFAD mice.** (A) Representative heatmaps and track plots during the 4 h probe trial of the MWM test for each group. (B-E) Quantitative analysis of the 4 h MWM probe trial: (B) time of first crossing (s), (C) percent of distance in the target quadrant, (D) crosses of platform location and (E) mean swimming speed (cm/s). (F) Representative heatmaps and track plots during the 72 h probe trial of MWM test for each group. (G-J) Quantitative analysis of the 72 h MWM probe trial: (G) time of first crossing (s), (H) percent of distance in the target quadrant, (I) crosses of platform location and (J) mean swimming speed (cm/s). (K) Representative heatmaps and track plots in the elevated plus maze (EPM) test for each group. (L-Q) Quantitative analysis of EPM parameters: (L) total distance, (M) duration of sniffing, (N) open arm entries, (O) time spent in open arm (s), (P) time spent in open arm (%) and (Q) mean speed. The group sizes were n = 13 for the 5XFAD‑ABX group (7 males and 6 females) and n = 14 for the 5XFAD‑ABX+MPs group (7 males and 7 females). Each point represents an individual mouse. Data are presented as mean ± SEM. ns, not significant (unpaired Student’s *t*-test in B-E, G-J, L-Q).

**Fig. S13.**


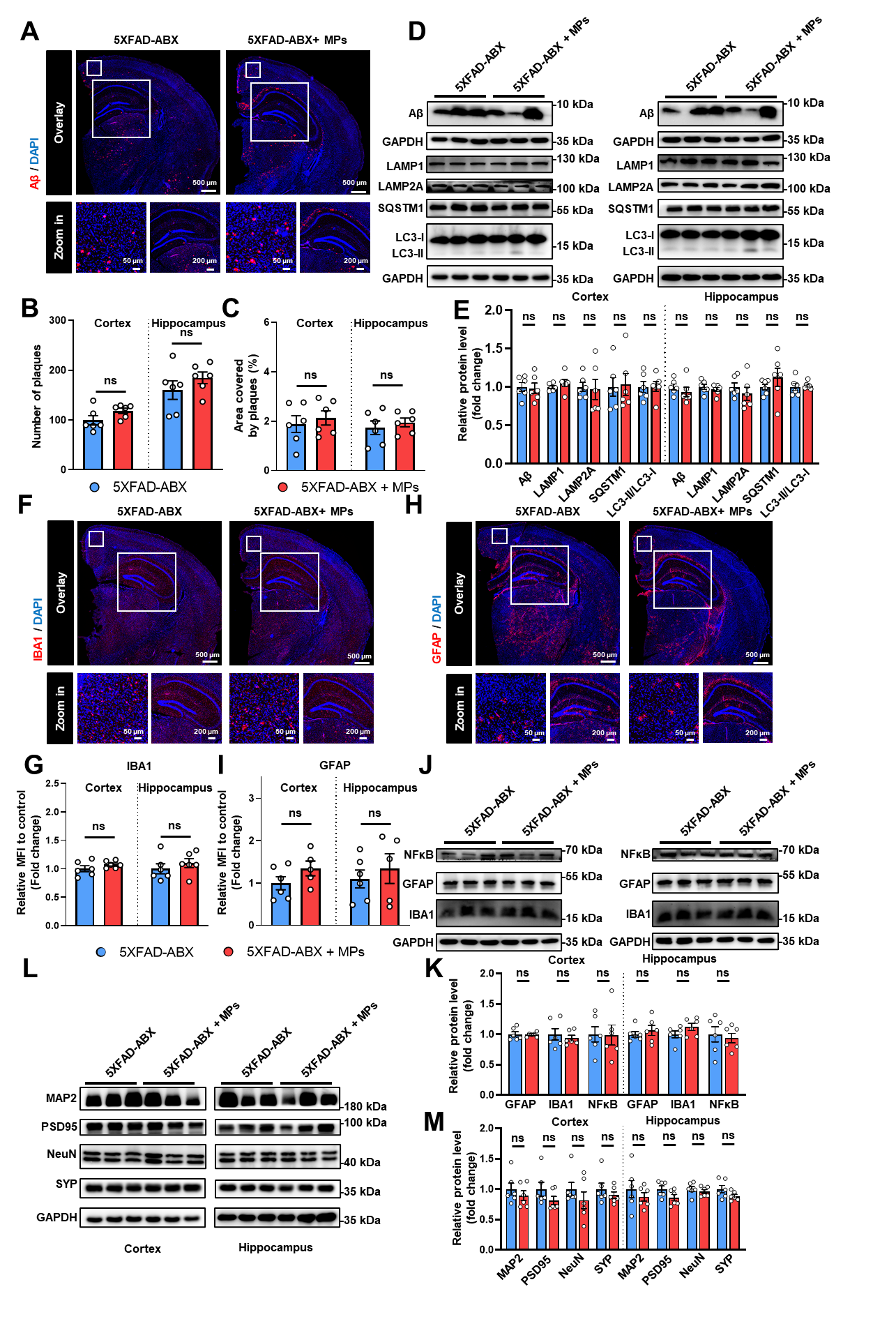


**Fig. S13. Gut microbiota depletion abolished MPs-exacerbated AD-related pathology in 5XFAD mice.** (A) Representative immunofluorescence images showing Aβ deposition (red) with DAPI counterstaining (blue) in the cortex and hippocampus of ABX-treated 5XFAD mice with or without concurrent MPs administration. (B-C) Quantification of Aβ burden in the cortex and hippocampus: (B) plaque number and (C) total plaque area. (D-E) Western blot analysis of Aβ and autophagy-related proteins (LAMP1, LAMP2A, SQSTM1, and LC3-II/LC3-I ratio) in cortical and hippocampal tissues: (D) representative western blot images and (E) quantitative analysis of Aβ and autophagy-related protein levels. (F-G) Microglial activation: (F) representative immunofluorescence images and (G) quantitative analysis of IBA1 mean fluorescence intensity (MFI) in the cortex and hippocampus. (H-I) Astrocytic activation: (H) representative immunofluorescence images and (I) quantitative analysis of GFAP MFI in the cortex and hippocampus. (J-K) Neuroinflammation and glial activation markers: (J) representative western blot results and (K) quantitative analysis of protein levels in the cortex and hippocampus. (L-M) Neuronal and synaptic integrity markers: (L) representative western blot results and (M) quantitative analysis of protein levels in the cortex and hippocampus. n = 6 per group. Each data point represents an individual mouse. Data are presented as mean ± SEM. ns, not significant (unpaired Student’s *t*-test in B, C, E, G, I, K, M).

**Fig. S14.**


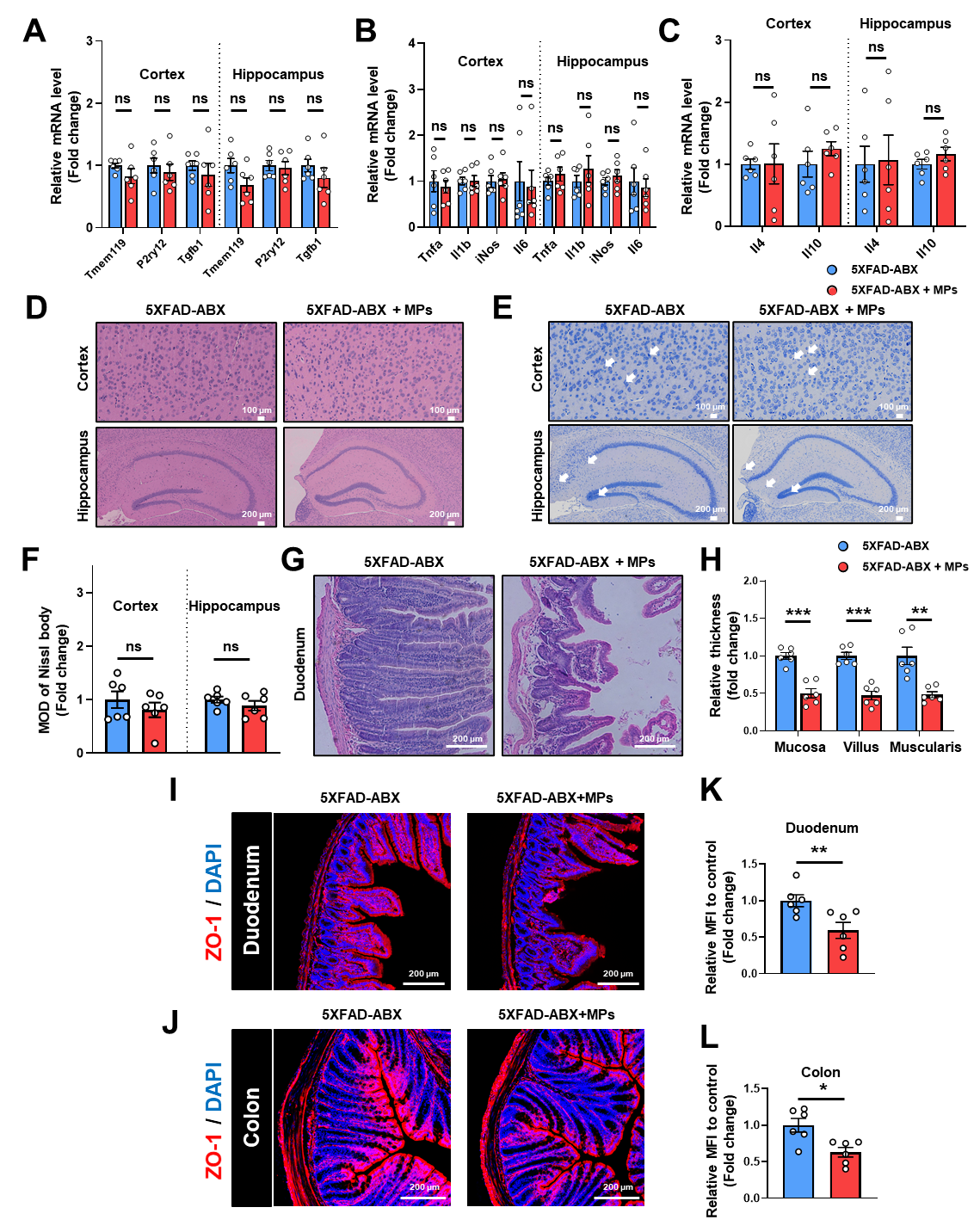


**Fig. S14. Depletion of gut microbiota abolished MPs-exacerbated neuroinflammation and synaptic dysfunction but not intestinal impairment in recipient mice (n = 6).** (A) Relative quantitative analysis of microglial homeostasis- and phagocytosis-related genes in the cortex and hippocampus between the two groups. (B-C) Relative quantitative analysis of (B) pro-inflammatory (*Il6*, *Tnfa*, *Il1b*, *inos*) and (C) anti-inflammatory cytokines (*Il4* and *Il10*) in the cortex and hippocampus between the two groups. (D) Representative H＆E staining images in the cortex and hippocampus of ABX-treated 5XFAD mice. (E) Representative Nissl staining images of the cortex and hippocampus in ABX-treated 5XFAD mice of each group, with white arrows indicating Nissl bodies. (F) Quantification of the MOD of Nissl bodies in the cortex and the hippocampus. (G) Representative H＆E images of the duodenum of mice in each group. (H) Quantitative analysis of the mucosal layer thickness, villus length and muscularis externa thickness of the duodenum. (I-J) Representative fluorescence images of ZO-1 in (I) the duodenum and (J) the colon of mice in each group. (K-L) Quantitative analysis and comparison of the MFI of ZO-1 among the three groups in (K) the duodenum and (L) colon. Each point represents a mouse. Data are presented as mean ± SEM. ns, not significant; **P* < 0.01, ***P* < 0.01, ****P* < 0.001 (Unpaired *t*-test).

**Fig. S15.**


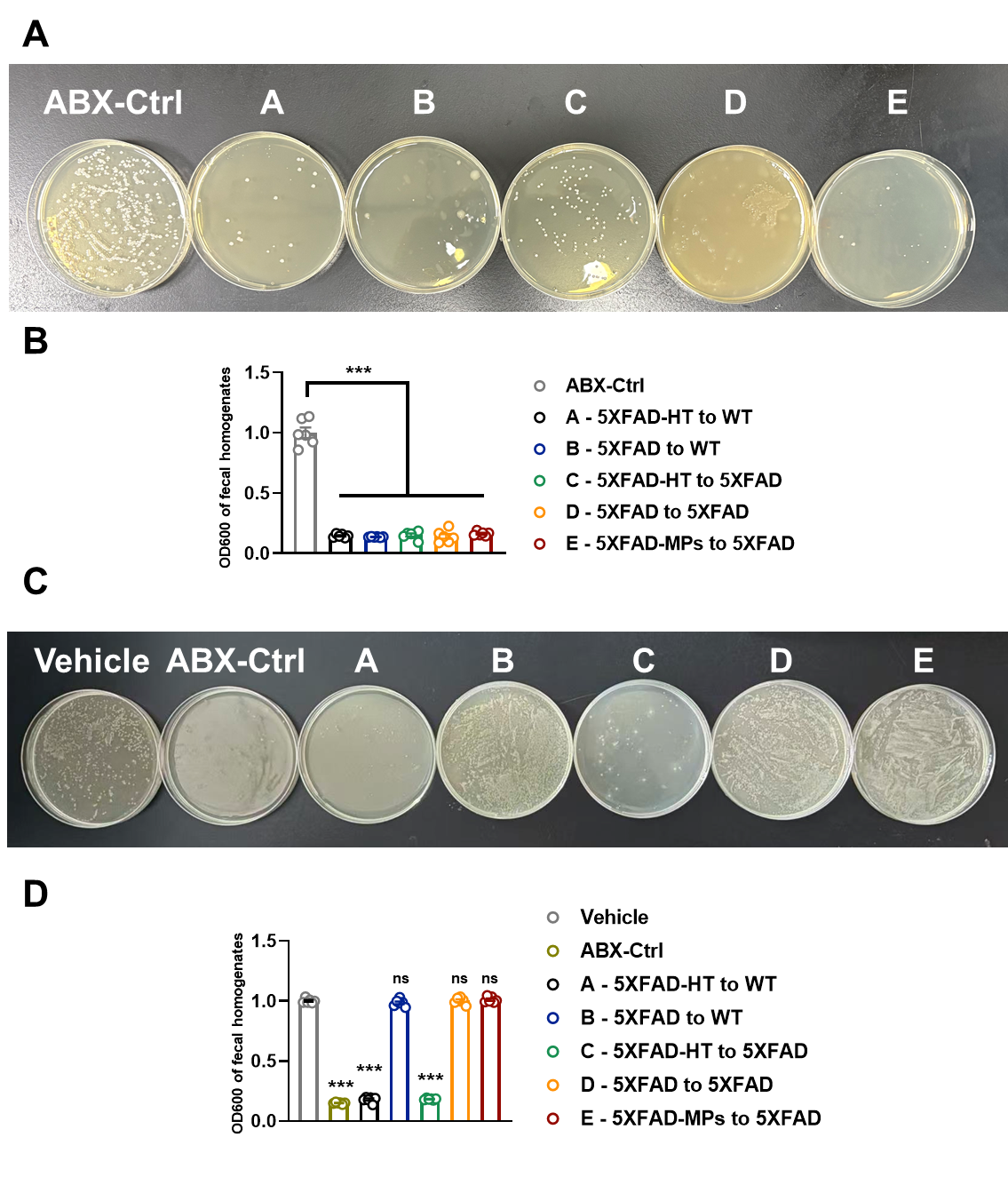


**Fig. S15. Validation of gut microbiota depletion and recolonization following ABX treatment and fecal microbiota transplantation (FMT).** (A) Representative colony-forming unit (CFU) assay image of fecal homogenates from 5XFAD recipient mice following ABX treatment, showing markedly suppressed bacterial growth on Luria-Bertani (LB) agar plates. (B) Quantification of OD_600_ of fecal homogenates confirming a significant reduction in total bacterial load after ABX administration (n = 6 per group). (C) Representative CFA image of fecal homogenates from recipient mice post-FMT, demonstrating a robust restoration of bacterial growth. (D) Corresponding OD_600_ measurements showing significant increase in bacterial abundance following FMT, indicating successful microbial colonization (n = 6 per group). Each data point represents an individual mouse. Data are presented as mean ± SEM. ns, not significant; ****P* < 0.001 (one-way ANOVA with Tukey’s *post hoc* test in B and D).

**Fig. S16.**

**
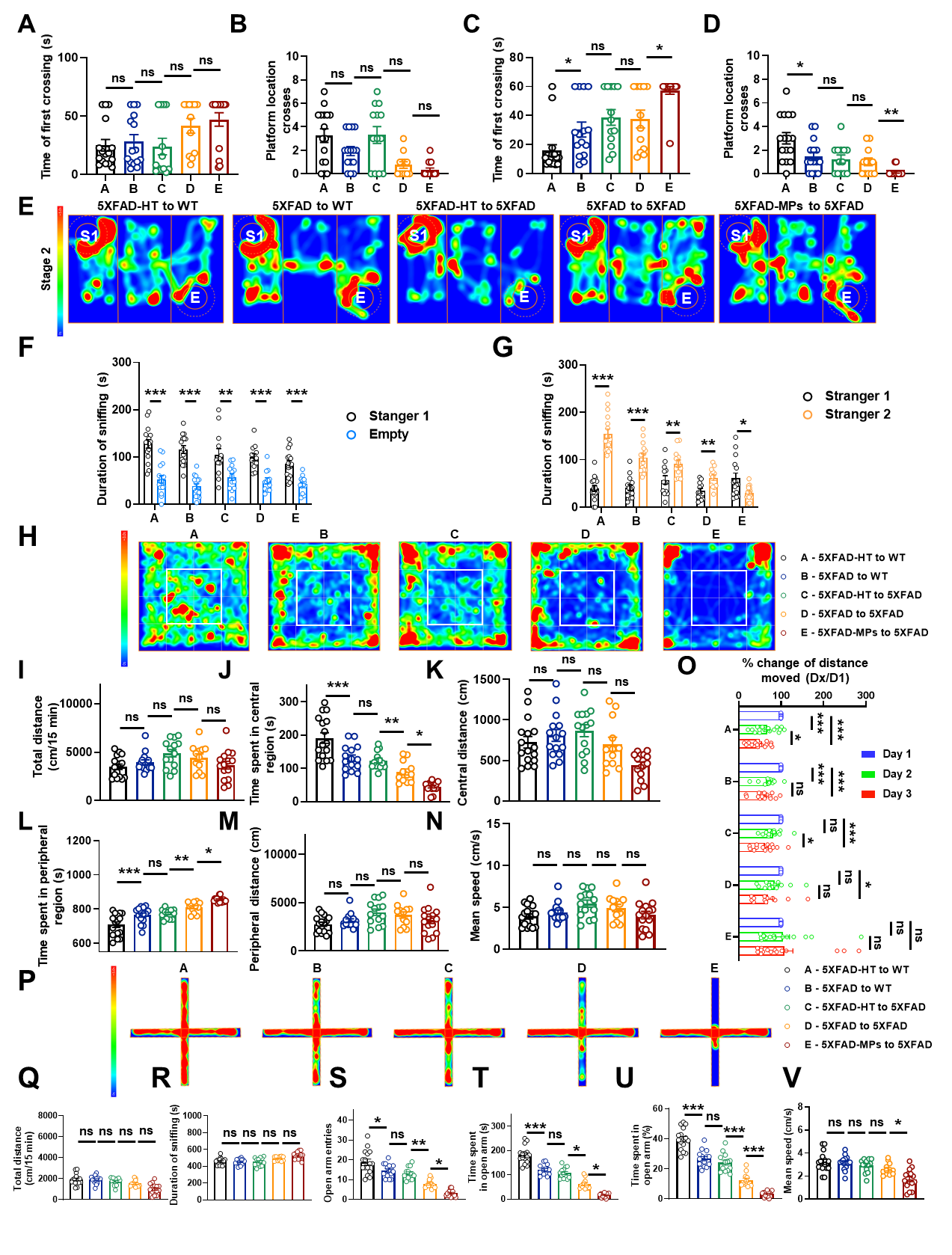
**

**Fig. S16. Supplementary behavioral assessments of MPs-exacerbated deficits in 5XFAD mice.** (A-D) MWM probe trial analysis: (A-B) 4 h probe trial and (C-D) 72 h probe trial, showing (A, C) latency to first platform crossing (s) and (B, D) number of platform location crossings. (E) Representative heatmaps from Stage 2 of the TCST for each group. (F-G) TCST social approach and social novelty preference: (F) sniffing duration toward stranger mouse 1 (S1) versus empty cage, and (G) sniffing duration toward S1 versus stranger mouse 2 (S2). (H) Representative heatmaps and track plots from the OF test for each group. (I-N) OF test parameters: (I) total distance (cm/15 min), (J) time spent in the central region, (K) central distance (cm), (L) time spent in the peripheral region (s), (M) peripheral distance (cm) and (N) mean speed (cm/s). (O) Analysis of locomotor habituation in the OF test over three consecutive days. The graph shows the percent reduction in total distance traveled on Day 2 and Day 3 relative to Day 1 for each experimental group. (P) Representative heatmaps and track plots from the EPM test for each group. (Q-V) EPM test parameters: (Q) total distance traveled, (R) duration of sniffing, (S) open arm entries, (T) time spent in the open arm (s), (U) time spent in the open arm (%) and (V) mean speed. The group sizes were n = 16 (8 males and 8 females) for Group A, n = 15 (8 males and 7 females) for Group B, n = 13 (7 males and 6 females) for Group C, n = 12 (6 males and 6 females) for Group D, and n = 15 (8 males and 7 females) for Group E. Each data point represents an individual mouse. Data are presented as mean ± SEM. ns, not significant; **P* < 0.05, ***P* < 0.01, ****P* < 0.001 (Unpaired Student’s t-test in F and G; one-way ANOVA with Tukey’s *post hoc* test in A-D, I-N, O, Q-V).

**Fig. S17.**


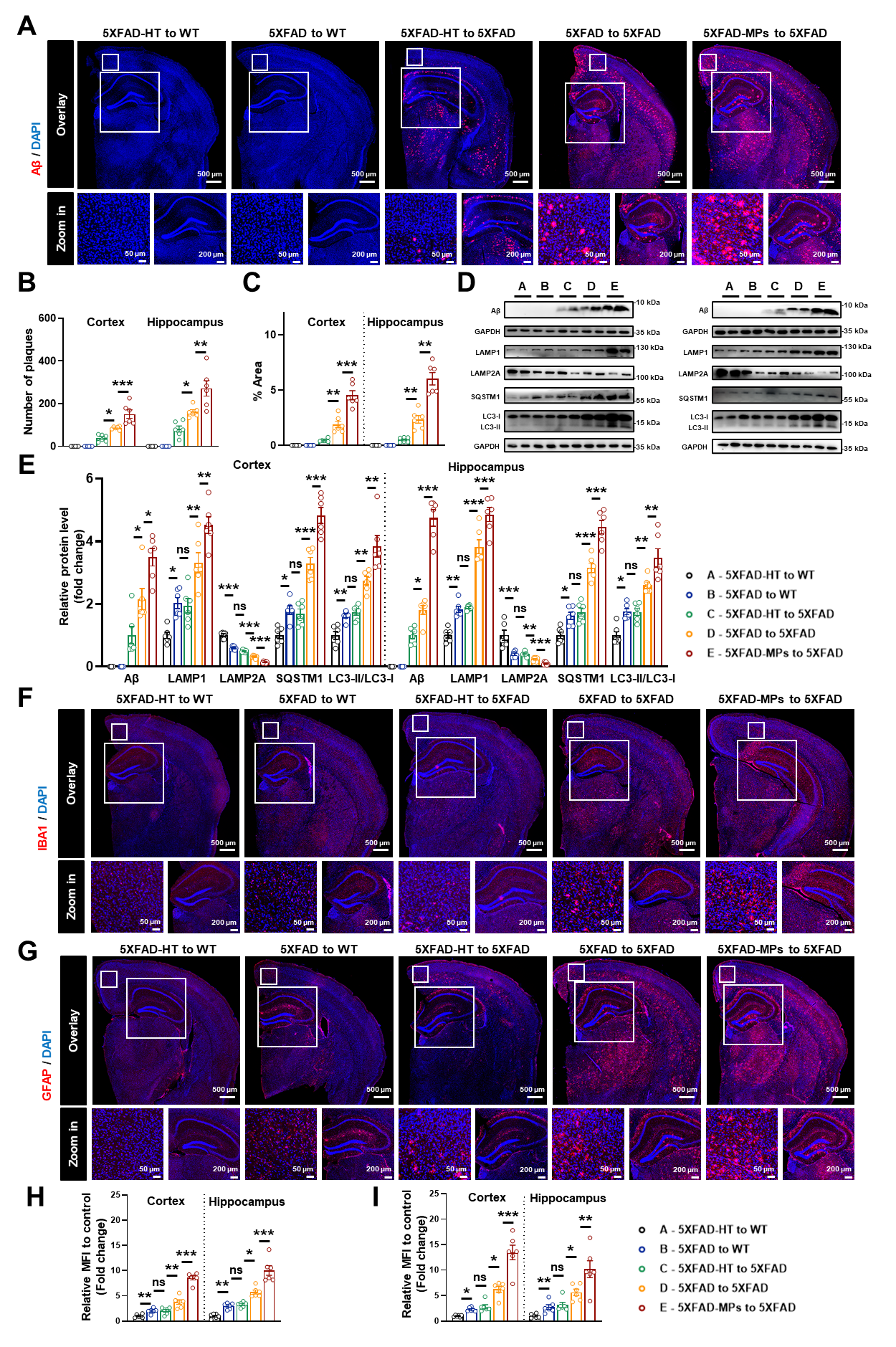


**Fig. S17 Gut microbiota is required for MPs-induced Aβ pathology and gliosis.** (A) Representative cortical and hippocampal immunofluorescence images showing Aβ deposition (red) with DAPI counterstaining (blue) in each group. (B-C) Quantification of Aβ plaque number (B) and plaque area (C) in the cortex and hippocampus. (D) Representative western blots of Aβ and autophagy-related proteins (LAMP1, LAMP2A, SQSTM1, and LC3-II / LC3-I) in cortical and hippocampal tissues. (E) Quantification of the level of Aβ and autophagy-related proteins in D. (F-G) Representative coronal sections co-stained for IBA1 (F) and GFAP (G) with DAPI in the cortex and hippocampus, respectively. (H-I) Quantification of the MFI of IBA1 (H) and GFAP (I) in the cortex and hippocampus among all groups. Each point denotes individual animals. n = 6 biologically independent mice per group. Data are presented as mean ± SEM. ns, not significant; **P* < 0.05, ***P* < 0.01, ****P* < 0.001 (one-way ANOVA with Tukey’s *post hoc* test in B and C, E, H and I).

**Fig. S18.**


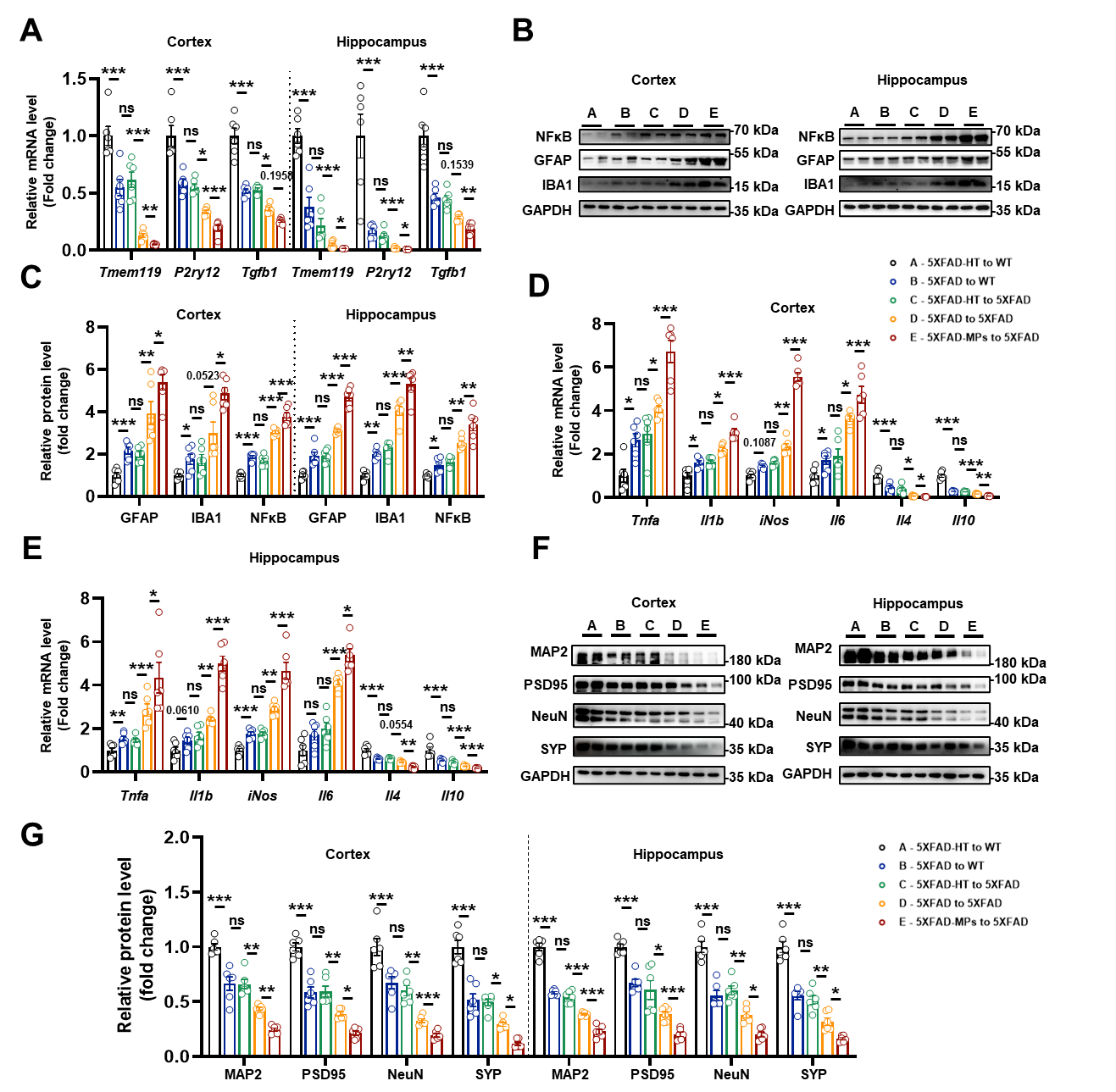


**Fig. S18. FMT from MPs-treated donors exacerbated gliosis, neuroinflammation, and synaptic impairment in recipient mice.** (A) Relative mRNA expression of microglial homeostasis- and phagocytosis-related genes in the cortex and hippocampus across groups. (B-C) Neuroinflammation and glial activation markers: (B) representative western blot images and (C) quantitative analysis of protein levels in the cortex and hippocampus. (D-E) Cytokine expression analysis: relative mRNA levels of pro-inflammatory and anti-inflammatory cytokines in the (D) cortex and (E) hippocampus. (F-G) Neuronal and synaptic integrity: (F) representative western blot images and (G) quantitative analysis of protein levels in the cortex and hippocampus. n = 6 per group. Each data point represents an individual mouse. Data are presented as mean ± SEM. ns, not significant; **P* < 0.05, ***P* < 0.01, ****P* < 0.001 (one-way ANOVA with Tukey’s *post hoc* test in A, C, D and E, G).

**Fig. S19.**


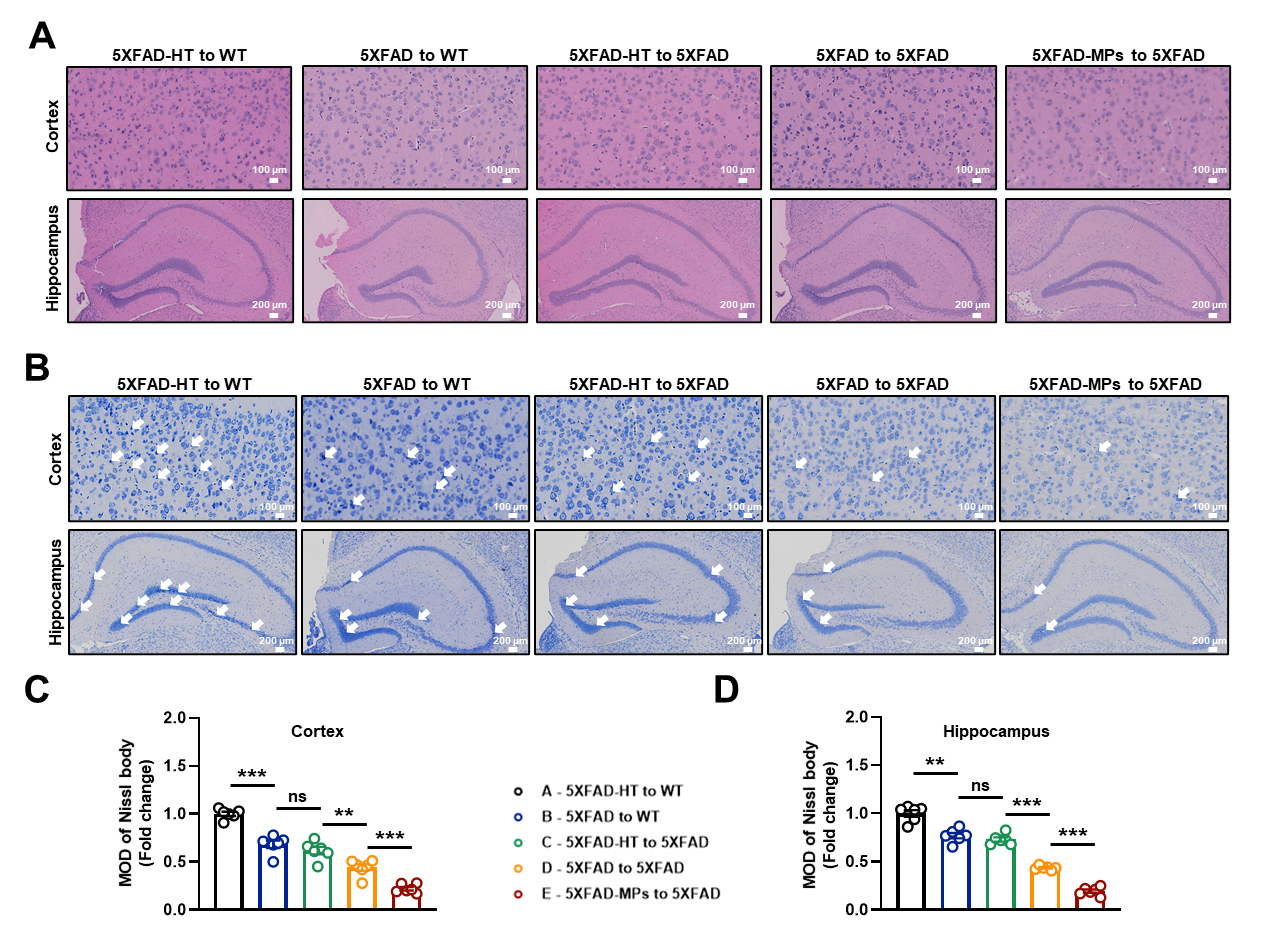


**Fig. S19. FMT from MPs-treated donors exacerbated neuronal impairment in recipient mice.** (A) Representative H&E staining images of the cortex and hippocampus for each group. (B) Representative Nissl staining images of the cortex and hippocampus, with white arrows indicate Nissl bodies. (C-D) Quantification of the MOD of Nissl bodies in the (C) cortex and (D) hippocampus. n = 6 per group. Each data point represents an individual mouse. Data are presented as mean ± SEM. ns, not significant; ***P* < 0.01, ****P* < 0.001 (one-way ANOVA with Tukey’s *post hoc* test in C and D).

**Fig. S20.**


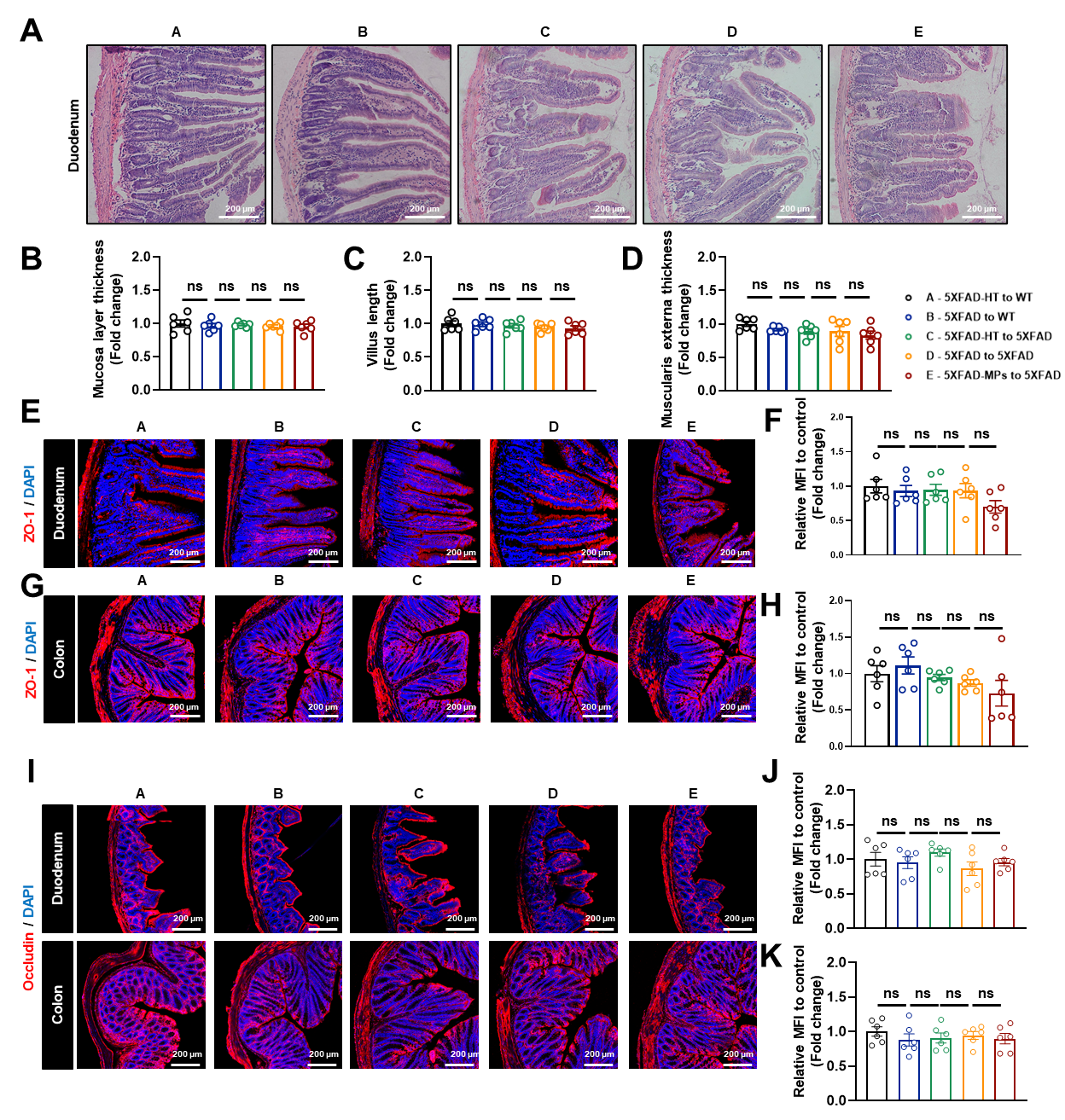


**Fig. S20. FMT from donor mice did not alter the intestinal structure of recipient mice.** (A) Representative H&E images of duodenum and colon of mice in each group. (B-D) Quantitative analysis of the (B) mucosal layer thickness, (C) villus length and (D) muscularis externa thickness of the duodenum. (E-F) Representative fluorescence images of ZO-1 in the duodenum and (F) quantitative analysis and comparison of the MFI of ZO-1 among the three groups. (G-H) Representative fluorescence images of ZO-1 in the colon and (H) quantitative analysis and comparison of the MFI of ZO-1 among the three groups. (I-K) Representative fluorescence images of Occludin in the colon and quantitative analysis and comparison of the MFI of Occludin among the three groups. n = 6. Each point represents a mouse. Data are presented as mean ± SEM. ns, not significant (one-way ANOVA with Tukey’s *post hoc* test in B-D, F and H).

**Fig. S21.**


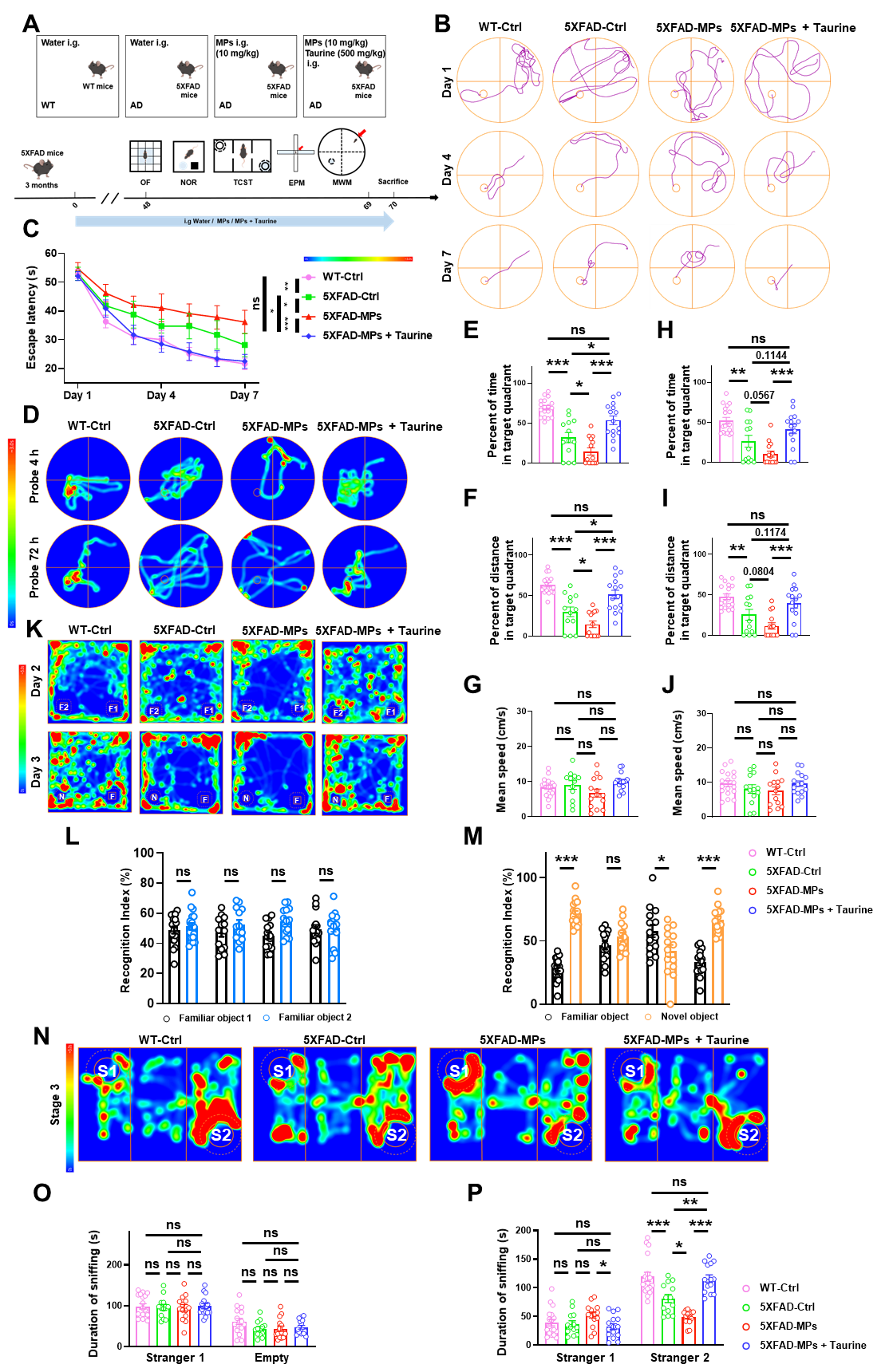


**Fig. S21. Taurine supplementation** **alleviated MPs-exacerbated cognitive and social behavioral deficits in 5XFAD mice.** (A) Schematic illustration of experimental design and group allocation. Mice received differential treatments for the same duration, followed by comprehensive behavioral assessments. (B) Representative track plots from MWM test for each group in the training session. (C) MWM navigation training showed escape latency across consecutive training days. (D) Representative heatmaps from the MWM test in 4 h and 72 h probe trials for each group. (E-G) Probe trial analysis (4 h post-training): (E) percentage of time in the target quadrant, (F) percentage of distance in the target quadrant, and (G) mean swimming speed (cm/s). (H-J) Probe trial analysis (72 h post-training): (H) percentage of time in the target quadrant, (I) percentage of distance in the target quadrant, and (J) mean swimming speed (cm/s). (K) Representative heatmaps from the NOR test for each group. (L-M) NOR performance: (L) recognition index (%) on day 2 (familiar object pairs) and (M) recognition index (%) on day 3 (novel vs. familiar object). (N) Representative heatmaps from stage 3 of the TCST for each group. (O-P) TCST social behavior analysis: (O) sniffing duration (s) toward empty cage (E) or stranger mouse 1 (S1), and (P) sniffing duration (s) toward S1 or stranger mouse 2 (S2). The group sizes were n = 18 (9 males and 9 females), n = 13 (7 males and 6 females), n = 14 (7 males and 7 females), and n = 15 (8 males and 7 females) for the four groups, respectively. Each data point represents an individual mouse. Data are presented as mean ± SEM. ns, not significant; **P* < 0.05, ***P* < 0.01, ****P* < 0.001 (unpaired Student’s t-test in L and M; one-way ANOVA with Tukey’s *post hoc* test in E-J, O and P; Two-way ANOVA with Tukey’s *post hoc* test in C).

**Fig. S22.**


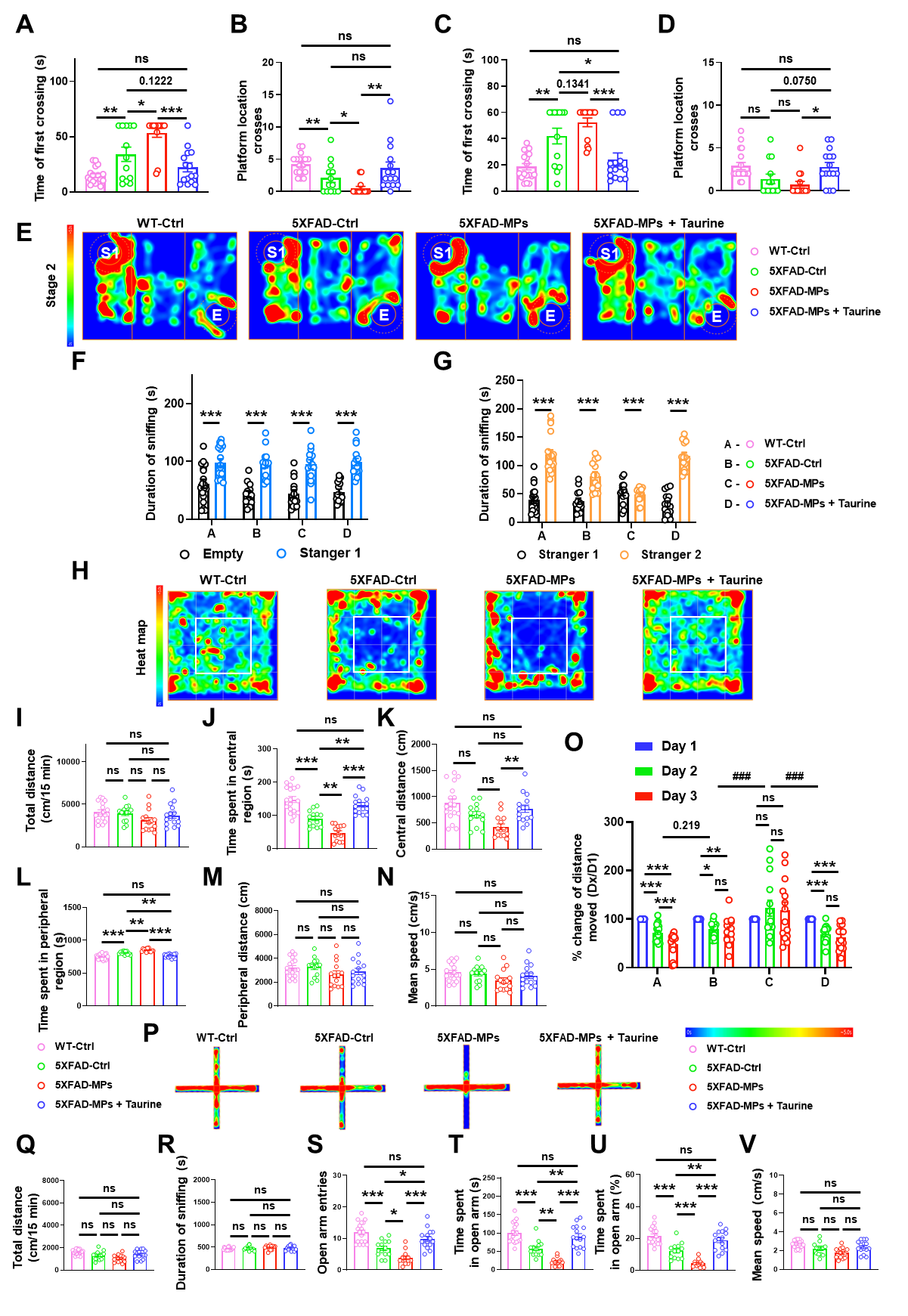


**Fig. S22. Taurine administration alleviated MPs-exacerbated behavioral deficits in 5XFAD mice.** (A-D) MWM probe trial analysis: (A-B) 4 h probe trial and (C-D) 72 h probe trial showing (A, C) time of the first crossing (s) and (B, D) platform location crosses. (E) Representative heatmaps from stage 2 of the TCST for each group. (F-G) TCST social approach and social novelty preference: (F) duration of sniffing (s) toward stranger mouse 1 (S1) versus an empty cage, and (G) duration of sniffing (s) toward S1 versus stranger mouse 2 (S2). (H) Representative heatmaps and track plots from the OF test for each group. (I-N) OF test parameters: (I) total distance (cm/15 min), (J) time spent in the central region, (K) central distance (cm), (L) time spent in the peripheral region (s), (M) peripheral distance (cm) and (N) mean speed (cm/s). (O) Analysis of locomotor habituation in the open field test over three consecutive days, showing percent reduction in total distance traveled on Days 2 and 3 relatives to Day 1. (P) Representative heatmaps and track plots from the EPM test for each group. (Q-V) EPM test parameters: (Q) total distance, (R) duration of sniffing (s), (S) open arm entries, (T) time spent in the open arm (s), (U) time spent in the open arm (%) and (V) mean speed. The group sizes were n = 18 (9 males and 9 females), n = 13 (7 males and 6 females), n = 14 (7 males and 7 females), and n = 15 (8 males and 7 females) for the four groups, respectively. Each data point represents an individual mouse. Data are presented as mean ± SEM. ns, not significant; **P* < 0.05, ***P* < 0.01, ****P* < 0.001 (unpaired Student’s *t*-test in F and G; one-way ANOVA with Tukey’s *post hoc* test in A-D, I-N, O, Q-V; Two-way ANOVA with Tukey’s *post hoc* test in O).

**Fig. S23.**


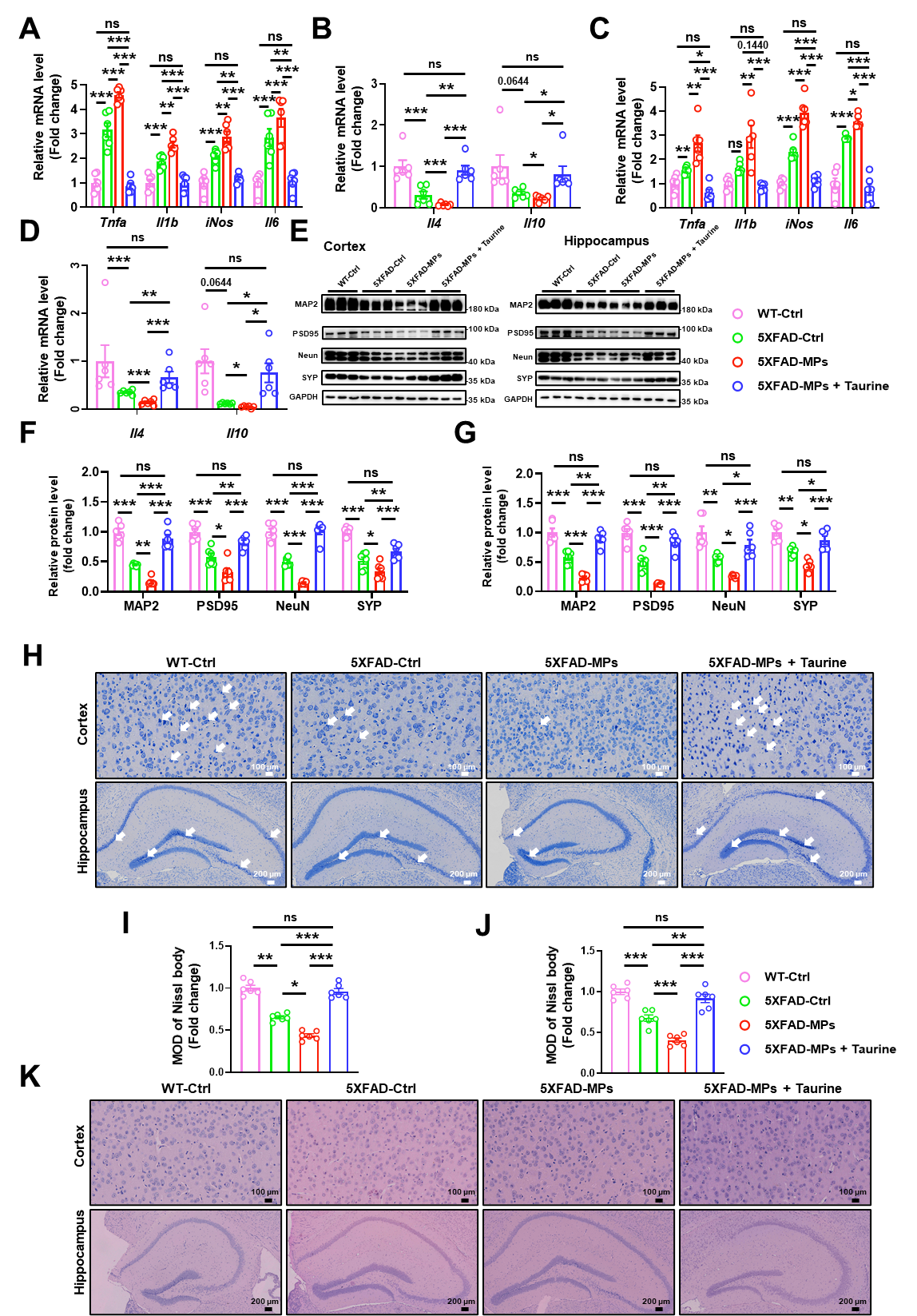


**Fig. S23. Taurine supplementation alleviated MPs-exacerbated synaptic impairment and neuroinflammation in 5XFAD mice.** (A-D) Relative mRNA expression of cytokines in the cortex and hippocampus: (A-B) pro-inflammatory and anti-inflammatory cytokines in the cortex, and (C-D) corresponding quantitative analysis in the hippocampus. (E-G) Neuronal and synaptic integrity markers: (E) representative western blot images and quantitative analysis of protein levels in the (F) cortex and (G) hippocampus. (H) Representative Nissl staining images of the cortex and hippocampus, with white arrows indicating Nissl bodies. (I-J) Quantification of the MOD of Nissl bodies in the (I) cortex and (J) hippocampus. (K) Representative H＆E staining images of the cortex and hippocampus for each group. n = 6 per group. Each data point represents an individual mouse. Data are presented as mean ± SEM. ns, not significant; **P* < 0.05, ***P* < 0.01, ****P* < 0.001 (one-way ANOVA with Tukey’s *post hoc* test A-D, F and G, J and K).

**Fig. S24.**


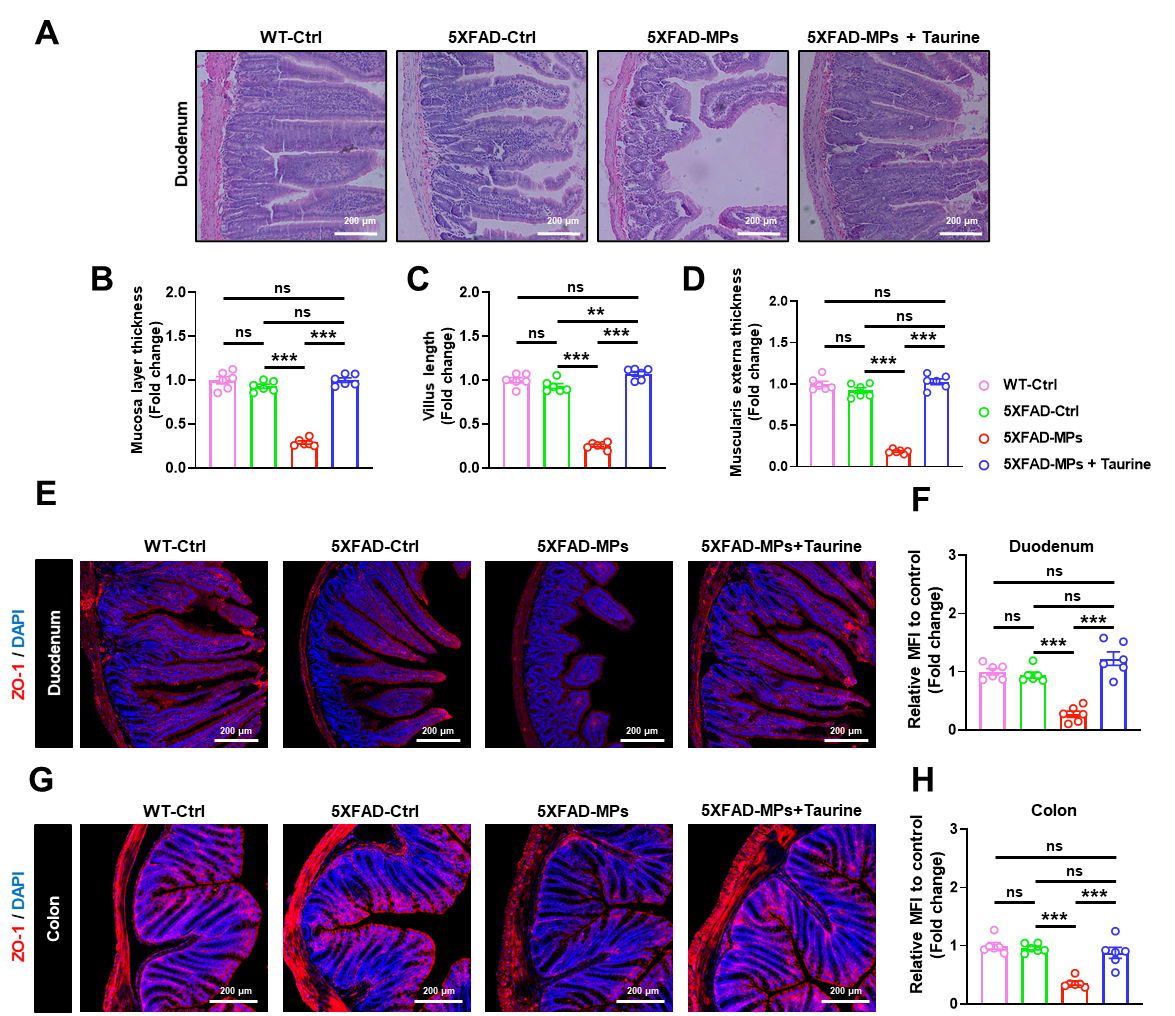


**Fig. S24. Taurine supplementation alleviates MPs-exacerbated intestinal pathology of 5XFAD mice.** (A) Representative H＆E images of the duodenum of mice in each group. (B-D) Quantitative analysis of the (B) mucosal layer thickness, (C) villus length and (D) muscularis externa thickness of the duodenum. (E-F) Representative fluorescence images of ZO-1 in the duodenum and (F) quantitative analysis and comparison of the MFI of ZO-1 among the three groups. (G-H) Representative fluorescence images of ZO-1 in the colon and (H) quantitative analysis and comparison of the MFI of ZO-1 among the three groups. n = 6. Each point represents a mouse. Data are presented as mean ± SEM. ns, not significant (one-way ANOVA with Tukey’s *post hoc* test in B-D, F and H).

**Fig. S25.**


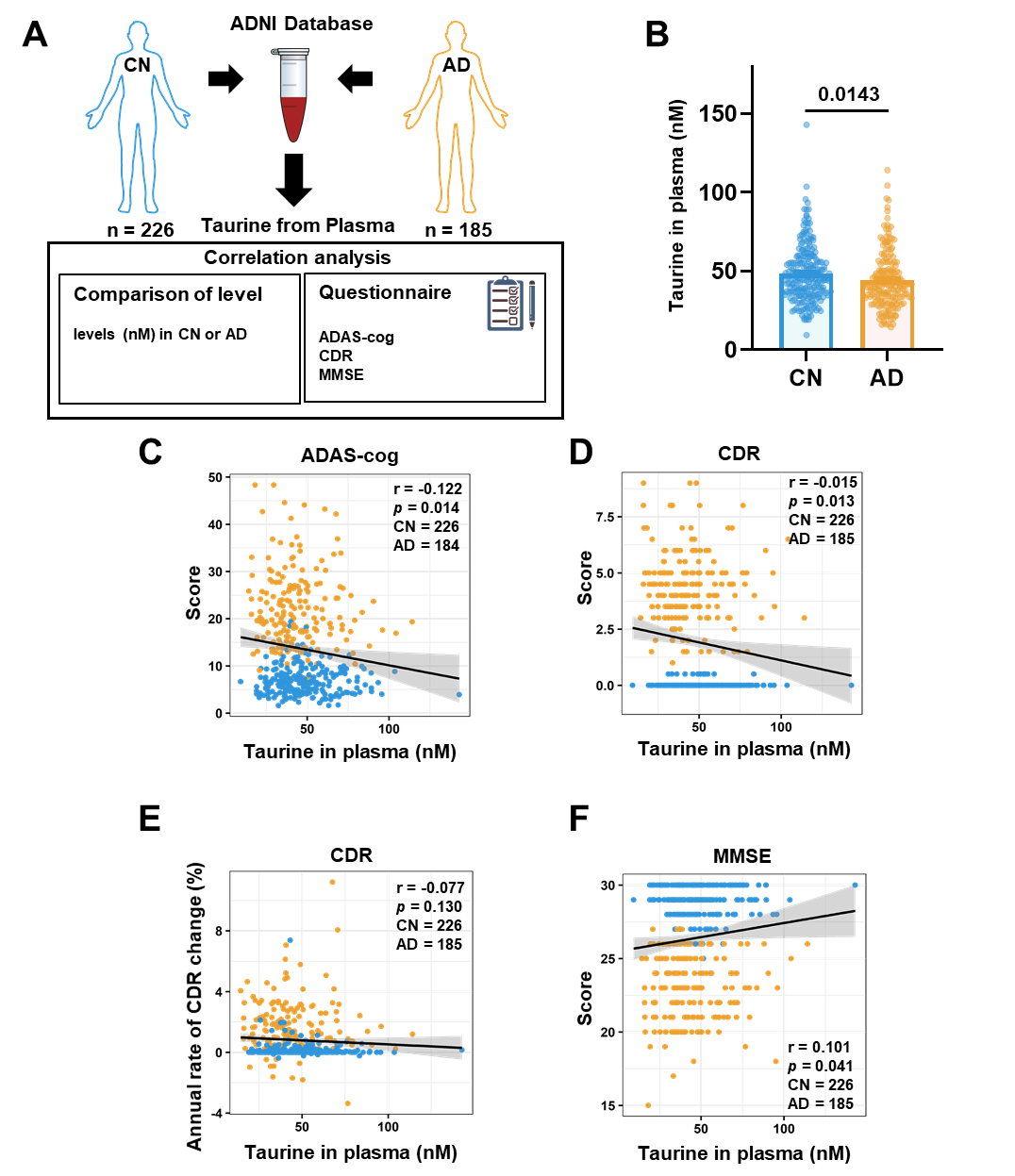


**Fig. S25. Decreased plasma taurine level in patients with AD.** (A) Schematic overview of the study design linking plasma taurine levels to clinical status and cognitive performance in the Alzheimer's Disease Neuroimaging Initiative (ADNI) cohort. (B) Plasma taurine concentration in cognitively normal (CN) controls versus AD patients. (C-F) Correlation analyses between plasma taurine levels and cognitive assessment scores: (C) Alzheimer's Disease Assessment Scale-Cognitive Subscale (ADAS-cog), (D-E) Clinical Dementia Rating (CDR) and (F) Mini-Mental State Examination (MMSE). Pearson correlation coefficients (r) and *P* values are indicated. Each data point represents an individual participant. Data are presented as mean ± SEM.

**Table S1** KEGG pathway enrichment results of the serum metabolomics

| **ID** | **KEGG Pathway** | **Adjusted *P* value** |
| --- | --- | --- |
| mmu00260 | Glycine, serine and threonine metabolism | 2.77034e-06 |
| mmu00052 | Galactose metabolism | 1.90034e-07 |
| mmu00240 | Pyrimidine metabolism | 8.65057e-06 |
| mmu00330 | Arginine and proline metabolism | 1.78826e-05 |
| mmu00970 | Aminoacyl-tRNA biosynthesis | 7.81681e-08 |
| mmu04974 | Protein digestion and absorption | 1.45138e-10 |
| mmu05230 | Central carbon metabolism in cancer | 1.62501e-13 |
| mmu02010 | ABC transporters | 2.48727e-06 |
| mmu04721 | Synaptic vesicle cycle | 0.006485513 |
| mmu04728 | Dopaminergic synapse | 0.006485513 |
| mmu05031 | Amphetamine addiction | 0.003715807 |
| mmu05034 | Alcoholism | 0.003715807 |
| mmu05231 | Choline metabolism in cancer | 0.004985082 |
| mmu00430 | Taurine and hypotaurine metabolism | 0.007602902 |
| mmu04918 | Thyroid hormone synthesis | 0.004627844 |
| mmu00030 | Pentose phosphate pathway | 0.007205186 |
| mmu00480 | Glutathione metabolism | 0.008078715 |
| mmu00770 | Pantothenate and CoA biosynthesis | 0.002840161 |
| mmu04148 | Efferocytosis | 0.000516542 |
| mmu00053 | Ascorbate and aldarate metabolism | 0.011273304 |
| mmu00220 | Arginine biosynthesis | 0.000103161 |
| mmu00500 | Starch and sucrose metabolism | 0.001246238 |
| mmu04082 | Neuroactive ligand signaling | 0.001895262 |
| mmu05415 | Diabetic cardiomyopathy | 0.001655699 |
| mmu00051 | Fructose and mannose metabolism | 0.002138541 |
| mmu00250 | Alanine, aspartate and glutamate metabolism | 2.64767E-05 |
| mmu00270 | Cysteine and methionine metabolism | 0.007129955 |
| mmu00400 | Phenylalanine, tyrosine and tryptophan biosynthesis | 0.000123649 |
| mmu00564 | Glycerophospholipid metabolism | 0.002377198 |
| mmu04080 | Neuroactive ligand-receptor interaction | 0.000317663 |

**Table S2** Top 50 VIP metabolites in Cluster 4 of serum

| **Metabolite** | **VIP** | **Metabolite** | **VIP** |
| --- | --- | --- | --- |
| Isorosmanol | 1.94398545 | Citrulline | 1.245707349 |
| Ciclopirox | 1.77637499 | LPC(16:0) | 1.244887591 |
| Sulfamerazine | 1.553600132 | 2-(Diphenylphosphoryl)-1,4-benzenediol | 1.243147383 |
| 2,3-Dinorfluprostenol | 1.513416142 | Leucopterin | 1.241155423 |
| Asticolorin_B | 1.490443899 | Tryptophan | 1.239730958 |
| 4-Chloro-N-(2,3-dimethylphenyl)benzenesulfonamide | 1.445590466 | Diglycine | 1.236348428 |
| N-Acetylsulfamethoxazole | 1.437704583 | 1-Stearoyl-2-arachidonoyl-sn-glycero-3-phospho-(1'-myo-inositol) | 1.234693284 |
| Ganoderic_acid_L | 1.409476894 | Glyoxylic acid | 1.234579492 |
| 1-Butanone, 1-(1,3-benzodioxol-5-yl)-2-(dimethylamino)- | 1.396855875 | Purine mononucleotide | 1.232512429 |
| 4-Aminobutyric acid (GABA) | 1.389716079 | 2-(1-Hydroxycyclohexyl)butanoic acid | 1.230754444 |
| Trichodermamide C | 1.382475157 | Pergolide | 1.227241601 |
| (R)-S-Lactoylglutathione | 1.364373384 | M544T208 | 1.217347875 |
| Acamprosate (calcium) | 1.341283407 | L-NG-Monomethylarginine | 1.203244452 |
| 3-Dehydro-L-threonate | 1.334928875 | Dimethyltin oxide | 1.193764101 |
| 6-[3-[(3,4-dimethoxyphenyl)methyl]-4-methoxy-2-(methoxymethyl)butyl]-4-methoxy-1,3-benzodioxole | 1.320947582 | M131T147 | 1.18637338 |
| UDP-N-acetyl-D-mannosaminouronate | 1.308586583 | Sulfamethoxazole | 1.179733433 |
| Glu-Arg | 1.306968033 | Arginine | 1.300887098 |
| Taurine | 1.179319613 | 2-Acetylthiazole | 1.173274894 |
| Aminocyclopyrachlor | 1.176452787 |  |  |
| 3-(Diphenylphosphino)propionic acid | 1.173585458 |  |  |

**Table S3** Top 50 VIP metabolites in Cluster 4 of cecal contents

| **Metabolite** | **VIP** | **Metabolite** | **VIP** |
| --- | --- | --- | --- |
| Secologanoside | 2.047096975 | Cellobiose | 1.753194558 |
| Melibiose | 1.919450763 | M270T173 | 1.739856918 |
| 2-Deoxyribose 5-phosphate | 1.871130917 | M328T173 | 1.711101962 |
| M350T216 | 1.834819711 | 6-Benzyl-3-butan-2-yl-9-(7,8-dihydroxy-6-oxooctyl)-1,4,7,10-tetrazabicyclo[10.4.0]hexadecane-2,5,8,11-tetrone | 1.706568765 |
| M524T174 | 1.827154363 | Neohancoside D | 1.685153177 |
| M196T183 | 1.823980395 | M497T42 | 1.683694015 |
| N-Butyl-2-(2,4-dichlorophenoxy)propanamide | 1.818261665 | M441T183 | 1.676931325 |
| CDP-ethanolamine | 1.807013574 | Murrastifoline_F | 1.676814122 |
| Gulono-1,4-lactone | 1.801331074 | 4-Ethoxy-4-oxobut-2-enoic acid | 1.670894219 |
| Taurine | 1.789265334 | Quazepam | 1.662853129 |
| Calphostin C | 1.777113707 | M476T191 | 1.621620075 |
| 1-(3-Chlorophenyl)-3-[1-(3,4-dichlorobenzyl)-1H-pyrazol-3-yl]urea | 1.771459987 | PE(18:0/18:2) | 1.61217466 |
| 2-{[2-Chloro-5-(trifluoromethyl)anilino]carbonyl}benzoic acid | 1.771231621 | M402T207 | 1.611815765 |
| Isomaltose | 1.753194558 | Benzo[b]thiophene-2-carboxamide, 3-chloro-N-[trans-4-(methylamino)cyclohexyl]-N-[[3-(4-pyridinyl)phenyl]methyl]- | 1.58655432 |
| N-Formylaspartate | 1.576962658 |  |  |
| 6-(3,4-Dihydro-2(1H)-isoquinolinyl)-3-methyl[1,2,4]triazolo[3,4-a]phthalazine | 1.542058942 | M543T173 | 1.487318504 |
| Swertiamarin | 1.53540505 | 2-(S-Glutathionyl)acetyl glutathione | 1.478881856 |
| 2,4-Dihydroxybutanoic acid | 1.533017399 | 2-{[2-(3,4-Dihydroxyphenyl)-2-oxoethyl]sulfanyl}-6-methyl-4(3H)-pyrimidinone | 1.447921213 |
| [(6R,7R)-7-Hydroxy-7-methyl-8-oxo-3-[(E)-prop-1-enyl]-5,6-dihydro-1H-isochromen-6-yl] 3,6-dihydroxy-4-methoxy-2-methylbenzoate | 1.542969299 | Methanone, (2-iodophenyl)(1-pentyl-1H-indol-3-yl)- | 1.437932561 |
| Erianin | 1.523157442 | clemastine | 1.434322436 |
| 3-Oxocyclobutanecarboxylic acid | 1.52150047 | LPA(20:5) | 1.424950673 |
| Pyrogallol | 1.509146463 | Coagulin_R_3-glucoside | 1.420223808 |
| Propachlor ESA | 1.507074552 | Deacetylnomilin | 1.411304594 |
| Pyruvaldehyde | 1.50349434 | Arachidonoyl ethanolamide phosphate | 1.39683261 |
| 1-O-(2,3,19,23-Tetrahydroxy-23,28-dioxours-12-en-28-yl)hexopyranose | 1.502669541 | Piericidin C1 | 1.393763643 |
| Mannose | 1.488377298 |  |  |

**Table S4** KEGG pathway enrichment results of the cecal contents metabolomic

| **ID** | **KEGG Pathway** | **Adjusted *P* value** |
| --- | --- | --- |
| mmu00020 | Citrate cycle (TCA cycle) | 0.004268483 |
| mmu00052 | Galactose metabolism | 4.17979E-08 |
| mmu00260 | Glycine, serine and threonine metabolism | 0.001146375 |
| mmu00500 | Starch and sucrose metabolism | 0.003216978 |
| mmu00520 | Amino sugar and nucleotide sugar metabolism | 0.001283792 |
| mmu00630 | Glyoxylate and dicarboxylate metabolism | 0.000814308 |
| mmu02010 | ABC transporters | 1.50433E-05 |
| mmu04974 | Protein digestion and absorption | 0.001040351 |
| mmu04981 | Folate transport and metabolism | 0.004268483 |
| mmu05230 | Central carbon metabolism in cancer | 1.92795E-06 |

**Table S5.** Antibodies used in this study.

| **Primary antibodies** | **Source** | **Catalog no.** | **Western blot** | **Immunofluorescence** | **Immunohistochemistry** |
| --- | --- | --- | --- | --- | --- |
| Rabbit polyclonal anti-GAPDH | Affinit | AF7021 | 1:5000 |  |  |
| Rabbit monoclonal Anti-Aβ | Cell signaling | 8243s | 1:1000 | 1:500 | 1:500 |
| Rabbit monoclonal Anti-NFκB | Cell signaling | 8242P | 1:1000 |  |  |
| Rabbit polyclonal Anti-GFAP | Servicebio | GB11096 | 1:3000 | 1:500 | 1:500 |
| Rabbit polyclonal Anti-Caspase 3 | Cell signaling | 9662S | 1:1000 |  |  |
| Rabbit polyclonal Anti-IBA1 | Bioss | bs-1363R | 1:400 |  |  |
| Rabbit polyclonal Anti-IBA1 | Wako | 019-19741 |  | 1:500 | 1:500 |
| Rabbit recombinant multiclonal Anti-MAP2 | Abcam | ab281588 | 1:1000 |  |  |
| Rabbit polyclonal Anti-PSD95 | Abcam | ab18258 | 1:1000 |  |  |
| Rabbit monoclonal Anti-NeuN | Abcam | ab177487 | 1:1000 |  |  |
| Rabbit polyclonal anti-synaptophysin | Cell signaling | AF0257 | 1:1000 |  |  |
| Rabbit polyclonal Anti-LAMP1 | Abcam | ab24170 | 1:1000 |  |  |
| Rabbit polyclonal Anti-LAMP2A | Abcam | ab18528 | 1:1000 |  |  |
| Rabbit monoclonal Anti-SQSTM1 | ABclonal | A19700 | 1:1000 |  |  |
| Rabbit polyclonal Anti-LC3 | Proteintech | 14600-1-AP | 1:1000 |  |  |
| Rabbit polyclonal Anti-ZO-1 | Proteintech | 21773-1-AP |  | 1:200 |  |
| Rabbit polyclonal Anti-Occludin | Affinit | DF7504 |  | 1:200 |  |
| KPL peroxidase-labeled affinity purified antibody to rabbit IgG (H + L) | Seracare | 5450-0010 (474-1506) | 1:10000 |  | 1:400 |
| CoraLite594–conjugated Goat Anti-Rabbit IgG(H+L) | Proteintech | SA00013-4 |  | 1:400 |  |

**Table S6.** Primer sequences for RT-qPCR in this study^19^

| REAGENT or RESOURCE | SOURCE | Cat |
| --- | --- | --- |
| ***Gapdh*** F 5-TGGAGAAACCTGCCAAGTATGA-3' | This Study | N/A |
| ***Gapdh*** R 5-TGGAAGAATGGGAGTTGCTGT-3' | This Study | N/A |
| ***Tmem119*** F 5-CCTACTCTGTGTCACTCCCG-3' | This Study | N/A |
| ***Tmem119*** R 5-CACGTACTGCCGGAAGAAATC-3' | This Study | N/A |
| ***P2ry12*** F 5-ATGGATATGCCTGGTGTCAACA-3' | This Study | N/A |
| ***P2ry12*** R 5-AGCAATGGGAAGAGAACCTGG-3' | This Study | N/A |
| ***Tgfb1*** F 5-CTCCCGTGGCTTCTAGTGC-3' | This Study | N/A |
| ***Tgfb1*** R 5-GCCTTAGTTTGGACAGGATCTG-3' | This Study | N/A |
| ***inos*** F 5-GGAGTGACGGCAAACATGACT-3' | This Study | N/A |
| ***inos*** R 5-TCGATGCACAACTGGGTGAAC-3' | This Study | N/A |
| ***Il1b*** F 5-TGCCACCTTTTGACAGTGATG-3' | This Study | N/A |
| ***Il1b*** R 5-TGATGTGCTGCTGCGAGATT-3' | This Study | N/A |
| ***Il6*** F 5-TAGTCCTTCCTACCCCAATTTCC-3' | This Study | N/A |
| ***Il6*** R 5-TTGGTCCTTAGCCACTCCTTC-3' | This Study | N/A |
| ***Tnfa*** F 5-CTCATGCACCACCATCAAGG-3' | This Study | N/A |
| ***Tnfa*** R 5-ACCTGACCACTCTCCCTTTG-3' | This Study | N/A |
| ***Il4*** F 5-GGTCTCAACCCCCAGCTAGT-3' | This Study | N/A |
| ***Il4*** R 5-GCCGATGATCTCTCTCAAGTGAT-3' | This Study | N/A |
| ***Il10*** F 5-GCTCTTACTGACTGGCATGAG-3' | This Study | N/A |
| ***Il10*** R 5-CGCAGCTCTAGGAGCATGTG-3' | This Study | N/A |

**Reference**

1 Pan, R. Y. *et al.* Intermittent fasting protects against Alzheimer's disease in mice by altering metabolism through remodeling of the gut microbiota. ***Nat Aging*** 2, 1024-1039, doi:10.1038/s43587-022-00311-y (2022).

2 Wang, J. *et al.* The enhancement in toxic potency of oxidized functionalized polyethylene-microplastics in mice gut and Caco-2 cells. ***Sci Total Environ*** 903, 166057, doi:10.1016/j.scitotenv.2023.166057 (2023).

3 Boutron, I. *et al.* The ARRIVE guidelines 2.0: Updated guidelines for reporting animal research. ***PLoS Biol*** 18, doi:10.1371/journal.pbio.3000410 (2020).

4 Cox, K. D. *et al.* Human consumption of microplastics. ***Environ Sci Technol*** 53, 7068-7074, doi:10.1021/acs.est.9b01517 (2019).

5 Yang, Q. Y. *et al.* Oral feeding of nanoplastics affects brain function of mice by inducing macrophage IL-1 signal in the intestine. ***Cell Rep*** 42, 112346, doi:10.1016/j.celrep.2023.112346 (2023).

6 Nair, A. & Jacob, S. A simple practice guide for dose conversion between animals and human. ***J Basic Clin Pharm*** 7, doi:10.4103/0976-0105.177703 (2016).

7 Fabbiano, S. *et al.* Functional gut microbiota remodeling contributes to the caloric restriction-induced metabolic improvements. ***Cell Metab*** 28, 907–921.e907, doi:10.1016/j.cmet.2018.08.005 (2018).

8 Mohr, A. E. *et al.* Gut microbiome remodeling and metabolomic profile improves in response to protein pacing with intermittent fasting versus continuous caloric restriction. ***Nat Commun*** 15, 4155, doi:10.1038/s41467-024-48355-5 (2024).

9 Jin, J. *et al.* Gut-derived beta-amyloid: Likely a centerpiece of the gut-brain axis contributing to Alzheimer's pathogenesis. ***Gut Microbes*** 15, 2167172, doi:10.1080/19490976.2023.2167172 (2023).

10 Singh, P. *et al.* Taurine deficiency as a driver of aging. ***Science*** 380, doi:10.1126/science.abn9257 (2023).

11 Luo, R. C. *et al.* Activation of PPARA-mediated autophagy reduces Alzheimer disease-like pathology and cognitive decline in a murine model. ***Autophagy*** 16, 52-69, doi:10.1080/15548627.2019.1596488 (2020).

12 Wang, J. *et al.* Chlorpyrifos induced dysregulation of arginine biosynthesis pathway aggravates Alzheimer’s disease progression in 5XFAD mice via microbiota-gut-brain axis crosstalk. ***Environ Int*** 208, 110064, doi:10.1016/j.envint.2026.110064 (2026).

13 Luo, R. C. *et al.* A novel missense variant in ACAA1 contributes to early-onset Alzheimer's disease, impairs lysosomal function, and facilitates amyloid-β pathology and cognitive decline. ***Signal Transduct Tar*** 6, 325, doi:10.1038/s41392-021-00748-4 (2021).

14 Livak, K. J. & Schmittgen, T. D. Analysis of relative gene expression data using real-time quantitative PCR and the 2(-Delta Delta C(T)) Method. ***Methods*** 25, 402-408, doi:10.1006/meth.2001.1262 (2001).

15 Zha, X. *et al.* Microbiota-derived lysophosphatidylcholine alleviates Alzheimer’s disease pathology via suppressing ferroptosis. ***Cell Metab*** 37, 169-186.e169, doi:10.1016/j.cmet.2024.10.006 (2025).

16 DeSantis, T. Z. *et al.* Greengenes, a chimera-checked 16S rRNA gene database and workbench compatible with ARB. ***Appl Environ Microbiol*** 72, 5069-5072, doi:10.1128/aem.03006-05 (2006).

17 Quast, C. *et al.* The SILVA ribosomal RNA gene database project: improved data processing and web-based tools. ***Nucleic Acids Res*** 41, D590-D596, doi:10.1093/nar/gks1219 (2012).

18 Weiner, M. W. *et al.* 2014 update of the Alzheimer's disease neuroimaging initiative: A review of papers published since its inception. ***Alzheimers Dement*** 11, e1-120, doi:10.1016/j.jalz.2014.11.001 (2015).

19 Whittaker, D. S. *et al.* Circadian modulation by time-restricted feeding rescues brain pathology and improves memory in mouse models of Alzheimer's disease. ***Cell Metab*** 35, 1704-1721.e1706, doi:10.1016/j.cmet.2023.07.014 (2023).
